# How leisure activities affect health: a narrative review and multi-level theoretical framework of mechanisms of action

**DOI:** 10.1016/S2215-0366(20)30384-9

**Published:** 2021-02-11

**Authors:** Daisy Fancourt, Henry Aughterson, Saoirse Finn, Emma Walker, Andrew Steptoe

**Affiliations:** Department of Behavioural Science & Health, University College London; Department of Behavioural Science & Health, University College London; Department of Behavioural Science & Health, University College London; Department of Epidemiology & Public Health, University College London; Department of Behavioural Science & Health, University College London

## Abstract

There is a large and growing body of evidence on the health benefits of engagement in leisure activities (voluntary, enjoyable non-work activities, such as hobbies, arts, volunteering, community group membership, sports, and socialising). However, there is no unifying framework explaining how leisure activities affect health: what the mechanisms of action are by which engagement with leisure activities leads to the prevention, management, or treatment of mental and physical illness. In this Review, we identify and map over 600 mechanisms of action. These mechanisms can be categorised as psychological, biological, social, and behavioural processes that operate at individual (micro), group (meso), and societal (macro) levels, and are synthesised into a new theoretical framework: the Multi-level Leisure Mechanisms Framework. This framework situates understanding of leisure activities within the theoretical lens of complex adaptive systems and aims to support the design of more theory-driven, cross- disciplinary studies.

## Introduction

Leisure (how a person spends their free time) has been described as “the principal driving force underpinning the human desire to render life meaningful … or to give it a sense of passion, pleasure and purpose”.^1^ Leisure activities are frequently defined as voluntary non-work activities that are engaged in for enjoyment,^2^ and encompass actions such as: taking part in hobbies; participating in arts; taking educational classes; reading; watching television; socialising; shopping; listening to music; volunteering; joining religious activities; participating in political parties, trade unions, or environmental groups; engaging in libraries, archives, culture, and heritage activities; taking part in sports or exercise groups; cooperating in community, neighbourhood, or tenants’ groups; and participating in social clubs.

Thousands of studies have shown a relationship between leisure engagement and both physical and mental health, including showing efficacy and causality through experimental and clinical intervention studies, and the longitudinal and beneficial nature of these effects, the depth of their impact on human wellbeing, and their potential to affect diverse populations through observational and qualitative studies.^3–13^ In relation to mental health, studies have suggested the value of leisure activities in the prevention and management of mental illnesses such as depression, anxiety, stress, bipolar disorder, and schizophrenia.^14–22^ In relation to physical health, there is evidence that engagement in leisure activities can lead to improvements in self- reported health^23–27^ and play a protective role against the development of conditions such as coronary heart disease,^28,29^ cognitive decline and dementia,^30–38^ and age- related physical decline including chronic pain, frailty, and disability.^39–44^ For individuals who already have a chronic illness, leisure can support the management of symptoms and help to reduce the rate of illness progression.^43,45,46^ Furthermore, there is even literature showing a relationship between leisure engagement and increased longevity.^29,47–53^

However, there is no unifying theoretical framework explaining how leisure activities affect health: what the mechanisms of action are by which engagement with leisure activities leads to the prevention, management, or treatment of mental and physical illness. Over the past two decades, many theories have been developed within various disciplines that could explain the effects of leisure on health, including those focusing on individual or intraindividual responses to leisure activities at a micro- level, those focused on how leisure activities interact with and affect wider social groups at a meso-level, and those focused on how these activities sit within and influence broader societal, cultural, and political contexts at a macro-level. Numerous studies have explored the relationship between different types of leisure activities and specific mechanisms, with a growing number of systematic reviews synthesising and critiquing the quality and findings of the literature on individual mechanisms.^1–3^

However, much of this discussion has occurred within individual disciplines and with a narrow lens onto specific mechanisms in isolation from others. Little attempt has been made to take a whole-system approach, map the key mechanisms across disciplines, consider how different mechanisms interact with one another, and develop a unifying framework. Such work is fundamental from a research perspective, to ensure that research does not become entrenched in disciplinary silos and that it remains theoretically pluralist and connected.

Understanding mechanisms is also pertinent from a practical perspective, given the roll-out internationally of schemes such as social prescribing that involve referring individuals to leisure and other community activities.^57^ To refer individuals to appropriate leisure activities with realistic potential for a positive effect on mental and physical health, as well as to design and test bespoke interventions for targeted patient groups, it is crucial that the mechanisms by which leisure activities affect health are understood. Indeed, there have been calls for logic models and theories of change for social prescribing programmes, but comprehensive and research-driven frameworks are still scarce. To address this need, in this Review we identify and catalogue potential mechanisms of action from diverse academic disciplines, linking leisure activities with health outcomes at a micro-level, meso-level, and macro-level. We do not attempt to catalogue or appraise every single study ever done on leisure mechanisms of action; such work would be beyond the scope of any individual review, and is the focus of the growing number of mechanism-specific systematic reviews mentioned above. Instead, the focus of this Review is to catalogue each specific potential mechanism, either empirically or theoretically discussed in previous research in relation to leisure activities, and to present these mechanisms in a new theoretical framework: the Multi-level Leisure Mechanisms Framework.

## Terminology

### Defining leisure

Leisure has proved a challenging concept to categorise, and no single model predominates. Leisure activities are generally considered as activities done in an individual’s free time (ie, when not working or otherwise employed). Early categorisations of leisure differentiated activities as relaxed, serious, and unclassified, with relaxed leisure including socialising, watching television, reading, and listening to music; serious leisure including sports, games, arts, and hobbies; and unclassified leisure including thinking, resting, and studying.^58^ Multiple other categorisations have since been proposed. Some of these have focused on how active leisure activities are, differentiating active activities (such as recreational sports) from passive activities (such as watching television or listening to music),^59^ or between high- demand leisure activities (eg, sports and gardening), low- demand leisure activities (eg, sewing and reading), and instrumental activities (eg, shopping).^23^ Others have focused on the goal of the activity, such as time-out leisure (including any solitary, passive activities) versus achievement leisure (including activities that provide challenges for individuals).^60,61^ Others still have focused on the expressive nature of leisure activities with further categories proposed, such as transitional or expressive leisure (eg, engaging in arts, hobbies, music, or sports) and fantasy or imagery (eg, reading or watching television).^62^ Notably, many categorisations have focused specifically on the social elements of leisure activities. Some have made social leisure activities merely another category of leisure,^23,63^ but others have drawn social leisure activities away from the umbrella term of leisure and instead focused on them as part of wider social engagement, which includes both social activities carried out for enjoyment and formal social activities carried out, for example, for work.^64–68^

Classification of leisure activities has generally aimed to identify substitutable activities, which either contain common elements (so that engaging in any activities within a particular category would produce a similar experience), or that are favoured by individuals with similar behaviours or preferences.^62^ This classification is particularly relevant to health research, if it is hypo- thesised that different categories of activities could have differential effects on health. However, such approaches have produced overlapping or conflicting models, with no real consensus on how activities cluster together. Further, leisure is defined and valued differently in different cultures,^69,70^ so it is conceptually challenging to differentiate whether specific leisure activities could have more health benefits than others when the membership of these categories is so flexible.

Therefore, in this Review, we define leisure activities as voluntary activities not related to employment responsibilities that are engaged in during free time, predominantly for enjoyment. However, we make no attempt to provide strict criteria for which activities are or are not eligible for inclusion under the umbrella of leisure, or to subgroup activities based on common elements or common features between those who engage in them. Instead, we propose that such activities can be grouped and considered to affect health based on the mechanisms of action that they stimulate.

### Defining mechanisms of action

Leisure activities are complex interventions, in that they contain several interacting components that can lead to various outcomes at both individual and group levels.^71^ As a result, researching their effects involves understanding their so-called active ingredients (the specific components involved in an activity or intervention), what causal processes these ingredients then set in motion (the mechanisms by which they affect outcomes), and what factors act as moderators (leading to variations in these causal processes). Specifically, this Review focuses on the second of these aspects: how leisure activities lead to mental and physical health outcomes, hereafter referred to as the mechanisms of action. Understanding mechanisms of action means understanding the theo- retical basis linking leisure to health, which is key to research on leisure and health. As the UK Medical Research Council’s guidance on complex interventions states, “a good theoretical understanding is needed of how the intervention causes change”, so researchers are recommended to “develop a theoretical understanding of the likely process of change by drawing on existing evidence and theory”.^71^

This emphasis on theory-driven approaches in complex intervention research has led to mixed responses. Although there are many examples of it strengthening the design and evaluation of interventions, there have been concerns that the emphasis on theory-based approaches can lead to researchers adopting so-called off- the-shelf theories without considering, in depth, their applicability to the intervention being studied.^72^ It has been argued that the application of simple or widely used theoretical frameworks can lead to a narrow lens for studies, excluding important mechanisms that lie outside a given framework.^72^ This is a criticism that could be directed at studies of various types of leisure activities, for which, despite the involvement of researchers with diverse expertise, disciplinary silos have often led to narrow considerations of relevant theory.^73^ Consequently, there has been a call for health researchers to move towards incorporating a broader range of potential theoretical perspectives within research on complex interventions, which cross disciplines and move beyond focusing on individual specific theories to explain complex effects.^72^ Although some disciplines, such as behaviour change research, have been developing taxonomies of theories to support more sophisticated theory-driven research,^74^ such an approach is still rare and has not been applied to research on leisure.

Therefore, we review potential mechanisms of action to improve understanding of the effects of leisure activities on mental and physical health, and to support the design, development, implementation, and replication of bespoke leisure programmes targeting specific health outcomes in different populations. As the focus of this Review is on potential mechanisms of action that could explain the relationship between leisure and health, we include mechanisms that have been either empirically tested or theoretically discussed in relation to leisure in previous research.

## Reviewing mechanisms of action

Over 600 potential mechanisms of action by which leisure activities might affect health have been identified (appendix). Some of these mechanisms represent immediate responses to leisure activities, whereas others are proposed to emerge over time or through changes in wider aspects of human development and behaviour. Some mechanisms operate at micro-levels, affecting individuals or very small groups, whereas others operate at meso-levels, affecting larger groups, communities, and institutions, or at macro-levels, affecting societies and cultures at large. Each mechanism is presented here in terms of the direction of effect that is most likely to promote health, but we discuss the potential for reverse effects in more detail later in the text. A summary of all mechanisms is provided in the text below, an over- view of overarching categories and subcategories of mechanisms is provided in the panel, and full lists of all identified mechanisms are provided in the appendix pp 3−14. The appendix also includes definitions and key references for each mechanism identified (appendix pp 15−38), and provides citations of literature linking each group of mechanisms to engagement in leisure, other mechanisms, and health behaviours and outcomes (appendix pp 39−43).

### Psychological processes

At an individual level, leisure activities can have immediate effects on affective states, build resilience, develop a sense of self, support individual personal transformation, help individuals to flourish, build psychological capabilities, and build psychological resources. At a group level, leisure activities have the potential to build group mind, change group attitudes, and lead to changes in language (appendix pp 15−20).

### Biological processes

Biologically, at an individual level, leisure activities can activate mechanisms within the endocrine, immune, and central nervous systems, affect the cardiometabolic system, affect physical performance, and elicit multi- system biological responses. At a group level, leisure activities (especially those that involve engagement with the natural environment) have the potential to affect group-level biological factors relating to environmental diversity, and mechanisms relating to the susceptibility of groups to disease (appendix pp 21−26).

### Social processes

At an individual level, leisure activities can activate mechanisms relating to social activity and social rela- tionships, support specific learning and traits, and build social resources. At a group level, leisure engagement also has the potential to build group strength and affect group power (appendix pp 27−30).

### Behavioural processes

At an individual level, leisure engagement can lead to changes in behavioural mechanisms such as those relating to the development of habits, affect behavioural decisions, affect behavioural drive, affect behavioural development, and affect personal location. At a group level, leisure activities have the potential to enhance behavioural processes relating to cooperation and approaches to health, and to affect the availability of assets (appendix pp 35−35).

### Health behaviours

Engagement with leisure can lead to individual engage- ment in healthy behaviours, and to reduced engagement in unhealthy behaviours. At a group level, leisure engagement also has the potential to affect health care delivery and performance (appendix pp 36−38).

## Developing a new theoretical framework

There are, clearly, many potential mechanisms of action by which leisure activities might affect health, both at individual and group levels. However, although the lists and tables provided show each mechanism as an entity in its own right, the literature in fact highlights that these mechanisms not only affect health behaviours and physical and mental health outcomes directly, but also interact with one another both across levels (eg, micro- level mechanisms bidirectionally interacting with macro-level mechanisms) and between domains (eg, psychological mechanisms bidirectionally interacting with social mechanisms) (appendix pp 39-44 provides a summary of such literature). As such, although some of these mechanisms can be directly activated via leisure engagement and have an immediate effect on health, others might be part of more complex indirect pathways that have effects on health over longer periods of time. Therefore, it is evident that any framework bringing together these mechanisms needs to take these inter- actions into account. As such, the Multi-level Leisure Mechanisms Framework proposes that all mechanisms exist symbiotically, interacting across levels and domains (figure). To support visualisation, this framework continues the loose categorisation of the mechanisms as psychological, biological, social, and behavioural processes, but many of the mechanisms included could be considered to fall under multiple headings and transcend categorisation. We previously highlighted that leisure activities are complex interventions, and understanding this com- plexity is key to advancing theoretical conceptualization of how these mechanisms work together. Therefore, we outline below some of the fundamental theoretical principles drawn from complexity science (including programme theory, ecological theory, and systems theory), and show how they apply to our new Multi-level Leisure Mechanisms Framework.

First, leisure activities involve multiple components and simultaneous causal strands.^75^ As such, no leisure activity will activate just one causal mechanism, and applying simple models of mechanisms to complex leisure activities risks overstating the causal contribution of individual mechanisms.^75^ Indeed, many of the implications of the identified mechanisms will be realised not only as a result of the mechanisms themselves, but also as a result of the interaction of multiple different mechanisms.^76,77^ This interaction of mechanisms can lead to new hybrid mechanisms emerging (adaption).^76^ Consequently, the health effect of leisure activities cannot be understood as a sum of the individual parts,^77^ and attempting to break down a complex system such as leisure engagement into individual elements (eg, research attempting to test specific mechanisms artificially isolated from other mechanisms) risks altering the processes that we seek to understand.^78^

Second, the mechanisms involved in complex inter- ventions are non-linear and can involve positive and negative feedback loops, recursive causality (whereby mechanisms can reinforce one another via feedback loops, leading to outputs functioning as inputs), self-reinforcement (whereby the successful activation of one mechanism might lead to adaptation of an individual’s engagement with a leisure activity so that this mechanism is further enhanced), disproportionate relationships (whereby small changes in individual’s leisure patterns can lead to big differences in mechanisms and outcomes), and emergent outcomes (whereby mechanisms and outcomes develop during the implementation of a leisure intervention).^75,77^ Indeed, mechanisms can even be autocatalytic, in that experiencing a mechanism might recatalyse the original process of engagement, providing a virtuous cycle of activity and effect.^78^ The model of the Multi-level Leisure Mechanisms Framework presented here attempts to highlight this non-linearity and complexity within the confines of presenting a com- prehensible diagram, but leisure activities must not be mistakenly considered as deterministic systems.^79^

Third, we cannot view leisure activities as discrete packages of components that exist in isolation from their contexts.^72^ Instead, we need to recognise that mechanisms will only occur (and make sense) when considering the dynamic interactions between leisure activities and micro- level, meso-level, and macro-level contextual factors.^77^ Indeed, complex systems are considered to be radically open, meaning that it can be almost impossible to discern where the boundaries of specific components of the intervention and the broader environment lie.^78^ Health (and mechanisms that affect health) is determined at multiple levels, and is affected by historical, political, economic, temporal, and spatial factors.^80^ When specific leisure activities are engaged in as part of a larger complex system (such as a social prescribing scheme involving clinician referral to leisure activities that therefore situates leisure engagement as part of the complex system of a community, and as part of health-care delivery), there might be aggregate complexity at play that can lead to the introduction of yet further mechanisms.^79^ Moreover, the mechanisms presented here operate not only within a single, albeit complex, cross-sectional state, but also in conjunction with historical events, both those experienced by individuals themselves and those occurring within society.^76^ So the investigative focus of mechanisms of action relating to leisure engagement needs to be on dynamic systems, rather than on artificially static states.^78^ Therefore, although the framework presented here is described in isolation, it is intended to be used and interpreted in light of historical and present contextual factors that moderate engagement with leisure and how leisure activities affect health.

Fourth, complex systems do not exist in equilibrium and are not static.^76^ Rather, they constantly adapt to feedback from interactions between elements within the system, and between elements and their environment.^77^ Control of the mechanisms involved is not hierarchical but is distributed across different parts of the system, such that both the leisure activities themselves and other elements of the system are all capable of affecting which mechanisms are activated.^79^ Therefore, leisure activities that involve the activation of specific mechanisms at a single point in time for a specific group of individuals will not necessarily always continue to involve these mechanisms.^79^ Changes will undoubtedly occur as elements of the system co-evolve. Therefore, although this framework is intended to help to elucidate how and why leisure activities affect health, specific mechanisms identified as being involved in particular activities cannot be taken as constants and should be expected to change over time.

Finally, although this framework brings together a large number of potential mechanisms of action, it is not, and could never be, complete.^77^ There might be mechanisms of action that have not yet been identified, or that might not yet exist but could still emerge as patterns of leisure activities and behaviours evolve. Further, many of the mechanisms activated by leisure engagement will not be unique to leisure, but will also be activated by non-leisure activities in everyday life. Consequently, as each mechanism of action itself is also the individual subject of ongoing research, both in relation to leisure activities and to broader activities, our conceptual understanding of these mechanisms is constantly evolving.

## Discussion

The Multi-level Leisure Mechanisms Framework is intended to support the design of future research into the effect of leisure engagement, by highlighting the potential mechanisms that could link leisure engage- ment to health outcomes. But it also highlights several challenges facing such research. First, this Review focused on potential mechanisms of action. Some of the mechanisms included have been tested comprehensively across multiple different leisure activities, some have been tested only in relation to specific leisure activities, and others remain hypothetical but not tested (appendix pp 39−44). We encourage more systematic reviews that explore specific mechanisms or groups of mechanisms in more detail, identifying and assessing what research has been done, and new studies into mechanisms that have been the focus of theoretical, but not empirical, research, to clarify which mechanisms are actual rather than potential. In doing so, it will be important to consider carefully whether a specific intervention is likely to be disruptive enough to bring about desired change, especially for mechanisms that represent deep-seated patterns of traits or behaviours.^81^ As many of these mechanisms represent complex structures of their own, the choice of measurement approaches for different mechanisms (such as the use of validated scales or the choice of biological markers) and the study design (especially the longitudinal tracking of any changes over varying periods of time) are going to be crucial to ensure that such studies are conceptually appropriate and adequately capture the mechanism that they are trying to measure.^78^ In particular, we feel it is important that the research starts to move beyond just focusing on those mechanisms and measures that are frequently incorporated, and into researching those that are less well understood.^76^

Second, it is important to remember the lens of complex adaptive systems. Studies that focus too narrowly on a single mechanism, attempting to manipulate it in isolation from its context, are unlikely to produce reliable data.^75^ We need more research assessing the contributing strength of mechanisms to specific outcomes, and testing whether changes in aspects of an intervention or changes in some mechanisms can improve the workings of other mechanisms. But we should remember the principles of recursive causality, disproportionate relationships, and non-linearity (among others discussed in this Review).^75,77^ Therefore, the framework and mechanisms that we present are intended to support the development of more multi-theory, cross-disciplinary research that embodies theoretical and methodological pluralism. We particularly encourage studies that take an outcome-wide approach in researching effects of leisure on multiple mechanisms simultaneously.^82^

It is also important to remember that complex inter- ventions do not have the same consistent pattern of effect as simple interventions, and differences in findings when replicating studies do not necessarily mean unreliable data.^75,77^ A wide range of factors can determine which mechanisms are activated for different individuals, and can moderate any relationship between these mechanisms and health outcomes, as well as affecting individuals’ leisure engagement in the first place (appendix p 44). These can operate at micro-levels, with an individual’s leisure engagement and its effects on their health influenced by individual physical, psy- chological, and social traits and how these are embodied, and also at meso-levels and macro-levels by how that individual is positioned spatially and culturally (emplacement).^80^ These factors are not static, but evolve across the life course and interact with broader life events. Although there are particular periods that have been identified as crucial or sensitive developmental periods, it is more broadly recognised that the timing and sequence of activities, such as leisure activities, can lead to different responses.^83^ As a result, the period and length of leisure engagement can affect the way leisure embeds itself into the structure and functioning of individual biological and behavioural systems.^84^ The existence of so many factors that predict leisure engagement and moderate the way leisure engagement affects health could cause concerns among researchers keen to standardise interventions and replicate precise research findings. However, it is increasingly recognised that complex interventions often give better results when tailored to local circumstances, and do not necessarily perform in the same way for different individuals.^71^ For mechanisms that are central to a specific intervention, these local adaptations or variations between individuals might not alter results much. But for subsidiary mechanisms, local adaptations or differences between individuals could lead to different mechanisms being engaged. We need to recognise that this difference is to be expected and might represent a natural adaptation of an activity to its setting or recipient. As a result, our research approach needs to be suitably flexible to capture elements of unpredictability and innovation.^81^

Several research questions remain to be explored. We have little information on the effect of moderating factors on the workings of individual mechanisms, so future studies could consider whether some mechanisms work only in the presence of particular contextual factors. The framework could also enable the design and execution of statistical modelling, predicting and then testing specific hypotheses using datasets. Given that many of the mechanisms presented here are also activated by non- leisure activities, it will be important to assess how leisure activities compare to non-leisure activities, either in the degree to which they are able to activate specific mechanisms or in the number of mechanisms that they manage to activate simultaneously. Additionally, further work is needed to explore what the actual components (active ingredients) of specific leisure activities are, as has been done for other areas of research, such as behaviour change interventions.^85–87^ This research would enable the development of clearer logic models con- necting interventions involving specific components with particular patterns of mechanisms of actions and specific health outcomes, and would therefore advance understanding of the health effect of leisure engagement.

### Limitations

First, although every attempt was made to ensure a cross- disciplinary approach in identifying and cataloguing mechanisms and to refer to the names of other mech- anisms where similar concepts existed in different fields, differences in terminology between disciplines might have led to the omission of some mechanisms. It is also to be noted that each of the described mechanisms involves complex underlying processes. For example, any psychological or biological mechanism identified occurs as a result of many biological micro-processes, such as the firing of synapses. We do not focus on these underlying micro-processes, but concentrate instead on the broader processes that can occur as a result of leisure engagement and that can provide plausible explanations for the health outcomes reported in research. Further, each of the mechanisms listed here has been the subject of (often extensive) previous research, and many of these mechanisms remain the focus of ongoing studies. Therefore, it is expected that definitions and conceptualisations will change over time. We present this Review not only as a comprehensive and up-to-date synthesis on mechanisms to date, but also as a dynamic and changing catalogue and framework.

Second, we focused specifically on the mechanisms by which leisure engagement can have a positive effect on health. As such, each mechanism is presented in terms of the direction of effect that is most likely to promote health. Not every mechanism is capable of being activated by every type of leisure activity. For example, mechanisms relating to engagement with green spaces (such as increased levels of vitamin D or improved gut microbiota) will be activated only if leisure engagement somehow brings individuals into contact with those green spaces. Additionally, some leisure activities can, by virtue of the active ingredients they contain, lead to more health benefits than others. Moreover, because this Review focused on potential mechanisms (ie, those that have been either dem- onstrated empirically or discussed theoretically), leisure activities might not be capable of causally affecting every mechanism listed, and the inclusion of a mechanism in this Review does not imply that every leisure activity has the potential to activate it. We have highlighted in the appendix (pp 34−44) where evidence is strongest and weakest and encourage future intervention studies and systematic reviews. Addi- tionally, several studies have identified the negative effects that leisure can have in some situations, from interaction with nature leading to exposure to vector- borne diseases, allergenic pollen, and hazardous materials such as toxic pollutants,^88^ to arts engagement leading to adverse health effects (such as hearing loss as a result of loud music)^89^ and negative social control (eg, as a propaganda tool),^90^ to screen-based activities such as television viewing being associated with negative outcomes such as cognitive decline.^91^ Indeed, literature on deviant leisure has identified specific activities that are routinely associated with adverse outcomes.^92^ Therefore, we do not propose that all leisure is a panacea. Some mechanisms can also be positive in moderate amounts, but become maladaptive if taken to extremes, and engagement with leisure itself in excess can become a maladaptive obsession.^93^ As such, although the Multi-level Leisure Mechanisms Framework presents the likely direction of effect, researchers are encouraged to maintain an open mind when researching these mechanisms and to predict and test rather than assume the response.

Third, this Review focuses on the mechanisms of action by which mental and physical health outcomes are achieved. In the Introduction, we referred to mental and physical health outcomes as including the incidence of, management of, or recovery from mental or physical illness and the promotion of wellbeing. However, there are many other aspects of health that can be considered outcomes, so the boundary between mechanisms and outcomes is naturally blurred. As a result, some of the mechanisms identified are also outcomes relating to mental or physical health them- selves. For example, improvements in purpose, quality of life, and affect are often considered aspects of mental health and are the primary outcome for many studies, whereas measures of gait, balance, and physical function are often considered core physical health outcomes. Therefore, any proposed logic model for an intervention focused on achieving these outcomes would require a rearrangement of the model to move these mechanisms to outcomes. This observation highlights the need to interpret the terms used in this Review with appropriate flexibility.

We have identified and catalogued a large number of theories, which could give the impression that leisure activities can trigger every possible mechanism, leading to the risk that the framework presented here becomes a totalising theory that attempts to explain everything and therefore explains nothing.^80^ Many further mechanisms were considered in relation to this framework but were not included, as there was neither existing empirical evidence nor a clear theoretical rationale for how leisure could trigger them. Some of the included mechanisms, such as those relating to the generational transmission of biological processes, have been hypothesised to relate to specific leisure activities, but can take generations to occur, and the evidence specifically relating these to leisure is weak (appendix p 41). We include these mechanisms as they meet our criteria for being potential mechanisms, and their inclusion increases the explan- atory power in understanding leisure. However, we encourage readers to consider that some mechanisms are probably not easily activated, and might only emerge in a gradual and small way over time, so they should not necessarily be the primary goal for leisure activities. Overall, this Review might have provided a framework that is complex to understand,^94^ but this is only a reflection of the complexity of the processes involved.

Finally, under the conceptual understanding that leisure activities are complex adaptive systems, it is impossible to understand and predict precisely how they work. Nevertheless, an enhanced understanding and stronger overarching theoretical framework is crucial to advancing research on this subject.

## Conclusions

We have identified over 600 mechanisms of action by which leisure engagement can affect health and health behaviours, through networks of psychological, bio- logical, social, and behavioural processes, at micro- levels, meso-levels, and macro-levels, and have synthesised these findings into a new Multi-level Leisure Mechanisms Framework. No system will ever be able to include every single mechanism and this framework does not aim or claim to do so. However, this is the most rigorous review of potential mechanisms of action for leisure engagement done so far. We anticipate that the framework will need to evolve as the theories themselves develop, and as new research is done and new mechanisms are described. Areas where there is already suggestion of large developments to come in the next few years include research on genetics and epigenetics, behaviour change, and environmental factors. Nevertheless, it is hoped that this Review and framework will support the design of more theory- driven, cross-disciplinary studies that explicitly consider the mechanisms underlying the effects of leisure engagement on mental and physical health.

### Search strategy and selection criteria

First, we developed a list of disciplines considered likely to have examined mechanisms relating to leisure activities and health (appendix p 1). Three key textbooks for each discipline were selected through personal experience and consultation with experts in each field. Textbook mentions of a mechanism that was either (1) theoretically discussed as a mechanism of action linking one or more types of leisure activity with health, or (2) empirically tested as a mechanism were added to a master database of mechanisms. Second, we searched for key papers using a list of key terms (appendix) in the following databases: Google Scholar, Scopus, Web of Science, PubMed, ScienceDirect, Europe PMC, and PsycINFO. Search criteria included articles published in English between Jan 1, 1950, and Jan 31, 2020. Relevant papers were then manually searched, and any further mechanisms identified were added to the database. Third, we consulted experts in each field from the MARCH Mental Health Research Network. After the mechanisms had all been catalogued in the master database, we went through each individually and only included in the final review those that were deemed relevant to the research question, definable and distinct from other mechanisms, and either theoretically proposed or empirically shown to act as a mechanism of action linking one or more leisure activities with health (ie, a potential mechanism of action, as described in the review).

A comprehensive description of the search strategy and selection criteria is provided in the appendix pp 1−2.

## Supplementary Material

Appendix
