# Supplementary material for: How leisure activities affect health: a narrative review and multi-level theoretical framework of mechanisms of action
Source: Lancet Psychiatry. Author manuscript; Available in PMC 2022 Jul 23. (PMC7613155; doi:10.1016/S2215-0366(20)30384-9)
Supplement: Appendix [file EMS150895-supplement-Appendix.docx]

**How do leisure activities affect health?**

Supplementary Materials

**Table of Contents**

[Search Strategy & Selection Criteria 1](#_Toc60908689)

**Overview of mechanisms**

[1. Full List of Mechanisms 3](#_Toc60908690)

[1.1 Psychological processes 3](#_Toc60908691)

[1.2 Biological processes 6](#_Toc60908692)

[1.3 Social processes 9](#_Toc60908693)

[1.4 Behavioural processes 11](#_Toc60908694)

[1.5 Health behaviours 14](#_Toc60908695)

**Mechanisms in detail**

[2. Psychological Processes: in detail 15](#_Toc60908696)

[2.1 Micro-level 15](#_Toc60908697)

[2.2 Meso-/Macro-level 19](#_Toc60908698)

[3. Biological Processes: in detail 21](#_Toc60908699)

[3.1 Micro-level 21](#_Toc60908700)

[3.2 Meso-/macro-level 26](#_Toc60908701)

[4. Social Processes: in detail 27](#_Toc60908702)

[4.1 Micro-level 27](#_Toc60908703)

[4.2 Meso-/Macro-level 29](#_Toc60908704)

[5. Behavioural Processes: in detail 31](#_Toc60908705)

[5.1 Micro-level 31](#_Toc60908706)

[5.2 Meso-/Macro-level 34](#_Toc60908707)

[6. Health Behaviours: in detail 36](#_Toc60908708)

[6.1 Micro-level 36](#_Toc60908709)

[6.2 Meso-/macro-level 37](#_Toc60908710)

**Further tables**

[7. Literature linking leisure, mechanisms, and health 39](#_Toc60908711)

[8. Factors that can predict leisure engagement or moderate the mechanisms linking leisure activities with health 44](#_Toc60908712)

[References 45](#_Toc60908713)

*How leisure activities affect health: a narrative review and multi-level theoretical framework of mechanisms of action*

*Daisy Fancourt, Henry Aughterson, Saoirse Finn, Emma Walker, Andrew Steptoe*

*Lancet Psychiatry (2021)*

# **Search Strategy & Selection Criteria**

This review had the aim of identifying potential mechanisms of action linking leisure engagement with health outcomes, taking a cross-disciplinary perspective. A key challenge, however, is that the language used across disciplines, especially for ‘mechanisms of action’, varies substantially. Therefore, we adopted a three-point strategy for identifying mechanisms as part of a larger five-stage process.

1. **Key text searches**

First, four authors (DF, HA, SF & EW) each independently developed a list of disciplines that were considered likely to have examined mechanisms relating to one or more leisure activities and health. The lists were then combined and duplicates excluded to provide a full list, which included the following:

*aesthetics, affective psychology, architecture, arts in health, behavioural economics, behavioural science, bioacoustics, biological anthropology, clinical psychology, cognitive psychology, community psychology, computational psychiatry, cultural anthropology, cultural psychology, cultural studies, ecological psychology, ecology, economics, education, engineering, evolutionary psychology, genetics, health economics, health geography, health humanities, health promotion, heath psychology, health sociology, leisure studies, medical humanities, medicine, music psychology, nature studies, neuroscience, occupational therapy, performance science, philosophy, positive psychology, psychiatry, psychobiology, psychophysics, psychotherapy, public health, recreational therapy, social epidemiology, social geography, social psychology, social work, sociology, sport psychology*

Four authors (DF, HA, SF & EW) then identified 3 key textbooks for each discipline that were either general textbooks on the discipline (where the discipline as a whole was felt to be of relevance to understanding leisure and health), or textbooks that focused on the specific cross-over between a discipline and leisure and health. These textbooks were identified through personal experience in respective fields of expertise and consultation with experts in each field from the MARCH Mental Health Research Network (see below). The four researchers then pooled their suggestions for key texts and discussed until a consensus was reached on which texts were most appropriate.

These four authors then split the reading of the textbooks between them and whenever mention was made of a mechanism that was either (i) theoretically discussed as a mechanism of action linking one or more types of leisure activities with health, or (ii) empirically tested as a mechanism, it was added to a master database of mechanisms.

1. **Database searches**

Second, we conducted database searches for key papers focusing on leisure and mechanisms of action using the following key terms:

| *Leisure* | *Mechanisms* | *Outcome* |
| --- | --- | --- |
| *Activity(ies)* | *Mechanism(s) of action* | *Health* |
| *Art(s)* | *Mechanism* | *Mental health* |
| *Club(s)* | *Process* | *Physical health* |
| *(Community) group(s)* | *Theory* | *Health behaviour(s)* |
| *Craft(s)* | *Pathway* | *Wellbeing* |
| *Creative(ity)* | *Causal* |  |
| *Culture* | *Affect* |  |
| *Dance* | *Effect* |  |
| *DIY* | *Impact* |  |
| *Exercise* | *Psychological* |  |
| *Free time* | *Biological* |  |
| *Game(s)* | *Social* |  |
| *Green space(s)* | *Behavioural* |  |
| *Heritage* |  |  |
| *Hobby(ies)* |  |  |
| *Leisure* |  |  |
| *Libraries* |  |  |
| *Music* |  |  |
| *Nature* |  |  |
| *Outdoor(s)* |  |  |
| *Reading* |  |  |
| *Religion* |  |  |
| *Social engagement* |  |  |
| *Socialising* |  |  |
| *Sport(s)* |  |  |
| *Television* |  |  |
| *Volunteer(ing)* |  |  |

Keyword searches were conducted using the following databases: Google Scholar, Scopus, Web of Science, PubMed, ScienceDirect, Europe PMC, and PsycINFO. We only looked at studies that had been published in English since 1^st^ January 1950 up until 31^st^ January 2020. Any relevant papers were then manually searched and any further mechanisms identified were added to the database.

1. **Expert opinion**

Third, the MARCH Mental Health Network Disciplinary Expert Group was consulted. The MARCH Mental Health Network is one of the UK’s national mental health research networks funded by UK Research and Innovation. The network focuses on the role of social, cultural and community engagement on mental health (including all leisure activities included within this review) and consists of over 1,300 member researchers and community organisations. Of these, 42 members are part of the Disciplinary Expert Group, with each specialising in a different field related to social, cultural and/or community engagement and mental health.

The Disciplinary Expert Group members were all contacted and asked to provide details on further key texts either reviewing mechanisms or focusing on specific mechanisms of action. The additional texts were then screened and further mechanisms identified were also added to the database. As explained above, the Disciplinary Expert Group were also called upon throughout the process to clarify terminology, identify key texts, and supporting the identification and definition of mechanisms in the review.

1. **Mechanism screening**

Once the mechanisms had all been catalogued in the master database, four researchers (DF, HA, SF & EW) went through each mechanism individually. For a mechanism to be included in the final review, it had to be deemed (a) relevant to the research question, (b) definable and distinct from other mechanisms, and (c) either theoretically proposed or empirically shown to act as a mechanism of action linking one or more leisure activities with health (i.e. a “potential mechanism of action”, as described in the review). Where there was disagreement on any of these three criteria, the researchers either returned to literature on that mechanism or sought expert advice from the Disciplinary Expert Group until consensus was reached.

1. **Thematic grouping**

All included mechanisms were then grouped into themes with similar mechanisms and approved by the four researchers (DF, HA, SF & EW). The fifth author (AS) then undertook a full review of all included mechanisms and their thematic grouping and all researchers approved the final versions of the tables.

# **Full List of Mechanisms**

## **1.1 Psychological processes**

| Categories | Sub-categories | Mechanisms |
| --- | --- | --- |
| MICRO-LEVEL | | |
| AFFECTIVE STATES | Eliciting affective responses | Increased experience of positive emotions  Decreased experience of negative emotions  Decreased stress  Increased eustress  Changes in valence  Experience of pleasure |
|  | Supporting emotion regulation | Improved attention-focused emotion regulation  Improved knowledge-focused emotion regulation  Improved expression-focused emotion regulation  Increased self-soothing  Changes in mood  Broadening of momentary thought-action repertoire |
| RESILIENCE | Supporting coping | Increased emotion-focused coping  Increased problem-focused coping  Increased stress buffering  Decreased catastrophizing  Reduced suicidal ideation  Increased terror management  Improved management of grief  Development of supportive internal dialogue  Increased adjustment  Increased accommodation  Greater availability of cognitive bandwidth |
|  | Building psychological strength | Increased self-confidence  Increased resilience  Increased vitality  Improved psychic homeostasis  Increased sense of continuity  Improved tolerance of uncertainty  Decreased submissiveness  Decreased denial  Decreased resignation  Reduced subjective weathering  Increased conservation of resources |
| SENSE OF SELF | Building identity | Formation & affirmation of self-identity  Improved self-knowledge  Improved self-concept  Decreased self-discrepancy  Decreased de-individuation  Increased self-expression  Development of a future self  Increased self-awareness |
|  | Developing self-acceptance | Increased self-esteem  Increased self-enhancement  Increased self-protection  Reduced self-stigmatisation  Increased self-affirmation  Decreased self-derogation  Increased authenticity  Acceptance of personal fallibility |
| PERSONAL TRANSFORMATION | Supporting changing identity | Reduced ‘loss of self’  Reduced biographical disruption  Increased reminiscence  Increased self-restoration  Provision of alternative identities  Building of new narratives  Development of personal pathography |
|  | Aiding personal evolution | Increased openness to experience  Increased agreeableness  Increased conscientiousness  Increased extroversion  Decreased neuroticism  Increased optimism  Personal transformation  Increased growth  Increased regulatory focus  Self-transcendence |
| FLOURISHING | Enhancing meaning in life | Provision of meaning  Increased sense of life being worthwhile  Improved life satisfaction  Increased sense of coherence  Formation and affirmation of central values  Increased gratitude  Increased purpose |
|  | Developing autonomy | Increased self-locus of control  Changes in self-attribution  Increased autonomy  Increased agency  Increased empowerment  Compensation for imbalanced demand-control in other life domains |
| PSYCHOLOGICAL CAPABILITIES | Supporting cognitive learning | Enhanced information processing  Enhanced semantic memory  Enhanced episodic memory  Enhanced procedural memory  Enhanced convergent thinking  Enhanced divergent thinking  Improved reasoning  Expansion of perceptual sets  Increased use of heuristics  Enhanced transfer effects  Enhanced aesthetic judgement  Enhanced attention |
|  | Supporting emotional learning | Increased emotion recognition  Improved theory of mind  Increased empathy  Increased compassion  Enhanced sense of conscience  Increased decentering  Increased mentalising  Increased mind-mindedness |
|  | Supporting overarching cognitive processes | Enhanced processing fluency  Enhanced cognitive development  Enhanced cognitive restructuring  Enhanced transformative learning  Increased diversification of intelligence  Increased curiosity  Enhanced creativity  Enhanced visualisation  Increased imagination  Reduced cognitive decline |
| PSYCHOLOGICAL RESOURCES | Building general resources | Enhanced maturity  Increased human capital  Increased cultural capital  Improved educational attainment  Increased personal conversion factors |
|  | Building health-related resources | Expanded lay theories  Changes in illness cognitions  Increased health consciousness  Increased health literacy  Increased understanding of others’ health conditions  Increased salience of healthcare engagement  Experience of healing  Improved quality of life |
| MESO/MACRO-LEVEL | | |
| GROUP MIND | Developing a group self | Increased sense of group self  Increased collective rationality  Altered cultural perception  Increased group knowing  Enhanced sense-making |
|  | Developing group values and understanding | Increased meaning-making  Development of social norms  Reduced anomie  Increased collective responsibility  Development of collective consciousness |
| GROUP ATTITUDES | Reducing stigma | Reduced social labelling  Reduced prejudice  Increased tolerance  Disruption of stereotypes  Decreased social death |
|  | Changing attitudes to health | Changes in the social construction of illness  Decreased medicalisation of social deviance  Decreased cultural camouflage  Decreased risk discourse  Enhanced satisfaction with healthcare |
| LANGUAGE | Changing communication | Increased voice for minority groups  Enhanced group matrix  Increased spontaneous communication  Increased symbolic communication  Development of memes  Increased symbolic interactionism |
|  | Modifying group emotions | Elicitation of collective emotions  Increased collective emotional convergence  Decreased collective stress  Formation of collective memories  Development of collective effervescence |

## **1.2 Biological processes**

| Categories | Sub-categories | Mechanisms |
| --- | --- | --- |
| MICRO-LEVEL | | |
| NERVOUS SYSTEM | Activating perceptual processes | Changes in activation of the auditory system  Changes in activation of the visual system  Changes in activation of the somatosensory system  Changes in activation of the gustatory system  Changes in activation of the olfactory system  Changes in activation of the vestibular system  Increased proprioception  Increased interoception  Increased kinaesthesia  Increased perceptual sensitivity  Increased sensory equilibrium  Modulated levels of sensory deprivation  Increased multisensory integration  Decreased central & peripheral sensitisation  Reduced pain |
|  | Increasing brain activation | Increased overall cerebral activation  Improved overall cerebral blood flow  Increased activation of brain regions involved in basic vision and object perception  Increased activation of brain regions involved in attention and sensory processing  Increased activation of brain regions involved in knowledge and meaning  Increased activation of brain regions involved in reward, valuation or response to perceived beauty  Increased activation of brain regions involved in affective responses  Increased activation of the motor cortex  Activation of mirror neurons |
|  | Altering brain physiology | Changes in brain structure  Increased grey matter & grey matter integrity  Increased white matter & white matter integrity  Increased neurogenesis  Increased neuroplasticity  Decreased neurotoxicity  Reduced brain atrophy  Increased cognitive reserve |
|  | Modulating brain biomarkers | Changes in acetylcholine production  Increased dopamine production  Increased serotonin levels  Changes in oxytocin and vasopressin levels  Increased levels of opioids  Increased levels of cannabinoids  Increased levels of melatonin  Changes in GABA and glutamate levels  Decreased neuro-inflammation  Reduced disruption of biological rhythms |
|  | Modifying arousal | Changes in arousal levels  Increased chills  Changes in electrodermal activity  Changes in activation of the autonomic nervous systems  Improved autonomic tone  Changes in brainwaves  Brainwave entrainment  Decreased muscle tension  Increased nerve stimulation |
| ENDOCRINE & IMMUNE SYSTEMS | Changing hormone levels | Improved regulation of the Hypothalamic-Pituitary-Adrenal axis  Reductions in excessive glucocorticoid activity  Reductions in excess production of catecholamines  Changes in thyroid function  Changes in growth hormone levels  Changes in levels of sex hormones |
|  | Altering immune function | Changes in levels of innate immunity  Changes in levels of acquired immunity  Decreased cellular ageing  Decreased bacterial and viral infections  Changes in antibody levels  Changes in levels of growth factors  Decreases in levels of inflammation  Shifts in cytokine profiles  Decreased oxidative stress  Increased vitamin D levels |
| CARDIOMETABOLIC SYSTEM | Modulating cardiovascular factors | Reduced heart rate  Increased heart rate variability  Reduced vasoconstriction  Decreased blood pressure  Reduced hypertension  Changes in cardiovascular reactivity  Increased angiogenesis  Decreased aortic stiffness  Improved cardiac function |
|  | Modulating metabolic factors | Decreased glucose levels  Changes in lipid levels  Changes in haemoglobin levels  Decreased body mass  Changes in body composition  Improved gut microbiota  Changes to the microbiome |
| PERFORMANCE | Improving physical function | Improved balance  Improved gait  Improved reflexes  Decreased reaction time  Improved flexibility  Improved posture  Increased bone health  Improved limb function  Improved motor coordination  Increased ability to perform activities of daily living  Increased physical fitness  Reduced falls |
|  | Improving respiratory function | Strengthened respiratory muscles  Increased lung capacity  Improved structured respiratory variability  Improved diffusing capacity  Increased oxygen saturation |
|  | Improving voice | Improved speech  Improved language  Improved voice quality |
| MULTI-SYSTEM | Reducing load | Increased homeostasis  Changes in visceral factors  Reduced fatigue  Reduced allostatic load  Rebalanced stress reactivity  Reduced weathering  Reduced biological embedding  Decreased frailty |
|  | Increasing beneficial nature exposure | Enhanced exposure to microbial diversity  Enhanced exposure to phytoncides  Increased exposure to negative air ions  Reduced development of allergies and asthma |
|  | Affecting genetic / epigenetic factors | Reduced damage to telomeres  Changes in DNA methylation patterns  Changes in gene expression |
| MESO/MACRO-LEVEL | | |
| ENVIRONMENTAL DIVERSITY | Increasing positive environmental exposures | Increased nature connection  Increased biodiversity of spaces  Increased natural capital |
|  | Decreasing negative environmental exposures | Decreased pollution  Decreased exposure to hazards |
| DISEASE SUSCEPTIBILITY | Affecting disease patterns | Reduced environmental risk of certain diseases  Changes in disease distribution  Increased resilience to disease |
|  | Altering generational transmission | Changes in transgenerational programming  Intergenerational genetic adaptation  Changes in penetrance of specific traits  Stabilising selection |

## **1.3 Social processes**

| Categories | Sub-categories | Mechanisms |
| --- | --- | --- |
| MICRO-LEVEL | | |
| SOCIAL ACTIVITY | Increasing social contact | Increased frequency of social contact  Increased unmediated interactions  Increased social integration  Increased social engagement  Increased cultural mixing |
|  | Stimulating social actions | Increased social mimicry  Changes in social influence  Changes in social contagion  Audience effects  Increased social performance  Increased prosocial behaviour  Increased altruism |
| SOCIAL RELATIONSHIPS | Enhancing social engagement | Increased positive attitude towards social exchange  Increased social networks  Stronger network ties  Greater satisfaction of social needs  Reduced iso-strain |
|  | Supporting social bonding | Increased reciprocity  Increased emotional closeness  Increased social bonding  Improved attachment styles  Increased satisfaction of desire for attachment  Reduced social isolation  Reduced loneliness  Decreased alienation  Increased opportunity for solitude  Increased belonging  Achievement of biosocial goals |
| LEARNING AND TRAITS | Building social learning and traits | Improved social skills  Reduced othering  Reduced individualism  Enhanced civic individualism  Increased social trust  Increased social responsibility  Changes in framing |
|  | Building cultural learning and traits | Increased cultural attachment  Increased cultural learning  Increased cultural consonance  Increased acculturation  Reduced acculturative stress  Increased inter-cultural competence  Increased cultural embodiment |
| SOCIAL RESOURCES | Building social identity | Changes in anonymity  Development of an interdependent self  Development of social identity  Development and reinforcement of social roles  Validation of experiences  Increased feeling of being valued  Increased positive social comparisons  Heightened social status |
|  | Building capital | Increased social support  Increased social capital  Increased employability  Increased physical capital  Increased socio-economic position  Increased social conversion factors  Increased environmental conversion factors |
| MESO/MACRO-LEVEL | | |
| GROUP STRENGTH | Supporting group cohesion | Enhanced social surroundings  Increased mutual dependence  Enhanced social solidarity  Enhanced collectivism  Increased social cohesion  Increased community cohesion  Decreased ostracism  Increased shared achievements |
|  | Building integration & resilience | Reduced marginalisation  Decreased cultural homogenisation  Increased normative multi-culturalism  Preservation of cultural traditions  Increased polyculturalism  Improved social resilience  Improved community resilience |
| GROUP POWER | Disrupting hierarchies | Changes in social hierarchies  Reduced social dominance  Increased social mobility  Social change  Increased community empowerment  Changes in social power  Increasing cultural evolution |
|  | Improving equality | Increase in social justice  Decreased relative deprivation  Decreased area deprivation  Increased neighbourhood security  Reduced income inequality  Reduced social inequality  Increased health equity |
|  | Developing the leisure industry | Increased leisure industrialisation  Increased numbers of jobs within leisure  Changes in labour market structure  Improvements in economic stability & security  Development of leisure-related public policies |

## **1.4 Behavioural processes**

| Categories | Sub-categories | Mechanisms |
| --- | --- | --- |
| MICRO-LEVEL |  |  |
| DEVELOPMENT OF HABIT | Disrupting existing habits | Disruption of autopilot  Discontinuity of habits  Disruption of script  Disrupted status quo bias  Reduced psychological reactance to norms |
|  | Assisting in the formation of new habits | Operant learning  Classical conditioning  Performing action slips  Formation of habits  Reinforcement of behaviours  Increased continuity of positive behaviours  Development of harmonious addiction |
| BEHAVIOURAL DECISIONS | Influencing individual choice | Reduced cognitive dissonance  Decreased dynamic inconsistency  Reduced cognitive bias  Reduced disconfirmation bias  Increased considerations underlying choice  Increased balancing of outcome expectancies  Increased reasoned action  Increased controlled risk-taking  Changes in perception of boundaries |
|  | Supporting the prediction of behaviours | Changes in default effects  Changes in deadline effects  Changes in norm effects  Changes in self-fulfilling prophecies  Changes in behavioural prediction  Increased response inhibition  Enhanced inhibitory control |
| BEHAVIOURAL DRIVE | Enhancing behavioural activation | Unfreezing of behaviours  Increased initiative  Increased goal setting  Increased self-activation  Increased self-efficacy  Increased readiness to act  Increased mental simulation  Increased action tendency  Increased self-determination  Decreased ego depletion  Decreased self-sabotaging beliefs |
|  | Increasing motivation | Decreased apathy  Increased extrinsic motivation  Increased intrinsic motivation  Increased expectancy motivation  Increased task-based motivation  Increased identity-based motivation  Reduced fear of failure  Anticipated regret from non-engagement  Increased evaluation apprehension  Increased implementation intentions  Perception of being given a second chance |
|  | Supporting achievement | Increased sense of reward  Increased commitment  Increased perseverance  Increased goal attainment  Increased experience of making mistakes  Improved personal competence  Increased self-actualisation  Increased generativity |
| BEHAVIOURAL DEVELOPMENT | Supporting child development | Improved infant behaviours  Increased social referencing  Enhanced school readiness  Reduced truancy  Improved parenting practices  Increased play |
|  | Supporting behavioural adjustment | Reduced externalising behaviours  Reduced internalising behaviours  Improved development of childhood adaptive behaviours  Reduced bullying  Increased independence |
| PERSONAL LOCATION | Enhancing a sense of time | Increased flow  Increased sense of momentum  Decreased boredom  Provision of routine  Displacement of time to engage in unhealthy behaviours  Changes in perception of time  Reduced present bias  Increased anticipation of forthcoming positive events  Increased prospection |
|  | Enhancing a sense of place | Enhanced sense of place  Development of a safe space  Increased perceptions of safety  Enhanced attachment to place  Enhanced field of care  Changes in place-based behaviour  Increased local engagement  Expanded life-world |
| MESO/MACRO-LEVEL | | |
| COOPERATION | Increasing social control | Changes in patterns of herd behaviour  Enactment of ritual  Changes in social shaping  Changes in social regulation of effort  Changes in levels of territoriality  Changes in informed social control  Reduced social strain |
|  | Building group learning | Increased social learning  Reduced bandwagon effect  Increased cooperation  Increased cultural consonance  Increased collective intentionality  Ecological transition |
|  | Encouraging adaptive group behaviours | Engagement in collective projects  Increased active citizenship  Increased collective action  Development of social movements  Increased political participation  Increased sharing of resources  Changes in civil unrest  Decreased conflict  Reduced criminality |
| APPROACHES TO HEALTH | Increasing social responsibility | Altered social conception of illness  Increased assumption of social responsibility  Increased collective responsibility in health  Increased social accountability towards health |
|  | Increasing health promotion | Increased collective self-efficacy  Increased structuralist-collectivist health promotion  Improved social-ecological health promotion  Changes in adaptation behaviours |
| AVAILABILITY OF ASSETS | Increasing leisure assets | Increased number of leisure artefacts  Increased diversity of leisure artefacts  Increased number of leisure experiences  Increased diversity of leisure experiences  Increased number of community assets  Increased diversity of community assets  Increased geographical spread of community assets  Increased preservation of community assets  Increased placemaking |
|  | Increasing healthcare assets | Increased numbers of paraprofessionals  Increased availability of self-help groups  Increased availability of leisure-based activities to support health  Increased referral systems to leisure activities |

## **1.5 Health behaviours**

| Categories | Sub-categories | Mechanisms |
| --- | --- | --- |
| MICRO-LEVEL | | |
| ENGAGEMENT IN HEALTHY BEHAVIOURS | Increasing behaviours relating to the prevention of ill-health | Increased leisure engagement  Reduced sedentary behaviours  Increased physical activity  Improved sleep  Increased engagement with behavioural immunogens  Repeated healthy behaviours  Increased responsiveness to health communication |
|  | Increasing behaviours relating to the management of ill-health | Increased self-management of health  Increased treatment adherence  Reduced need for medical treatment  Avoidance of relapse  Changes in use of health services  Increased self-protection behaviours  Provision of informal care |
|  | Developing behaviours to support the delivery of healthcare | Improved clinical skills  Improved staff-patient interactions  Reduced clinical errors  Reduced staff burnout |
| DISENGAGEMENT IN UNHEALTHY BEHAVIOURS | Reducing engagement in unhealthy activities | Decreased unhealthy behaviours  Decreased problem behaviours  Decreased delinquent or deviant behaviour  Decreased unsafe sex practices  Decreased screen time |
|  | Reducing engagement with substances | Decreased smoking and tobacco use  Decreased drug use  Improved management of addiction |
| MESO/MACRO-LEVEL | | |
| HEALTHCARE | Influencing healthcare delivery | Enhanced therapeutic landscapes  Increased de-institutionalisation  Increased primordial prevention  Improved health communication  Increased availability of person-centred care  Reduced demand for healthcare services |
|  | Improving healthcare performance | Increased recognition of paraprofessionals  Disruption of healthcare societies  Rehumanisation of health  Development of more compassionate care environments  Enhanced performance & quality of healthcare  Enhanced mutual recovery |
|  | Removing barriers to healthcare | Reduced health discrimination  Increased engagement with hard-to-reach groups  Increased conversations about health  Decreased accumulation of health hazards  Decreased clustering of negative health behaviours  Reduced health inequities  Reduced health inequalities |

# **Psychological Processes: in detail**

## **2.1 Micro-level**

| Mechanism | Definition | Reference |
| --- | --- | --- |
| AFFECTIVE STATES |  |  |
| Eliciting affective responses |  |  |
| Increased experience of positive emotions | Pleasant or desirable situational responses ranging from interest and contentment to love and joy | ^1^ |
| Decreased experience of negative emotions | Unpleasant situational responses such as hate, anger, jealousy and sadness | ^1^ |
| Decreased stress | A state of bodily or mental tension resulting from factors that alter an existent equilibrium | ^2^ |
| Increased eustress | A positive form of stress that has beneficial effects on health, motivation, performance and wellbeing | ^2^ |
| Changes in valence | An evaluation of an experience as intrinsically attractive (positive valence) or averse (negative valence) | ^3^ |
| Experience of pleasure | Agreeable or enjoyable feelings that can form the basis for more elaborate emotions or evaluations | ^4^ |
| Supporting emotion regulation |  |  |
| Improved attention-focused emotion regulation | The regulation of emotions via techniques that deploy attention (e.g. concentration, distraction or suppression) | ^5,6^ |
| Improved knowledge-focused emotion regulation | The regulation of emotions via techniques that involve cognitive change (e.g. reflection or reappraisal) | ^5,6^ |
| Improved expression-focused emotion regulation | The regulation of emotions via techniques such as venting or physiological self-regulation | ^5,6^ |
| Increased self-soothing | The ability of an individual to comfort themselves without the need for others | ^7^ |
| Changes in mood | Pervasive, sustained and less-specific feelings, states or prolonged emotions | ^8^ |
| Broadening of momentary thought-action repertoire | Whereby the experience of positive emotions such as joy, interest, contentment and love sparks a recurring cycle of positive emotions (see also broaden-and-build theory) | ^9^ |
| RESILIENCE |  |  |
| Supporting coping |  |  |
| Increased emotion-focused coping | The management of external or internal demands that are appraised as taxing or exceeding one’s resources through dealing with the emotional response | ^10^ |
| Increased problem-focused coping | The management of external or internal demands that are appraised as taxing or exceeding one’s resources through looking at solutions | ^10^ |
| Increased stress buffering | The reduction of the impact of life stress on oneself through the protective effect of psychosocial resources or engagement in diversified activities | ^11,12^ |
| Decreased catastrophizing | The tendency to irrationally believe something is far worse than it actually is (see also awfulising) | ^13^ |
| Reduced suicidal ideation | Thinking about, considering or planning suicide | ^14^ |
| Increased terror management | Whereby one overcomes one’s fear of adverse events (e.g. death) through pursuing activities that promote self-esteem | ^15^ |
| Improved management of grief | The response to loss, particularly to the loss of someone or something to which a bond or affection was held | ^16^ |
| Development of supportive internal dialogue | The voice in one’s head that comments on life, events and one’s thoughts either consciously or sub-consciously (see also use of self-talk) | ^17^ |
| Increased adjustment | One’s ability to find a resolution to a situation through achieving coordinated balance across multiple domains including feelings and actions | ^18^ |
| Increased accommodation | Whereby one is able to manage changes in their life (such as illness) through the pursuit of modified goals | ^18^ |
| Greater availability of cognitive bandwidth | Whereby the regulation of emotions frees up mental space and thereby leads to more rational thinking and behaviours (see also bounded rationality) | ^19^ |
| Building psychological strength |  |  |
| Increased self-confidence | A feeling of trust in one's abilities, qualities, and judgement | ^20^ |
| Increased resilience | One’s ability to recover from or adjust easily to change | ^21^ |
| Increased vitality | A state of being full of life and both mental and physical energy | ^22^ |
| Improved psychic homeostasis | A state of emotional balance that can be markedly altered during psychic disturbances | ^23^ |
| Increased sense of continuity | A sense of maintaining patterns and behaviours from the past in the face of disruptive unexpected and unwanted change | ^24^ |
| Improved tolerance of uncertainty | The assessment of uncertainty as desirable versus threatening | ^25^ |
| Decreased submissiveness | Whereby one obeys or yields to another’s will, putting one’s own desires lower than another’s | ^26^ |
| Decreased denial | An attempt to fight against a situation by minimising events and/or the implications of events (see also crisis theory) | ^18,27^ |
| Decreased resignation | Whereby one feels overwhelmed by events and feels subject to vicissitudes | ^18^ |
| Reduced subjective weathering | A social psychological (perceived) component of accelerated ageing | ^28^ |
| Increased conservation of resources | Whereby one seeks to obtain, retain and protect individual resources (e.g. self-esteem and control), in particular in the face of loss | ^29^ |
| SENSE OF SELF |  |  |
| Building identity |  |  |
| Formation & affirmation of self-identity | The perception or recognition of one's characteristics as a particular individual | ^30^ |
| Improved self-knowledge | Understanding of oneself or one's own motives or character | ^31^ |
| Improved self-concept | The perception or mental image one has of oneself | ^32^ |
| Decreased self-discrepancy | Whereby discrepancies between different self-beliefs or self-state representations produce emotional vulnerabilities | ^33^ |
| Decreased de-individuation | Whereby one loses one’s sense of individual identity | ^34^ |
| Increased self-expression | The expression of one's feelings, thoughts, or ideas (see also voice) | ^35^ |
| Development of a future self | How one imagines oneself in the future and the degree of continuity between a present self and that future self | ^36^ |
| Increased self-awareness | An awareness of one's own personality or individuality | ^37^ |
| Developing self-acceptance |  |  |
| Increased self-esteem | One’s sense of self-worth or personal value (either general or domain specific such as body-related self-esteem) | ^38^ |
| Increased self-enhancement | Whereby an individual places emphasis on their virtues ahead of their shortcomings | ^11^ |
| Increased self-protection | Whereby an individual construes events in a way that places their attributes in a favourable light | ^11^ |
| Reduced self-stigmatisation | Whereby the sense of self projected by others negatively affects one’s perception of oneself | ^39^ |
| Increased self-affirmation | The ability to affirm one’s own worthiness and value in the face of information or experiences that threaten one’s self concept | ^40^ |
| Decreased self-derogation | The tendency to disparage oneself, often unrealistically (see also self-damning) | ^41^ |
| Increased authenticity | The ability to be true to oneself and one’s beliefs | ^42^ |
| Acceptance of personal fallibility | Whereby one comes to terms with and embraces one’s tendency to make mistakes or be wrong such that they do not disrupt one’s sense of self | ^43^ |
| PERSONAL TRANSFORMATION |  |  |
| Supporting changing identity |  |  |
| Reduced ‘loss of self’ | A loss of subjective self-identity sometimes experienced in the face of an illness or other adverse event | ^44^ |
| Reduced biographical disruption | The way that one makes sense of an illness or other adverse event in the context of their lives | ^45^ |
| Increased reminiscence | The act of recollecting past experiences or events | ^46^ |
| Increased self-restoration | Re-affirmation of a previous identity and reinforcement of valued aspects of one’s previous life (e.g. prior to illness) | ^24^ |
| Provision of alternative identities | Whereby an individual can define themselves both according to multiple different factors in their life (e.g. assuming a ‘sick role’ but also holding an identity relating to their leisure engagement) | ^47^ |
| Building of new narratives | The way one views and understands one’s life story. Contingent narratives address beliefs about the origins of an event (e.g. illness), while moral narratives help to constitute changes between an individual, their illness and their identity, and core narratives connect an individual’s experience with deeper cultural levels of meaning | ^48^ |
| Development of personal pathography | The formulation of an understanding of one’s experience of illness (or other adverse event) | ^49^ |
| Aiding personal evolution |  |  |
| Increased openness to experience | A personality trait involving characteristics such as imagination and insight | ^50^ |
| Increased agreeableness | A personality trait involving characteristics such as trust, altruism, kindness, affection and other prosocial behaviours | ^50^ |
| Increased conscientiousness | A personality trait involving characteristics such as thoughtfulness, good impulse control, and goal-directed behaviours | ^50^ |
| Increased extroversion | A personality trait involving characteristics such as excitability, sociability, talkativeness, assertiveness, and high amounts of emotional expressiveness | ^50^ |
| Decreased neuroticism | A personality trait involving characteristics such as sadness, moodiness, and emotional instability | ^50^ |
| Increased optimism | An inclination to put the most favourable construction upon future events or actions | ^24^ |
| Personal transformation | Changes in one’s life such as restructuring of life goals and commitments, new activity interests and greater attention to the present in the wake of negative life events | ^24^ |
| Increased growth | Personal change and development associated with life events | ^51^ |
| Increased regulatory focus | Whereby an individual works to bring oneself into alignment with one’s standards and achieve one’s goals | ^52^ |
| Self-transcendence | A decreasing reliance on external factors for one’s definition of self, increasing interiority and spirituality, and a greater sense of connectedness with past and future | ^53^ |
| FLOURISHING |  |  |
| Enhancing meaning in life |  |  |
| Provision of meaning | Whereby an individual feels their life has significance | ^54^ |
| Increased sense of life being worthwhile | A feeling that one’s life is useful, important or good enough | ^54^ |
| Improved life satisfaction | A favourable attitude towards one’s life | ^54^ |
| Increased sense of coherence | How manageable, meaningful and comprehensible one finds the world to be | ^55^ |
| Formation and affirmation of central values | Principles or standards of behaviour and judgement of what is important in life | ^56^ |
| Increased gratitude | The quality of being thankful and a readiness to show appreciation for and to return kindness | ^57^ |
| Increased purpose | The courage to envisage and pursue valued goals | ^58^ |
| Developing autonomy |  |  |
| Increased self-locus of control | The ability to regulate oneself and the world around (internal locus of control) rather than events being due to chance or others (external locus of control) |  |
| Changes in self-attribution | The extent to which one sees oneself as responsible for situations and events as opposed to blaming failures on factors outside of our control (see also self-serving bias) | ^59^ |
| Increased autonomy | Self-directing freedom and independence | ^60^ |
| Increased agency | The capacity to act on one’s own behalf | ^61^ |
| Increased empowerment | Personal control and ability to effect change | ^62^ |
| Compensation for imbalanced demand-control in other life domains | Whereby an individual feels high psychological demands but low control in settings unrelated to leisure (such as work settings) | ^63^ |
| PSYCHOLOGICAL CAPABILITIES | |  |
| Supporting cognitive learning |  |  |
| Enhanced information processing | The ability to retain information over time through encoding, storage and retrieval | ^64^ |
| Enhanced semantic memory | Knowledge of facts, concepts, words, definitions, and language rules | ^64^ |
| Enhanced episodic memory | Knowledge of specific events, personal experiences (episodes), or activities | ^64^ |
| Enhanced procedural memory | Memory for motor and some cognitive skills, as well as emotional behaviours learnt through conditioning | ^64^ |
| Enhanced convergent thinking | The weighing of alternatives in solving a problem or answering a question | ^65^ |
| Enhanced divergent thinking | Creative thinking that leads to generation of new and original solutions to problems | ^65^ |
| Improved reasoning | A mental process that involves using and applying knowledge to solve problems/achieve goals | ^64^ |
| Expansion of perceptual sets | One’s tendency to perceive or notice some aspects of available sensory data and ignore others (see also auditory discrimination) | ^66^ |
| Increased use of heuristics | Practical mental shortcuts that ease the cognitive load of making decisions | ^67^ |
| Enhanced transfer effects | Whereby the knowledge and skills learnt in one cognitive domain have an effect on the achievement of goals in other cognitive domains (see also non-trained cognitive functions) | ^68^ |
| Enhanced aesthetic judgement | Sensory, emotional and intellectual responses to and reflections on art, culture and nature | ^69^ |
| Enhanced attention | The cognitive-behavioural process of concentrating specifically on one stimulus while ignoring other stimuli | ^64^ |
| Supporting emotional learning |  |  |
| Increased emotion recognition | The ability to recognise emotions being experienced or expressed by others | ^70^ |
| Improved theory of mind | The ability to attribute mental states to oneself and to others, and to understand that others’ beliefs, thoughts and perspectives might be different to one's own | ^71^ |
| Increased empathy | The awareness, understanding, sensitivity to, and vicarious experiencing of someone else’s thoughts, feelings, and experiences | ^72^ ^73^ |
| Increased compassion | Sympathetic pity and concern for the challenges faced by others | ^74^ |
| Enhanced sense of conscience | One’s moral sense of right and wrong, which acts as a guide to one's behaviour | ^75^ |
| Increased decentering | The ability to consider multiple aspects of a situation, moving from egocentrism to a shared reality with others (see also decentration) | ^76^ |
| Increased mentalising | The ability to see ourselves as others see us, and others as they see themselves | ^77^ |
| Increased mind-mindedness | One’s tendency to view another (especially a child) as an individual with a mind | ^78^ |
| Supporting overarching cognitive processes |  |  |
| Enhanced processing fluency | The ease with which information is processed | ^79^ |
| Enhanced cognitive development | The process by which one acquires knowledge and intelligence through changes in cognitive processes and abilities across childhood and into adulthood | ^80^ |
| Enhanced cognitive restructuring | The identification of problematic cognitions and distortions and the rationalisation and rebuttal of these thoughts | ^81^ |
| Enhanced transformative learning | The expansion of consciousness and change of frames of reference through transforming one’s understanding of oneself, a revision of one’s belief systems, and changes in lifestyle | ^82^ |
| Increased diversification of intelligence | Intelligence has been proposed to have at least nine different types: verbal, musical, logical-mathematical, spatial, body movement, intelligence to understand oneself, intelligence to understand others, naturalistic intelligence, and existential intelligence | ^83^ |
| Increased curiosity | The urge to know or learn | ^84^ |
| Enhanced creativity | A combination of flexibility in thinking and reorganisation of understanding to produce innovative ideas and new or novel solutions | ^85^ |
| Enhanced visualisation | The formation of a mental image of an object, situation or set of information | ^86^ |
| Increased imagination | The process of forming new ideas or images of something that cannot be perceived at that moment by the senses | ^87^ |
| Reduced cognitive decline | Problems with memory, language, thinking or judgement that occur with ageing and can develop into cognitive impairment and dementias | ^88^ |
| PSYCHOLOGICAL RESOURCES |  |  |
| Building general resources |  |  |
| Enhanced maturity | The awareness of how to behave and act according to the circumstances of the environment and the culture or the society in which one lives | ^89^ |
| Increased human capital | Individual resources including knowledge, skills, abilities, talents, intelligence, values | ^90^ |
| Increased cultural capital | Symbolic and embodied resources related to taste, engagement with art, language etc. that indicate social status or ability to have social mobility | ^90^ |
| Improved educational attainment | The highest level of education that an individual has completed | ^91^ |
| Increased personal conversion factors | One’s ability to convert resources (such as knowledge) into functionings | ^92^ |
| Building health-related resources |  |  |
| Expanded lay theories | One’s basic assumptions about what health is and what it is influenced by | ^93^ |
| Changes in illness cognitions | Individuals’ beliefs about their own illnesses (especially seen in individuals with a chronic illness) | ^94^ |
| Increased health consciousness | Whereby one becomes increasingly aware of one’s health, diet and lifestyle | ^95^ |
| Increased health literacy | The degree to which one has the capacity to obtain, process, and understand basic health information so that one can make appropriate health decisions | ^96^ |
| Increased understanding of others’ health conditions | The degree to which one comprehends the experience of living with mental or physical illness without resorting to stereotypes or essentialism | ^97^ |
| Increased salience of healthcare engagement | Whereby one perceives engaging with healthcare to be more important or relevant to oneself through it being highlighted alongside leisure engagement | ^98^ |
| Experience of healing | Whereby an individual moves from feeling ill to feeling well, balanced, restored, whole, and vitalised; an experience that can go beyond the resolution of specific medical symptoms | ^99^ |
| Improved quality of life | The standard of health, comfort, and happiness experienced by an individual or group | ^100^ |

## **2.2 Meso-/Macro-level**

| Mechanism | Definition | Reference |
| --- | --- | --- |
| GROUP MIND |  |  |
| Developing a group self |  |  |
| Increased sense of group self | A collective self with inherent ambitions, ideals and resources (see also collective self-definition) | ^101^ |
| Increased collective rationality | Rational cooperation guided by group reasoning that results in a collaborative effort to achieve or solve something | ^102^ |
| Altered cultural perception | The way that groups of people from a particular culture perceive their social environment | ^103^ |
| Increased group knowing | The building of collaborative knowledge | ^104^ |
| Enhanced sense-making | The situated, embodied activity of learning through interaction between learners and their environment (see also enactivist theory) | ^105^ |
| Developing group values and understanding |  |  |
| Increased meaning-making | Social understandings of identity, difference, morals, truth, perception and aesthetics (see also social semiotics) | ^106^ |
| Development of social norms | The unwritten rules of behaviour considered acceptable in a group or society | ^107^ |
| Reduced anomie | A condition of instability within society resulting from a lack of group purpose or ideals or a breakdown of collective standards or values | ^108,109^ |
| Increased collective responsibility | Whereby individuals feel responsible for others’ actions | ^110^ |
| Development of collective consciousness | The acquisition of critical consciousness as groups understand events around them (see also conscientisation) | ^111^ |
| GROUP ATTITUDES |  |  |
| Reducing stigma |  |  |
| Reduced social labelling | A process by which an individual or group obtain labels from how others view their tendencies or behaviours, which can in turn affect individuals’ identities and behaviours (see also stereotyping) | ^112^ |
| Reduced prejudice | Irrational and often inflexible opinions or attitudes held by one group about another | ^113^ |
| Increased tolerance | The ability or willingness of a group to tolerate opinions or behaviours that the group dislikes or disagrees with | ^113^ |
| Disruption of stereotypes | Oversimplified images or ideas about individuals or other entities that are widely held | ^113^ |
| Decreased social death | Whereby individuals with certain characteristics or health conditions (such as dementia) are not accepted as fully human by wider society and feel a loss of identity, connectedness and integration | ^114^ |
| Changing attitudes to health |  |  |
| Changes in the social construction of illness | How individuals come to understand health and illness based on how these concepts are embedded within cultural meaning | ^115^ |
| Decreased medicalisation of social deviance | Whereby problems such as obesity, heavy drinking and smoking are seen as medical rather than social problems | ^116^ |
| Decreased cultural camouflage | Whereby cultural stereotypes are used to excuse unhealthy behaviours such as drinking and smoking | ^117^ |
| Decreased risk discourse | Whereby individuals with health conditions (such as mental illness) are seen first in terms of the risks they pose to themselves and others as opposed to the care and support they need | ^118^ |
| Enhanced satisfaction with healthcare | Positive reporting of experiences by patients and healthcare staff | ^119^ |
| LANGUAGE |  |  |
| Changing communication |  |  |
| Increased voice for minority groups | A group of people who experience collective discrimination or unequal treatment because of their physical or cultural characteristics | ^120^ |
| Enhanced group matrix | The conscious and unconscious communicational network in a group | ^121^ |
| Increased spontaneous communication | The non-intentional communication of motivational-emotional states through biologically-shared understanding of displays (see also non-verbal communication) | ^122^ |
| Increased symbolic communication | The intentional communication of information using learned, socially shared signal systems (see also non-verbal communication) | ^122^ |
| Development of memes | Elements of a culture that propagate successfully and have the power to communication information widely and influence opinions | ^123^ |
| Increased symbolic interactionism | The interaction of individuals in a group via linguistic or gestural communication that builds social meaning | ^124^ |

| Modifying group emotion |  |  |
| --- | --- | --- |
| Elicitation of collective emotions | Whereby individuals collectively express group-based sentiments | ^125^ |
| Increased collective emotional convergence | The social calibration of emotional experiences | ^125^ |
| Decreased collective stress | Whereby social suffering at a group level leads to an increase in population stress | ^126^ |
| Formation of collective memories | The development of memories through involvement in identical emotionally relevant events, which heightens the propensity for recurring emotional convergence | ^125^ |
| Development of collective effervescence | Whereby a group comes together and simultaneously communicates the same thought or participates in the same action, leading to collective excitement and unification | ^127^ |

# **Biological Processes: in detail**

## **3.1 Micro-level**

| Mechanism | Definition | Reference |
| --- | --- | --- |
| NERVOUS SYSTEM |  |  |
| Activating perceptual processes |  |  |
| Changes in activation of the auditory system | The process by which sensations transduced in the ear from our surroundings stimulate nerves leading to the perception of sound | ^128^ |
| Changes in activation of the visual system | The process by which photoreceptors detect and interpret information from visible light to build a representation of the surrounding environment | ^128^ |
| Changes in activation of the somatosensory system | The process by which sensory receptor cells respond to changes at the surface or inside of the body and stimulate neural pathways leading to the perception of pain, temperature, touch and both position and movement | ^128^ |
| Changes in activation of the gustatory system | The process by which sensations produced in the mouth stimulate nerves leading to the perception of taste | ^128^ |
| Changes in activation of the olfactory system | The process by which sensations produced in the nostrils and surrounding areas stimulate nerves leading to the perception of smell | ^128^ |
| Changes in activation of the vestibular system | The process by which sensory organs next to the cochlea in the inner ear enable us to maintain balance and body posture | ^128^ |
| Increased proprioception | The perception or cognitive awareness of the position of one’s body in space | ^129^ |
| Increased interoception | The awareness and understanding of what is happening inside one’s own body (the sense of the internal state of the body) | ^130^ |
| Increased kinaesthesia | The awareness of movements in different parts of the body using sensory organs in the muscles and joints; a key component in hand-eye coordination and muscle memory | ^131^ |
| Increased perceptual sensitivity | The amount one can detect slight, low-intensity stimuli from the external environment | ^132^ |
| Increased sensory equilibrium | The adjustment of the senses to relieve external stress and establish balance | ^133^ |
| Modulated levels of sensory deprivation | The process by which one is deprived of normal external stimuli such as sight and sound for an extended period of time | ^134^ |
| Increased multisensory integration | The neural integration or combination of information from the different senses (see also multimodal integration or multisensory activation) | ^135^ |
| Decreased central & peripheral sensitisation | Whereby the central and peripheral nervous systems become regulated in a persistent state of high reactivity leading to the development and maintenance of chronic pain | ^136^ |
| Reduced pain | Unpleasant sensory and emotional experience associated with actual or potential tissue damage. It is asserted that non-painful sensory input can prevent pain sensations from travelling to the nervous system (see also gate-control theory of pain) | ^137^ |
| Increasing brain activation |  |  |
| Increased overall cerebral activation | Whereby the engagement of specific parts of the brain leads to increased oxygen delivery beyond the actual metabolic demand, which can support cognitive processes | ^138^ |
| Improved overall cerebral blood flow | The blood supply to the brain in a given period of time, dysregulation of which is associated with problems such as hypertension and risk of stroke and dementias | ^139,140^ |
| Increased activation of brain regions involved in basic vision and object perception | This includes brain regions such as the occipital cortex, fusiform gyrus, and parahippocampal gyrus | ^141,142^ |
| Increased activation of brain regions involved in attention and sensory processing | This includes regions such as the fusiform gyrus, angular gyrus, and superior parietal cortex | ^141,142^ |
| Increased activation of brain regions involved in knowledge and meaning | This includes regions involved in evaluative judgement and information retrieval (e.g. the dorsolateral, ventrolateral, anterior medial prefrontal cortex, temporal pole, posterior cingulate and precuneus) | ^141,142^ |
| Increased activation of brain regions involved in reward, valuation or response to perceived beauty | This includes regions such as the anterior cingulate cortex, orbitol frontal cortex, insula, ventromedial prefrontal cortex, caudate nucleus, substantia nigra, nucleus accumbens, and posterior cingulate cortex | ^141,142^ |
| Increased activation of brain regions involved in affective responses | This includes regions such as the subcortical amygdala, insula, posterior cingulate cortex, and medial temporal lobe | ^141,142^ |
| Increased activation of the motor cortex | A region of the cerebral cortex involved in the planning, control, and execution of voluntary movements, which includes the premotor areas, the primary motor cortex and the supplementary motor area | ^141,142^ |
| Activation of mirror neurons | Neurons that fire both when one acts and when one observes another carrying out an action, supporting in processes such as empathy, mimicry and synchronisation | ^143^ |
| Altering brain physiology |  |  |
| Changes in brain structure | Changes in dynamic spatial and temporal patterns of brain development, which can be associated with changes in cognitive skills especially across the developmental period | ^144^ |
| Increased grey matter & grey matter integrity | Tissue in the brain composed of neuronal cell bodies and other cells that is found in regions of the brain such as those involved in muscle control, sensory perception, memory, emotions, speech, decision making and self-control | ^145^ |
| Increased white matter & white matter integrity | Tissue in the brain composed of nerve fibres, the integrity of which facilitates connection among distributed neural systems and is associated with good perceptual speed and executive functioning | ^145^ |
| Increased neurogenesis | The process by which neurons are produced by neural stem cells | ^146^ |
| Increased neuroplasticity | The ability of the brain to change continuously throughout an individual's life. Neuroplasticity is decreased by factors such as stress and depression via mediating biological mechanisms such as decreases in brain derived neurotrophic factor (BDNF) | ^147^ |
| Decreased neurotoxicity | Damage to the brain or peripheral nervous system caused by toxins such as stress hormones | ^148^ |
| Reduced brain atrophy | Changes to the brain including suppressed proliferation of neurons, alterations in the morphology of neurons and reductions in the volume of the different brain regions as a result of factors such as stress | ^148^ |
| Increased cognitive reserve | The resilience of the brain against cognitive decline, which helps to explain individual differences in susceptibility to age-related brain changes | ^149,150^ |
| Modulating brain biomarkers |  |  |
| Changes in acetylcholine production | A neurotransmitter involved in memory, muscle contraction, heart rate, arousal, cognition and memory | ^141,142^ |
| Increased dopamine production | A neurotransmitter in the brain involved in motivation, reward and pain | ^151,152^ |
| Increased serotonin levels | A neurotransmitter in the brain involved in cognition, reward, learning, memory and reducing symptoms of depression and anxiety | ^153^ |
| Changes in oxytocin and vasopressin levels | Biomarkers that play a role in social bonding, trust, generosity and reproduction | ^154,155^ |
| Increased levels of opioids | Neuropeptides and peptide hormones such as endorphins involved in feelings of euphoria, hormonal and metabolic responses | ^156,157^ |
| Increased levels of cannabinoids | Lipids that play a role in memory, pleasure and hunger | ^158^ |
| Increased levels of melatonin | A hormone that regulates circadian rhythms, stress response and has anti-inflammatory properties within the immune system (increased by exposure to daylight, especially through outdoors engagement) | ^159^ |
| Changes in GABA and glutamate levels | GABA (gamma-Aminobutyric acid; an inhibitory neurotransmitter involved in anxiety, alertness, memory, muscle tension and sleep) and glutamate (an excitatory neurotransmitter involved in synaptic plasticity, learning and memory); both major neurotransmitters | ^160^ |
| Decreased neuro-inflammation | Inflammation of the nervous tissue, often triggered by processes such as ageing, exposure to viruses, air pollution, traumatic brain injury, toxic metabolites, and microbes. It can underlie conditions such as depression, anxiety and pain and is modulated by a range of psychosocial factors | ^161^ |
| Reduced disruption of biological rhythms | Natural internal processes that regulates physical, mental and behavioural changes and are affected by psychological, biological, social and behavioural processes, such as circadian rhythms that regulate each daily cycle | ^162^ |
| Modifying arousal |  |  |
| Changes in arousal levels | A measure of physiological activation ranging from feelings of energy and vigour to the opposite feelings of sleepiness and tiredness, and from subjective tension to placidity and quietness. Arousal underlies a variety of emotions and stress reactions | ^3^ |
| Increased chills | Skin tingling, piloerection and pupil dilation that can occur as a result of an emotional response to a stimulus | ^163^ |
| Changes in electrodermal activity | Differences in the electrical potential between different parts of the skin that give a measure of neutrally-mediated effects on sweat gland permeability in response to processes such as stress and arousal (see also skin conductivity) | ^164^ |
| Changes in activation of the autonomic nervous system | The system responsible for regulating the body’s unconscious actions. This has two divisions (the sympathetic and parasympathetic systems) that respectively respond to threat vs control homeostasis within the body, leading to a cascade of biological responses | ^165^ |
| Improved autonomic tone | The general activity rate of the autonomic nervous system and the balance between the sympathetic and parasympathetic aspects of the system | ^166^ |
| Changes in brainwaves | Neural oscillations (repetitive or rhythmic patterns of neural activity) such as alpha waves, beta waves and theta waves that affect physiological states such as arousal as well as processes such as memory and sleep | ^167^ |
| Brainwave entrainment | Whereby brainwave frequencies fall into step with a periodic stimulus leading to synchronisation of natural body functions and processes | ^168^ |
| Decreased muscle tension | Whereby muscles in the body remain semi-contracted for an extended period in response to psychological or physical factors | ^169^ |
| Increased nerve stimulation | Stimulation of peripheral or cranial nerves via electrical impulses, which has been found to reduce pain, depression and seizures | ^170^ |
| ENDOCRINE & IMMUNE SYSTEMS |  |  |
| Changing hormone levels |  |  |
| Improved regulation of the Hypothalamic-Pituitary-Adrenal axis | HPA axis; the central endocrine system for stress response that consists of the hypothalamus, pituitary gland and adrenal glands | ^171^ |
| Reductions in excessive glucocorticoid activity | The production of hormones such as vasopressin and cortisol which are increased in response to stress and are involved in processes including memory, mood, metabolism and wound healing | ^171^ |
| Reductions in excess production of catecholamines | Catecholamines act as neuromodulators in the brain and hormones in the blood to facilitate physiological responses such as changes in blood pressure and heart rate. Examples include adrenaline/epinephrine and noradrenaline/norepinephrine, which are part of the sympathetic nervous system’s response to stress | ^171^ |
| Changes in thyroid function | The thyroid gland is a part of the endocrine system and acts to regulate metabolism. Thyroid dysfunction can be caused by stress and is associated with a range of physical and psychological symptoms such as weight gain and anxiety | ^172^ |
| Changes in growth hormone levels | A hormone involved in growth, cell production and cell regeneration which is increased during acute physical stress and dysregulated by prolonged psychosocial stress | ^173^ |
| Changes in levels of sex hormones | Sex hormones include testosterone, oestrogen and progesterone, which are involved in functions including fertility, bone health and cardiovascular risk | ^174,175^ |
| Altering immune function |  |  |
| Changes in levels of innate immunity | Non-specific fast-acting defence mechanisms in the immune system. Multiple types of innate immune cells such as natural killer cells and neutrophils are increased by acute stress, but decreased by chronic stress | ^176^ |
| Changes in levels of acquired immunity | Adaptive defence mechanisms in the immune system that are specific to particular pathogens. Multiple types of acquired immune cells such as T lymphocytes are increased by acute stress, but decreased by chronic stress | ^176^ |
| Decreased cellular ageing | Progressive decline in the resistance of cells to stress and other cellular damages, causing a gradual loss of cellular functions and resulting eventually in cell death | ^177^ |
| Decreased bacterial and viral infections | Both the risk of common infections (e.g. common colds) and severe infections (e.g. sepsis, meningitis and endocarditis) is increased by factors such as stress | ^178^ |
| Changes in antibody levels | Antibodies (immunoglobulins) are molecules that enable the immune system to identify and neutralise foreign bodies such as viruses | ^176^ |
| Changes in levels of growth factors | Proteins or steroid hormones capable of stimulating the growth of immune cells. Growth factors are involved in a wide range of roles including cognition, stimulating the production of stem cells, and encouraging growth of new neurons in the brain | ^179^ |
| Decreases in levels of inflammation | Inflammatory markers include cytokines (chemical messengers that support communication between cells) and other biomarkers such as C-reactive protein (an acute phase protein). They are increased in response to acute stress and can become chronically elevated in response to chronic stress and depression | ^176,180^ |
| Shifts in cytokine profiles | Cytokines are small proteins that are important in cell signalling and activate immune cells and antibodies | ^176^ |
| Decreased oxidative stress | Oxidative stress is a disturbance in the balance between the production of reactive oxygen species (unstable molecules that contain oxygen) and antioxidant defences. It can lead to increased risk of a range of illnesses from cancers to dementias and cardiovascular conditions | ^181^ |
| Increased vitamin D levels | Vitamin D refers to a group of fat-soluble secosteroids (subclasses of steroids) responsible for multiple actions including increasing absorption within the intestines of calcium, magnesium, and phosphate. Vitamin D can be increased through engagement in outdoor activities that provide exposure to sun | ^182^ |

| CARDIOMETABOLIC SYSTEM |  |  |
| --- | --- | --- |
| Modulating cardiovascular factors |  |  |
| Reduced heart rate | The number of beats or contractions your heart makes per minute | ^183^ |
| Increased heart rate variability | The physiological phenomenon of the variation in the time interval between consecutive heartbeats, which is involved in biological responses to factors such as stress and emotions | ^184^ |
| Reduced vasoconstriction | The narrowing or constriction of blood vessels when smooth muscles in blood vessel walls tighten, which can be induced by stress | ^185^ |
| Decreased blood pressure | The force with which the heart pumps blood around the body, increased by physiological arousal and stress | ^186^ |
| Reduced hypertension | A medical condition whereby blood pressure in the arteries is persistently elevated, which is associated with a range of cardiovascular conditions | ^187^ |
| Changes in cardiovascular reactivity | The ability of the cardiovascular system to respond to periods of rest, demand or stress by changing heart rate, blood pressure or other measures of cardiovascular function | ^188^ |
| Increased angiogenesis | The formation of new blood vessels, which can be disturbed by psychosocial factors | ^189^ |
| Decreased aortic stiffness | Whereby the elastic fibres within the arterial wall (elastin) begin to fray due to mechanical stress; a process that is exacerbated by psychological stress | ^190^ |
| Improved cardiac function | The ability of the heart to meet the metabolic demands of the body | ^191^ |
| Modulating metabolic factors |  |  |
| Decreased glucose levels | Our main source of energy, carried through the bloodstream to provide energy to cells. Glucose levels are affected by factors such as stress | ^192^ |
| Changes in lipid levels | Hydrocarbon based molecules such as cholesterol (lipoproteins) and triglycerides that are essential for the structure and function of living cells, levels of which are affected by psychosocial processes | ^193,194^ |
| Changes in haemoglobin levels | Proteins responsible for transporting oxygen around the body. Levels of haemoglobin are affected by psychosocial processes such as stress and depression and both high and low levels (anaemia) can lead to physical illness | ^195^ |
| Decreased body mass | The ratio of a person's weight to their height, high levels of which are associated with a range of illnesses | ^196^ |
| Changes in body composition | Measures such as muscle mass or waist-hip ratio associated with diet and physical exercise | ^197^ |
| Improved gut microbiota | The gut is populated by biologically active microbes that interact with a range of processes in the host such as immune function, metabolism, organ development and microbiome composition. Gut microbiota have been linked to diet-induced disease predisposition and psychological processes | ^198^  ^199^ |
| Changes to the microbiome | The aggregate of all the microbes - bacteria, fungi, protozoa and viruses - that live on and inside the human body. It plays an important role in health by helping to control digestion and immune function and is affected by factors such as stress and mental illness | ^200^ |
| PERFORMANCE |  |  |
| Improving physical function |  |  |
| Improved balance | The distribution of weight that enables one to remain upright and steady | ^201^ |
| Improved gait | One’s manner of walking, including parameters such as velocity (speed), stride length and cadence (rhythm) | ^201^ |
| Improved reflexes | One’s basic unconscious physical responses to stimuli | ^201^ |
| Decreased reaction time | The length of time taken for a person to respond to a given stimulus or event | ^202^ |
| Improved flexibility | The range of motion in a joint or group of joints | ^201^ |
| Improved posture | The way in which one usually holds their shoulders, neck, and back | ^201^ |
| Increased bone health | Bone health includes factors such as bone density; a measure of the amount of minerals (mostly calcium and phosphorous) contained within bones | ^203^ |
| Improved limb function | One’s range of motion, strength and ability to use one’s arms and hands (upper limbs) or legs and feet (lower limbs) | ^201^ |
| Improved motor coordination | Combinations of motor movements that result in intended actions, including fine motor skills (smaller movements of wrists, hands, fingers and toes) and gross motor skills (larger movements involving arms, legs, feet or entire body) | ^201^ |
| Increased ability to perform activities of daily living | Essential and routine aspects of self-care, including independently eating, dressing, walking, bathing and using a toilet, and activities related to independent living such as managing money, taking medication, using a telephone and preparing meals | ^204^ |
| Increased physical fitness | One’s capacity for exertion or endurance, either via turning oxygen into energy for muscle cells (in aerobic exercise) or using energy stored in muscles (in anaerobic exercise) | ^201^ |
| Reduced falls | When one experiences an event leading to them coming to rest inadvertently on the ground | ^205^ |
| Improving respiratory function |  |  |
| Strengthened respiratory muscles | Diaphragm and external intercostal muscle contraction and ribcage elevation that lead to changes in volume and air pressure in the lungs | ^206^ |
| Increased lung capacity | The ability of the lungs to move air quickly through the airways via inhalation and exhalation (which is impaired in conditions such as asthma, chronic obstructive pulmonary disease and cystic fibrosis) | ^206^ |
| Improved structured respiratory variability | The ability of the respiratory system to respond to the oxygen demands of the body by varying factors such as breathing rate. Regulation of this system may be improved though physical activity | ^206^ |
| Improved diffusing capacity | The ability of the lungs to transfer gas from the air to red blood cells (see also transfer factor) | ^206^ |
| Increased oxygen saturation | The fraction of oxygen-saturated haemoglobin (a protein in red blood cells that carries oxygen around the body) relative to total haemoglobin in the blood | ^206^ |
| Improving voice |  |  |
| Improved speech | The expression of thoughts and feelings by articulate sounds. It involves factors such as phonation (ability to sustain the voice to achieve appropriate phrasing during speaking), fluency (e.g. not stuttering or stammering) and articulation | ^207^ |
| Improved language | The method of human communication involving the conveyance, comprehension, and processing of words (see also reduced aphasia) | ^207^ |
| Improved voice quality | The characteristic of auditory colouring of an individual’s voice involving factors such as roughness, breathiness, strain, deviations in pitch, deviations in normal volume, or unusual perceptual features such as instability and tremor | ^207^ |
| MULTI-SYSTEM |  |  |
| Reducing load |  |  |
| Increased homeostasis | A state of relatively stable equilibrium between physical and chemical conditions in the body | ^208^ |
| Changes in visceral factors | Drive states such as hunger, thirst, sexual desire, drug cravings, physical pain and fervent emotions | ^209^ |
| Reduced fatigue | Extreme tiredness resulting from mental or physical exertion or illness | ^210^ |
| Reduced allostatic load | The presence of too much stress or inefficient operation of the stress response system as a result of overload in trying to adapt to adverse psychosocial or physical situations | ^211^ |
| Rebalanced stress reactivity | One’s capacity or tendency to respond to stress, which, at high levels, can increase vulnerability to mental and physical illness | ^212^ |
| Reduced weathering | Accelerated psychological and physical ageing progresses as a result of cumulative exposure to stressful life circumstances (including socioeconomic disadvantage) and prolonged coping demands | ^213,214^ |
| Reduced biological embedding | Whereby an accumulation of biological processes over the life course leads to alterations in biological or developmental processes and poorer health | ^215^ |
| Decreased frailty | Physiological decline in late life that leads to vulnerability to adverse health outcomes | ^216^ |
| Increasing beneficial nature exposure |  |  |
| Enhanced exposure to microbial diversity | Exposure to microorganisms, such as bacteria, archaea, and eukaryotes, which is associated with improved human health | ^217,218^ |
| Enhanced exposure to phytoncides | Antimicrobial allelochemical volatile organic compounds derived from plants that have effects including improving immune response | ^219^ |
| Increased exposure to negative air ions | Electrically charged molecules in the atmosphere. They are created in nature as air molecules break apart through the movements of air and water and through sunlight and associated with multiple health benefits. | ^220^ |
| Reduced development of allergies and asthma | Exposure to high microbial diversity facilitates the development of an effective adaptive immune system and reduces risk of developing allergic diseases (see also the hygiene hypothesis) | ^218^ ^221^ |
| Affecting genetic / epigenetic factors |  |  |
| Reduced damage to telomeres | Non-coding, repetitive nucleotide segments on the ends of chromosomes that serve a protective role during DNA transcription and are shortened in length as a result of stress and ageing | ^222^ |
| Changes in DNA methylation patterns | The biological process whereby methyl groups (molecular structural units consisting of hydrogen and carbon atoms) are added to DNA molecules to repress gene expression. Particular patterns in methylation have been found in response to processes such as stress and adverse life experiences as well as enriched environments | ^223–225^ |
| Changes in gene expression | The processing of DNA into proteins that have biological functions. A range of molecular processes, such as DNA methylation, respond to stimuli (including psychological, social and behavioural activities) to change gene expression | ^226^ |

## **3.2 Meso-/macro-level**

| Mechanism | Definition | Reference |
| --- | --- | --- |
| ENVIRONMENTAL DIVERSITY |  |  |
| Increasing positive environmental exposures |  |  |
| Increased nature connection | As suggested by the biophilia (love of nature) hypothesis, humans have an inherent desire to connect with nature and natural processes (which may be increased at a group level through outdoors leisure engagement) | ^227^ |
| Increased biodiversity of spaces | The variability of animals, plants, fungi and microorganism species (which may be increased through increasing focus on and prioritisation of sustainable outdoor leisure activities) | ^228^ |
| Increased natural capital | The world’s stock of natural assets such as geology, soil, air, water and species (which may be increased or preserved through increasing focus on and prioritisation of sustainable outdoor leisure activities) | ^229^ |
| Decreased negative environmental exposures |  |  |
| Decreased pollution | Some plant and bacteria species are able to store (bioaccumulate) pollutants. So prioritisation of sustainable outdoor leisure activities could support high biodiversity, a nutrient rich ecosystem, and the removal of pollutants such as heavy metals or particulate matter | ^230,231^ |
| Decreased exposure to hazards | Hazardous factors include loud noise and waste (which can be reduced through improvement of spaces within neighbourhoods including leisure spaces) | ^232^ |
| DISEASE SUSCEPTIBILITY |  |  |
| Affecting disease patterns |  |  |
| Reduced environmental risk of certain diseases | The risk of certain infectious diseases is reduced through enhanced engagement with diverse ecosystems (which may be promoted through how we engage in outdoor leisure activities; see also dilution effect hypothesis) | ^233^ |
| Changes in disease distribution | The frequency and spatial patterning of communicable and non-communicable diseases (which may be directly affected by leisure behaviours, such as patterns of travelling abroad or staying at home during leisure time affecting communicable diseases, or indirectly via supporting health behaviours and thereby reducing non-communicable diseases) | ^234^ |
| Increased resilience to disease | Whereby changes in social, biological and behavioural factors at individual and group levels interact to provide changes in resistance to disease (see also ecosocial theory) | ^235^ |
| Altering generational transmission |  |  |
| Changes in transgenerational programming | Whereby environmental conditions and factors such as stress can influence disease risk across multiple generations through both non-genomic processes (e.g. behaviours) and epigenetic processes (e.g. fetal/prenatal programming) | ^236^ |
| Intergenerational genetic adaptation | Whereby humans continue to evolve biologically to adapt successfully to changing environments (which may be affected by persistent trends in leisure behaviours) | ^237^ |
| Changes in penetrance of specific traits | The proportion of individuals carrying a particular variant of a gene (genotype) associated with a particular trait (phenotype) (which may be affected by persistent trends in leisure behaviours) | ^238^ |
| Stabilising selection | Whereby over time selection favours the best suited genotype for the ecological niche (which may be affected by persistent trends in leisure behaviours) | ^239^ |

# **Social Processes: in detail**

## **4.1 Micro-level**

| Mechanism | Definition | Reference |
| --- | --- | --- |
| SOCIAL ACTIVITY |  |  |
| Increasing social contact |  |  |
| Increased frequency of social contact | Interaction with others, whether face-to-face, online, or via other means of communication | ^240^ |
| Increased unmediated interactions | Whereby one has the potential to meet strangers | ^241^ |
| Increased social integration | The process by which one is incorporated into the social structure of a society | ^240^ |
| Increased social engagement | One’s degree of participation in a community or society. It can be activated by mechanisms including attitudes, social influence, emotions and habits (see also theory of interpersonal behaviour) | ^242^ ^243^ |
| Increased cultural mixing | Whereby an individual of one culture moves into and interacts with another culture | ^244^ |
| Stimulating social actions |  |  |
| Increased social mimicry | The copying of postures, mannerisms, facial expressions and other behaviours to facilitate social reactions (see also chameleon effect) | ^245^ ^246^ |
| Changes in social influence | Whereby one changes one’s behaviour (either intentionally or unintentionally) to meet the demands of the social environment (see also peer pressure) | ^247^ |
| Changes in social contagion | Whereby the collective behaviours of a crowd can cause a hypnotic impact on an individual, leading to changes in behaviours | ^248^ |
| Audience effects | A type of social facilitation whereby one’s actions are influenced by the presence of others | ^249^ |
| Increased social performance | Whereby one performs better on tasks when others are around (see also social inhibition) | ^250^ |
| Increased prosocial behaviour | Behaviour that benefits others or has positive social consequences | ^251^ |
| Increased altruism | Disinterested and selfless concern for the wellbeing of others and the exchange of beneficial acts between individuals | ^252^ |
| SOCIAL RELATIONSHIPS |  |  |
| Enhancing social engagement |  |  |
| Increased positive attitude towards social exchange | Whereby we evaluate the pros and cons of social behaviour and determine it to be beneficial | ^253^ |
| Increased social networks | The web of relationships that surround an individual that can be described in terms of size, density, boundedness (degree to which they are defined based on traditional group structures such as kin, work, neighbourhood) and homogeneity | ^240^ |
| Stronger network ties | The characteristics of the relationships within social networks, including duration (the length of time one knows another person), intimacy, multiplexity (the number of types of social transactions or support involved) and reciprocity (the extent to which exchanges are mutual) | ^240^ |
| Greater satisfaction of social needs | Including the formation of friendships, romantic attachments and other emotional relationships that an individual feels are necessary to them | ^254^ |
| Reduced iso-strain | Whereby one experiences high demands, low control and low social support/isolation | ^255^ |
| Supporting social bonding |  |  |
| Increased reciprocity | Whereby one responds to the positive action of another with a positive action of one’s own | ^256^ |
| Increased emotional closeness | A perception of closeness to another that allows the sharing of personal feelings | ^257^ |
| Increased social bonding | A special form of affiliative behaviour in which selective social attachments strengthen social relationships | ^258^ |
| Improved attachment styles | Changes in the type of affectional bonds we build with others, in particular moving towards more secure attachments and away from avoidant or anxious attachments | ^259,260^ |
| Increased satisfaction of desire for attachment | The need for close and secure bonds with others, which functions as a primary motivational system | ^260^ |
| Reduced social isolation | An objective state whereby an individual has low levels of or a complete absence of social contact | ^261^ |
| Reduced loneliness | A complex and typically negative subjective emotional response to perceived deficiencies in the number of or extent of one’s social relationships | ^261^ |
| Decreased alienation | A high degree of distance or isolation or lack of common values between individuals | ^262^ |
| Increased opportunity for solitude | A positive condition in which a person is alone but not necessarily separated from others | ^263^ |
| Increased belonging | An affinity for a place, social location, group or collective, or ethical or political value system that leads to the feeling of being ‘at home’ | ^264^ |
| Achievement of biosocial goals | Such as to elicit and provide care from and to others, find a suitable mate, form cooperative alliances and reach high social rank | ^265^ |
| LEARNING AND TRAITS |  |  |
| Building social learning and traits |  |  |
| Improved social skills | The tools that enable one to communicate, learn, make friends and interact with society | ^266^ |
| Reduced othering | Whereby one views another in a negative way because of a distinguishing characteristic or trait | ^267^ |
| Reduced individualism | The prioritisation of the individual over the entire group | ^268^ |
| Enhanced civic individualism | Increasing freedom of action for individuals but in a way that acknowledges global civic culture and diversity | ^269^ |
| Increased social trust | Reliance on the character, ability, strength, or truth of others within society | ^270^ |
| Increased social responsibility | Whereby one feels an obligation to act for the benefit of society at large | ^271^ |
| Changes in framing | Schemas of interpretation that are socially constructed and which individuals rely on to understand and respond to events | ^272^ |
| Building cultural learning and traits |  |  |
| Increased cultural attachment | The formation of secure attachment to one’s native and/or host culture | ^273^ |
| Increased cultural learning | A form of social learning that allows for fidelity of transmission of behaviours and information within cultures | ^274^ |
| Increased acculturation | The process of change by which an individual adjusts to a new cultural environment, adopting and acquiring their behaviours or values (see also sociocultural adaptation) | ^275^ |
| Reduced acculturative stress | The psychological stress of integrating into a new culture | ^276^ |
| Increased inter-cultural competence | Behaving and communicating in a way that is suitable according to one's culture to achieve desired goals | ^277^ |
| Increased cultural embodiment | A means of gaining information about the world and the people within it through perception and attention facilitated by the body | ^278^ |
| SOCIAL RESOURCES |  |  |
| Building social identity |  |  |
| Changes in anonymity | Feeling anonymous or invisible (which can be both positive and negative for health) | ^279^ |
| Development of an interdependent self | The self that is dependent on and fundamentally connected to other people | ^31^ |
| Development of social identity | An individual’s sense of who they are based on their group membership (e.g. leisure club) | ^280^ |
| Development and reinforcement of social roles | The part an individual plays as a member of a social group and the corresponding changes they make to fit the expectations of that role | ^281^ |
| Validation of experiences | The acceptance of one’s own internal experiences, thoughts or feelings as a result of the recognition and acceptance of these experiences by another | ^282^ |
| Increased feeling of being valued | One’s perceptions of the respect they receive from others, either generalised respect (e.g. towards all individuals within a group or collective), or individualised respect (for particular attributes, behaviours or achievements) (see also worth) | ^283^ |
| Increased positive social comparisons | The determination of one’s social and personal worth based on how one compares oneself to others | ^284^ |
| Heightened social status | One’s perceived relative rank within society | ^285^ |
| Building capital |  |  |
| Increased social support | Instrumental, financial, informational, appraisal or emotional support provided by others | ^240^ |
| Increased social capital | Resources embedded in one's social network and social ties, including bonding social capital (links to like-minded people), bridging social capital (links to heterogeneous groups) and linking social capital (links to people in dissimilar situations or outside of one’s community) | ^90^ |
| Increased employability | The attributes of a person that make that person able to gain and maintain employment | ^90^ |
| Increased physical capital | Tangible resources such as wealth, property and assets | ^90^ |
| Increased socio-economic position | An aggregate concept that includes both resource-based and prestige-based measures (e.g. education, income and occupation), as linked to both childhood and adult social class position | ^286^ |
| Increased social conversion factors | One’s ability to use social resources (such as public policies, social norms, power relations etc) to support oneself in doing things within one’s life | ^92^ |
| Increased environmental conversion factors | One’s ability to use environmental resources (such as the physical or built environment in which a person lives, and the means of communication or transportation) to support oneself in doing things within one’s life | ^92^ |

## **4.2 Meso-/Macro-level**

| Mechanism | Definition | Reference |
| --- | --- | --- |
| GROUP STRENGTH |  |  |
| Supporting group cohesion |  |  |
| Enhanced social surroundings | Whereby individuals are surrounded by supportive others who move with them through the life course (see also convoy theory) | ^287^ |
| Increased mutual dependence | Relying upon and benefiting from a collaborative partnership or group membership | ^288^ |
| Enhanced social solidarity | The interdependence between individuals that allows individuals to feel they enhance the lives of others | ^289^ |
| Enhanced collectivism | A focus on group goals, relationships and what is best for the collective group | ^290^ |
| Increased social cohesion | The willingness of members of a society to cooperate with each other across economic divides in a way that reduces inequalities in order to survive and prosper | ^291^ |
| Increased community cohesion | The willingness of members of a society to cooperate and solve problems between different communities, based on ethnic, faith or cultural divisions | ^292^ |
| Decreased ostracism | Whereby certain individuals or groups are ignored or excluded | ^293^ |
| Increased shared achievements | The experience of accomplishing a goal or act as a group or as the result of a group effort | ^294^ |
| Building integration & resilience |  |  |
| Reduced marginalisation | Whereby certain individuals or groups are relegated to the sidelines with regards to opportunities and resources and treated as less significant | ^295^ |
| Decreased cultural homogenisation | Whereby two or more cultures are integrated in a way that damages cultural heritage | ^296^ |
| Increased normative multi-culturalism | Whereby society is culturally diverse and accepting in a way that avoids stereotyping and bias | ^297^ |
| Preservation of cultural traditions | The languages, relics, events, rituals and customs that a society shares | ^298^ |
| Increased polyculturalism | Whereby different cultural groups connect with and influence one another in a dynamic way | ^299^ |
| Improved social resilience | The capacity of groups or communities to cope with external stresses and disturbances as a result of social, political and environmental change | ^300^ |
| Improved community resilience | The ability of a community to use its available resources to respond to and recover from adverse situations | ^301^ |
| POWER |  |  |
| Disrupting hierarchies |  |  |
| Changes in social hierarchies | The system of ranking according to relative status or authority within groups or society | ^302^ |
| Reduced social dominance | Whereby group-based inequalities are maintained through processes of discrimination and behavioural inequality | ^303^ |
| Increased social mobility | The movement of individuals, families, households, or other categories of people within or between social strata in a society | ^304^ |
| Social change | Significant alterations over time in behavioural patterns, cultural values or norms | ^305^ |
| Increased community empowerment | The process of enabling communities to increase control over the factors that shape their lives, increase their assets, build their capacities, and gain control | ^306^ |
| Changes in social power | The available tools and social influence an individual or group has to exert influence over others | ^307^ |
| Increasing cultural evolution | Whereby whole population thinking leads to cultural change | ^308^ |
| Improving equality |  |  |
| Increase in social justice | Fair and just relationships between individuals and a society, as measured by the distribution of wealth, opportunities for personal activity and social privileges | ^309^ |
| Decreased relative deprivation | The lack of resources to sustain the basic lifestyle or activities that a group is accustomed to within society | ^310^ |
| Decreased area deprivation | An area's potential for health risk as a result of a combination of factors such as poverty, unemployment, social problems or economic disinvestment | ^311^ |
| Increased neighbourhood security | The safety of an area such that individuals living or working in the area do not perceive themselves to be vulnerable to risks or threat | ^312^ |
| Reduced income inequality | The disparity of income distributions within a society (which can be reduced through factors such as the provision of skills and employment to diverse social groups) | ^313^ |
| Reduced social inequality | Unequal opportunities and rewards for different social positions or statuses within a group or society | ^314^ |
| Increased health equity | The absence of avoidable or unfair differences among groups | ^315^ |

| Developing the leisure industry |  |  |
| --- | --- | --- |
| Increased leisure industrialisation | The transformation of leisure activities into global industries | ^316^ |
| Increased numbers of jobs within leisure | Opportunities for individuals to have paid work (for example within the leisure industry) | ^317^ |
| Changes in labour market structure | Changes in the supply of labour and the demand for labour as a result of factors such as economic growth and social change (which may be achieved through a growth or change in leisure engagement) | ^318^ |
| Improvements in economic stability & security | Whereby the financial system of a nation displays only minor fluctuations in output growth and exhibits a consistently low inflation rate (which may be achieved through the success of industries such as the creative industries) | ^316^ |
| Development of leisure-related public policies | The process by which governments translate their political visions into programmes and actions e.g. constitutions, legislative acts, and judicial decisions | ^319^ |

# **Behavioural Processes: in detail**

## **5.1 Micro-level**

| Mechanism | Definition | Reference |
| --- | --- | --- |
| DEVELOPMENT OF HABIT | | |
| Disrupting existing habits |  |  |
| Disruption of autopilot | A state in which subconscious brain activity regulates our behaviour automatically allowing us to carry out activities or routines instinctively without being fully conscious | ^320^ |
| Discontinuity of habits | Whereby a change in context (e.g. through taking up a leisure activity) leads to a disruption in habits and greater deliberation over actions | ^321^ |
| Disruption of script | Whereby a new activity (e.g. taking up a leisure activity) breaks the power of a previous sequence of expected behaviours | ^322^ |
| Disrupted status quo bias | One’s tendencies to stick with whatever options are the current default | ^323^ |
| Reduced psychological reactance to norms | Whereby an individual feels their personal freedoms are being restricted (e.g. through attempts to encourage them to avoid unhealthy behaviours) and therefore engages in an act of anti-conformity | ^324^ |
| Assisting in the formation of new habits |  |  |
| Operant learning | Whereby rewards or punishments for behaviours (e.g. leisure engagement) lead to an association between a particular behaviour and its consequence | ^325^ |
| Classical conditioning | Whereby neutral stimuli take on an emotional tone by being associated with other powerful stimuli, eliciting a specific response | ^326^ |
| Performing action slips | Whereby one engages in a behaviour based on associate cues without intending to do so (see also automatic behaviour) | ^327^ |
| Formation of habits | Whereby a new behaviour is performed repeatedly in similar circumstances so that cognitive associations are formed between context cues and the behaviour that trigger the behaviour | ^328^ |
| Reinforcement of behaviours | Positive responses to behaviours across other mechanisms of action lead to increases in that behaviour | ^329^ |
| Increased continuity of positive behaviours | Whereby one retains adaptive behaviours as one ages | ^330^ |
| Development of harmonious addiction | Whereby an activity becomes a favourite pastime and the subject of autonomous involvement as a ‘passion’ | ^331^ |
| BEHAVIOURAL DECISIONS |  |  |
| Influencing individual choice |  |  |
| Reduced cognitive dissonance | Whereby there is an inconsistency between our attitudes and behaviours that has to be resolved, often through changes in behaviours | ^332^ |
| Decreased dynamic inconsistency | Whereby our preferences change over time and our future selves and present self have contradictory desires | ^333^ |
| Reduced cognitive bias | A systematic pattern in one’s thinking where one deviates from norms or rationality in judgement | ^334^ |
| Reduced disconfirmation bias | Whereby individuals refute or ignore arguments that go against their prior beliefs | ^335^ |
| Increased considerations underlying choice | Whereby decisions are made through comparing the characteristics of the options under consideration (see also multi-attribute utility theory) | ^336^ |
| Increased balancing of outcome expectancies | Whereby one acknowledges both the positive and negative outcomes of engaging in specific behaviours | ^337^ |
| Increased reasoned action | Whereby one’s decision to engage in a particular behaviour is based on pre-existing attitudes and behavioural intentions | ^338^ |
| Increased controlled risk-taking | The action of engaging in risky behaviours but in a limited risk setting (such as trying new experiences or taking on new challenges in life that are unlikely to cause harm) | ^339^ |
| Changes in perception of boundaries | The perceived dividing lines between different thoughts, feelings or current or prospective experiences | ^340^ |
| Supporting the prediction of behaviours |  |  |
| Changes in default effects | Whereby one engages in a behaviour because it is presented as the default option | ^341^ |
| Changes in deadline effects | Whereby the setting of deadlines (e.g. through having leisure activity goals) affects procrastination and choice behaviours (either promoting or reducing these behaviours) | ^342^ |
| Changes in norm effects | Whereby social norms and personal norms guide one’s actions. In particular injunctive social norms (the perception of how others approve/disapprove of one’s conduct) can guide behavioural change (see also focus theory of normative conduct) | ^343^ |
| Changes in self-fulfilling prophecies | Whereby predictions or expectations come true because an individual believes it will and aligns their behaviour to fulfil those beliefs | ^344^ |
| Changes in behavioural prediction | Whereby one’s behaviour follows reasonably from their beliefs | ^345^ |
| Increased response inhibition | Suppression of actions that are inappropriate in a given context and interfere with goal-driven behaviour | ^346^ |
| Enhanced inhibitory control | Whereby an individual inhibits their impulses and dominant behavioural responses to a stimuli in order to select a more appropriate behaviour | ^347^ |
| BEHAVIOURAL DRIVE |  |  |
| Enhancing behavioural activation |  |  |
| Unfreezing of behaviours | Whereby one becomes aware of problems with the current situation and accepts the need to make change (see also change theory) | ^348^ |
| Increased initiative | One’s ability to independently assess and carry out appropriate actions in a given situation | ^349^ |
| Increased goal setting | As a result of mechanisms relating to motivation, attitudes and subjective norms (see also goal-setting theory) | ^350^ |
| Increased self-activation | Whereby behaviours are activated when values that are seen as a core part of one’s self-concept are cognitively activated (e.g. through leisure engagement) | ^321^ |
| Increased self-efficacy | An individual’s belief in their ability to succeed in specific situations or accomplish tasks | ^351^ |
| Increased readiness to act | Whereby an individual enters pre-contemplation or contemplation stages that can lead to preparation, action and maintenance of behaviours (see also transtheoretical model) | ^352^ |
| Increased mental simulation | Our mind's ability to imagine taking a specific action and simulating what the probable result will be before acting | ^353^ |
| Increased action tendency | The urge to carry out certain behaviours linked to specific emotions | ^354^ |
| Increased self-determination | One’s free choice of one’s future actions | ^355^ |
| Decreased ego depletion | Whereby energy for mental activity is limited and if depleted leads to low self-control. Ego depletion can be restored through short-term good mood and long-term building of inner resources and self-regulation | ^356^ |
| Decreased self-sabotaging beliefs | Thoughts that create problems in daily life or interfere with goals such as procrastination, self-injury or negative internal dialogue (see also goal sabotaging) | ^357^ |
| Increasing motivation |  |  |
| Decreased apathy | A lack of interest, enthusiasm or concern that may be decreased through social connection or behavioural change | ^358^ |
| Increased extrinsic motivation | A desire to engage in activities or behaviours that either reduce biological needs or help us to obtain incentives or external rewards (see also drive theory) | ^359^ ^360^ |
| Increased intrinsic motivation | A desire to engage in activities or behaviours because they are personally rewarding and fulfil our expectations and beliefs | ^359^ |
| Increased expectancy motivation | Whereby the expectation of positive responses increases engagement | ^361^ |
| Increased task-based motivation | Whereby core characteristics of a task are associated with task satisfaction, motivation and engagement (see also job characteristics model) | ^362^ |
| Increased identity-based motivation | Whereby one’s identity and self-concept motivates one to take action towards one’s goals | ^363^ |
| Reduced fear of failure | Motivation to avoid failure by not doing tasks that are too challenging and finding ways to ensure one does not succeed (see also self-handicapping) | ^364^ |
| Anticipated regret from non-engagement | Whereby the anticipated regret of not engaging leads one to engage further in a behaviour (see also fear of missing out) | ^365^ |
| Increased evaluation apprehension | Whereby we fear the evaluation of others, which motivates behaviour | ^366^ |
| Increased implementation intentions | A self-regulatory strategy whereby an individual plans how and when to engage in a behaviour in order to achieve a specific goal | ^367^ |
| Perception of being given a second chance | An opportunity to try something again after failing | ^368^ |
| Supporting achievement |  |  |
| Increased sense of reward | Satisfaction or a feeling of profit that results from factors such as learning, emotion and motivation | ^369^ |
| Increased commitment | As a result of an individual making choices that make it more costly for them to make unwanted choices in the future (e.g. signing up to a group performance or paying up front for annual gym membership) | ^370^ |
| Increased perseverance | Persistence in doing something despite difficulty or delay in achieving success | ^371^ |
| Increased goal attainment | As a result of the achievement of one goal (e.g. relating to leisure engagement) leading to the setting and achievement of further goals (see also goal-setting theory) | ^350^ |
| Increased experience of making mistakes | Errors or slip-ups, that can in turn lead to processes of improvement and learning | ^372^ |
| Improved personal competence | An ever-evolving accumulation of related capabilities that facilitate learning and other forms of goal attainment | ^373^ |
| Increased self-actualisation | The realisation or fulfilment of one’s talents and potentials | ^254^ |
| Increased generativity | Making one’s mark on the world through creating or nurturing things that will outlast oneself | ^374^ |
| BEHAVIOURAL DEVELOPMENT |  |  |
| Supporting child development |  |  |
| Improved infant behaviours | Such sleeping, suckling, feeding, cuddling and crying and responses to behavioural cues and interaction | ^375^ |
| Increased social referencing | Whereby a child regulates their behaviour towards environmental objects, people or situations | ^376^ |
| Enhanced school readiness | The academic, attention, and socioemotional skills required for a child to enter school ready to engage in and benefit from early learning experiences | ^377^ |
| Reduced truancy | Absenteeism from school without good reason | ^378^ |
| Improved parenting practices | Behavioural approaches of parents towards children, including monitoring behaviours (parents’ awareness of their children’s actions), nurturance behaviours (the degree to which parents are supportive of their children), and the consistency of how parents address their children’s inappropriate behaviours | ^379^ |
| Increased play | Engagement in activities for enjoyment and recreation rather than a serious or practical purpose (relevant both to children and adults) | ^380^ |
| Supporting behavioural adjustment |  |  |
| Reduced externalising behaviours | Behavioural problems manifested in outward behaviours such as disruptive, hyperactive, aggressive or anti-social behaviours | ^381^ |
| Reduced internalising behaviours | Behavioural problems that more centrally affect an individual’s internal psychological environment including anxiety, withdrawal, inhibition, and over-controlled behaviours | ^381^ |
| Improved development of childhood adaptive behaviours | Such as developing social skills, personal responsibility, cognitive skills and learning how to carry out essential activities of daily living. They can occur as a result of supportive social and environmental factors during child development (ecobiodevelopmental model) | ^382^ |
| Reduced bullying | Repeated oppression, psychological or physical, of another | ^383^ |
| Increased independence | A personality trait in which a person is comfortable and confident acting on his/her own thoughts and feelings | ^384^ |
| PERSONAL LOCATION |  |  |
| Enhancing a sense of time |  |  |
| Increased flow | A state in which one becomes absorbed in an activity such that nothing else seems to matter | ^385^ |
| Increased sense of momentum | Impetus and driving force gained through an action or course of events | ^386^ |
| Decreased boredom | An emotional state experienced when one has nothing in particular to do | ^358^ |
| Provision of routine | A regular schedule of actions and events | ^387^ |
| Displacement of time to engage in unhealthy behaviours | Whereby one has less time available to engage in unhealthy behaviours due to less spare time | ^388^ |
| Changes in perception of time | Whereby an individual re-orientates their sense of time for the past, present and/or future | ^389^ |
| Reduced present bias | Whereby one places disproportionate weight on present rather than future concerns (see also hyperbolic discounting) | ^390^ |
| Increased anticipation of forthcoming positive events | Pleasure in considering or waiting for expected events (including leisure events) |  |
| Increased prospection | The act of anticipating, thinking forwards or simulating future events (see also futures thinking) | ^391^ |
| Enhancing a sense of place |  |  |
| Enhanced sense of place | A social phenomenon whereby one’s sense of personal and cultural identity is bound up with place identity | ^392^ |
| Development of a safe space | A place or environment in which an individual feels confident they will not be exposed to any physical or emotional harm, including discrimination, criticism or harassment | ^393^ |
| Increased perceptions of safety | Perceptions of being both protected from harm and unlikely to cause harm to others | ^394^ |
| Enhanced attachment to place | The affective bond between people and place or setting (see also field of care) | ^395^ |
| Changes in place-based behaviour | Whereby a particular place (the behaviour setting e.g. a museum) provides inputs and controls that predict human behaviour in that place | ^396^ |
| Increased local engagement | Whereby engaging in a leisure activity increases the likelihood one will engage in other activities close by as the cost and effort required for these further activities is lower (see also friction of distance) | ^397^ |
| Expanded life-world | The spatio-temporal setting or horizon of one’s everyday life as defined in cultural terms | ^398^ |

## **5.2 Meso-/Macro-level**

| Mechanism | Definition | Reference |
| --- | --- | --- |
| COOPERATION |  |  |
| Increasing social control |  |  |
| Changes in patterns of herd behaviour | The tendency of large groups to conform to group choices (see also informational social influence) | ^399^ |
| Enactment of ritual | Whereby groups perform a series of actions according to a prescribed order (e.g. when engaging in a leisure activity) | ^400^ |
| Changes in social shaping | The modulation of an individual’s psychological, biological, social, and behavioural responses to events and circumstances through one’s social relationships | ^401^ |
| Changes in social regulation of effort | Whereby one’s adaptation to the social environment leads to an expectation of the amount of energy that will be required for tasks, such that changes in the resources available lead to increases in required effort and consequent distress (see also social baseline theory) | ^402^ |
| Changes in levels of territoriality | How people use space (territory) to communicate occupancy or ownership of areas or assets | ^403^ |
| Changes in informed social control | Whereby the reactions of individuals and groups lead to conformity to norms and laws (see also social surveillance) | ^404^ |
| Reduced social strain | Whereby social structures, such as area deprivation, may pressure citizens to commit crimes | ^405^ |
| Building group learning |  |  |
| Increased social learning | Whereby groups learn behaviours (within and across generations) by observing, imitating and modelling the actions of others | ^406,407^ |
| Reduced bandwagon effect | Whereby the rate of uptake of beliefs, ideas, fads and trends increases the more that these things have already been adopted by others, without individual conscious thought | ^408^ |
| Increased cooperation | The action or process of individuals working together to maximise outcomes for self and others | ^409^ |
| Increased cultural consonance | The degree to which individuals are able to follow behaviours encoded in shared cultural models | ^410^ |
| Increased collective intentionality | The power of minds in a group to be jointly directed at objects, goals, or values through joint attention, shared intention, shared belief and collective acceptance | ^411^ |
| Ecological transition | The shift of roles that occurs over life course, leading to change in a person's behaviour but also affecting the wider environment they interact with | ^412^ |
| Encouraging adaptive group behaviours |  |  |
| Engagement in collective projects | Whereby individuals work together to achieve something (see also supra-individual projects) | ^101^ |
| Increased active citizenship | The involvement of individuals in their local communities and democracy at all levels | ^413^ |
| Increased collective action | Action taken together by a group with the aim of enhancing their status or achieving a common objective | ^414^ |
| Development of social movements | A type of group action that focuses on specific social or political issues and empowers oppressed groups to mount effective challenges to or resist the status quo (see also social movement theory) | ^415^ |
| Increased political participation | The engagement of individuals in activities that develop and express their opinions on the world and how it is governed and try to shape these decisions | ^416^ |
| Increased sharing of resources | The process of dividing and distributing money, materials or other tangible and intangible assets between individuals and groups (see also distributive justice) | ^417^ |
| Changes in civil unrest | Fighting between different groups of people such as between groups of citizens and their authorities (see also civil disorder or disturbance) | ^418^ |
| Decreased conflict | Active disagreement or fighting between opposing groups | ^419^ |
| Reduced criminality | Behaviour that is contrary to or forbidden by criminal law | ^420^ |
| APPROACHES TO HEALTH |  |  |
| Increasing social responsibility |  |  |
| Altered social conception of illness | Changing perception of health conditions from “disease” (something that needs to be cured and is the health professional’s concern) to “illness” (something that needs to be managed and is an innately human experience of symptoms and suffering) | ^48^ |
| Increased assumption of social responsibility | Whereby the development of new roles within society (e.g. as a result of leisure activities) places pressure on individuals to take on responsibilities (see also staffing theory or manning theory) | ^421^ |
| Increased collective responsibility in health | Whereby cooperation between individuals and health professionals leads to greater group engagement in prevention and treatment of illness | ^422^ |
| Increased social accountability towards health | Actions initiated by citizen groups to hold authorities (e.g. public health officials, politicians, and health providers etc) to account (e.g. for delivering health services and improving public health) | ^423^ |
| Increasing health promotion |  |  |
| Increased collective self-efficacy | A group’s beliefs in its ability to carry out actions successfully | ^424^ |
| Increased structuralist-collectivist health promotion | Health promotion activities that focus on involving the community with health programmes and developing health legislation | ^425^ |
| Improved social-ecological health promotion | Approaches to health promotion that recognise that individuals are embedded within larger social systems an individual, interpersonal, organisational, community and policy level | ^426^ |
| Changes in adaptation behaviours | The strategies adopted at a group level such as planning, budgeting and decision-making to tackle health problems | ^427^ |
| AVAILABILITY OF ASSETS |  |  |
| Increasing leisure assets |  |  |
| Increased number of leisure artefacts | The tangible products produced as a result of leisure engagement e.g. artworks, books, performances, gardens and monuments | ^428^ |
| Increased diversity of leisure artefacts | The extent to which leisure artefacts represent and have a relevance to a wide range of groups, cultures and societies | ^428^ |
| Increased number of leisure experiences | The intangible products produced as a result of leisure engagement e.g. performances, festivals, events and stories | ^428^ |
| Increased diversity of leisure experiences | The extent to which leisure experiences represent and have a relevance to a wide range of groups, cultures and societies | ^428^ |
| Increased number of community assets | The number of buildings, monuments or other land of community value e.g. parks, gardens, leisure centres, cultural venues, libraries and other recreational spaces | ^429^ |
| Increased diversity of community assets | The extent to which community assets represent and have a relevance to a wide range of groups, cultures and societies | ^429^ |
| Increased geographical spread of community assets | The distribution of community assets across countries, geographical regions, areas of urban or rural classification, and areas of varying deprivation | ^429^ |
| Increased preservation of community assets | The protection and safeguarding of cultural, heritage and natural sites | ^430^ |
| Increased placemaking | The development of public spaces in way that capitalises on local community assets with the aim of promoting individuals’ health and wellbeing | ^431^ |
| Increasing healthcare assets |  |  |
| Increased numbers of paraprofessionals | Non-medical professionals who support the delivery of healthcare (including arts therapists, social prescribing link workers, and leisure staff running community health programmes) | ^432^ |
| Increased availability of self-help groups | Groups of people who share a common problem (e.g. illness or addiction) and provide mutual support for each other (see also mutual help, mutual aid, or support groups) | ^433^ |
| Increased availability of leisure-based activities to support health | Leisure activities specifically designed or provided in order to support aspects of mental or physical health | ^434^ |
| Increased referral systems to leisure activities | Such as social prescribing schemes that support the referral of individuals from health and social care professionals to leisure programmes | ^435^ |

# **Health Behaviours: in detail**

## **6.1 Micro-level**

| Mechanism | Definition | Reference |
| --- | --- | --- |
| ENGAGEMENT IN HEALTHY BEHAVIOURS |  |  |
| Increasing behaviours relating to the prevention of ill-health |  |  |
| Increased leisure engagement | Engagement in leisure activities. This can result from mechanisms including learning to engage in leisure activities (see also leisure socialisation) | ^436^ |
| Reduced sedentary behaviours | Behaviours that involve sitting or lying down | ^437^ |
| Increased physical activity | Whereby individuals become more active, increasing behaviours that involve moderate or vigorous exercise or activity | ^437^ |
| Improved sleep | A condition of body and mind in which the nervous system rests and consciousness is largely suspended | ^438^ |
| Increased engagement with behavioural immunogens | Behavioural immunogens (see also health promoting behaviours) include healthy eating, exercise and attending health checks. They can be increased as a result of combinations of mechanisms relating to coping and the perception of the threat (see also protection motivation), combinations of mechanisms relating to perceived risk, benefits, motivation and psychological factors leading to change in health beliefs (see also health belief model), or combinations of mechanisms relating to self-efficacy, goal setting, risk perception and outcome expectancies (see also Health Action Process Approach) | ^439–441^ ^442^ |
| Repeated healthy behaviours | Health-promoting behaviours can become regular as a result of mechanisms involving social bonding, others’ behaviour and attitudes, cultural knowledge and values, a sense of self and social competence leading to motivational beliefs, attitudes, self-efficacy and trials of behaviour (see also triadic influence model) | ^443^ |
| Increased responsiveness to health communication | The degree to which one reacts quickly and positively to health messages. This can be enhanced through combinations of mechanisms relating to motivation, cognitive processing, personal beliefs and comprehension (see also elaboration-likelihood model) | ^444^ |
| Increasing behaviours relating to the management of ill-health |  |  |
| Increased self-management of health | The ability of an individual to look after their own health. This can be enhanced through combinations of mechanisms relating to knowledge and beliefs, self-regulation skill and ability and social facilitation (see also integrated theory of health behaviour change) | ^445^ |
| Increased treatment adherence | The degree to which an individual correctly follows medical advice (see also compliance) | ^446^ |
| Reduced need for medical treatment | Reduced experience of symptoms or health conditions that require medication or other medical intervention, or the better tolerance or management of such symptoms or conditions | ^446^ |
| Avoidance of relapse | A deterioration in one’s mental or physical health after a period of improvement. This can be protected against through combinations of mechanisms relating to coping and self-efficacy (see also Marlatt’s cognitive behavioural model) | ^447^ |
| Changes in use of health services | Use of healthcare services or spaces can be increased through such engagement becoming the default option e.g. via co-location of leisure activities within healthcare settings (see also nudge theory), or via individuals taking more responsibility for their own health, or could be decreased as a result of healthcare problems being addressed | ^98^ |
| Increased self-protection behaviours | Behaviours that an individual engages in to protect their health. These can be encouraged through combinations of mechanisms relating to planning and implementation intentions (see also precaution adoption process) | ^448^ |
| Provision of informal care | Unpaid care provided to older or dependent individuals by individuals with whom the individual has a social relationship (e.g. family or friends) | ^449^ |
| Developing behaviours to support the delivery of healthcare |  |  |
| Improved clinical skills | Procedural knowledge, science knowledge and clinical reasoning that enable the development and application of physical examination skills, communication skills, management skills or the execution of practical procedures | ^450^ |
| Improved staff-patient interactions | The quantity (i.e. frequency of contact) and quality (e.g. clarity of communication and compassion) of engagement between healthcare professionals and patients | ^451^ |
| Reduced clinical errors | The failure of a planned action within healthcare to be completed as intended or the use of a wrong plan to achieve an aim | ^452^ |
| Reduced staff burnout | A syndrome that results from chronic workplace stress that has not been successfully managed, involving feelings such as exhaustion, negativity, cynicism, psychological distance from one’s work, and reduced job performance | ^453^ |
| DISENGAGEMENT IN UNHEALTHY BEHAVIOURS |  |  |
| Reducing engagement in unhealthy activities |  |  |
| Decreased unhealthy behaviours | Engagement in behaviours that are detrimental to health such as poor diet or lack of exercise. These behaviours can be triggered by a perceived dissonance between one’s attitudes and one’s behaviours (see also post-decisional conflict) or due to attempts to convince oneself that our behaviours are adequate and to ignore behaviours that do not fit our sense of self (see also compensatory health beliefs) | ^454,455^ |
| Decreased problem behaviours | Problems such as anger and aggression. These can be reduced through combinations of mechanisms relating to values, control, social support and socialisation (see also problem behaviour theory) | ^456^ |
| Decreased delinquent or deviant behaviour | Behaviours that violate social norms such as crime or violence. These can be reduced through combinations of mechanisms relating to socialisation, skills and beliefs (see also social development model), or mechanisms relating to negative self-attitudes (see also general theory of deviant behaviour) | ^457,458^ |
| Decreased unsafe sex practices | This includes engaging in sex without protection or putting an individual at risk of sexually transmitted diseases. Unsafe sex can be increased as a result of the normalisation of aggressive behaviour and the alteration of norms governing behaviour (see also disinhibition theory) | ^459^ |
| Decreased screen time | The amount of time spent using devices with a screen such as computers, televisions, smartphones, or games consoles | ^460^ |
| Reducing engagement with substances |  |  |
| Decreased smoking and tobacco use | Including the smoking of cigarettes, pipes, cigars and e-cigarettes. It can be reduced through combinations of mechanisms relating to status, self-image, personality, physiological reactions and social engagement | ^461^ |
| Decreased drug use | The use of illegal substances or overuse of prescription medication. Drug use can be increased as a result of negative self-attitudes (see also general theory of deviant behaviour) | ^458^ |
| Improved management of addiction | The need for a particular substance or activity. It can be reduced as a result of increased control over negative aspects of withdrawal via emotional mechanisms (see also opponent process theory) | ^462^ |

## **6.2 Meso-/macro-level**

| Mechanism | Definition | Reference |
| --- | --- | --- |
| HEALTHCARE |  |  |
| Influencing healthcare delivery |  |  |
| Enhanced therapeutic landscapes | The combination of physical, social and human factors within environments to produce an atmosphere that is conducive to health and healing | ^463^ |
| Increased de-institutionalisation | Whereby individuals are cared for in the community rather than in institutions (e.g. hospitals) as much as possible | ^464^ |
| Increased primordial prevention | The prevention of risk factors through changes in social and environmental conditions with a focus on childhood | ^465^ |
| Improved health communication | The practice of communicating health information e.g. through public health campaigns, health education, and doctor-patient interactions | ^466^ |
| Increased availability of person-centred care | Whereby people have choice and control over the way their care is planned and delivered (see also personalised care) | ^467^ |
| Reduced demand for healthcare services | The need and desire for health-related goods or services that result from the desire of individuals to have good health | ^468^ |
| Improving healthcare performance |  |  |
| Increased recognition of paraprofessionals | The appreciation of non-medical professionals supporting healthcare (including arts therapists, social prescribing link workers, and leisure staff running community health programmes) | ^432^ |
| Disruption of healthcare societies | The rules of behaviour, tradition, language, hierarchies and priorities that can be detrimental to individual or humanised care provision | ^469^ |
| Rehumanisation of health | Whereby healthcare is reconnected with individuals and rewards are based on caring for patients above meeting targets (see also depersonalisation) | ^470^ |
| Development of more compassionate care environments | Whereby care is designed and delivered in a way that seeks to minimise the sufferings or misfortunes of others | ^470^ |
| Enhanced performance & quality of healthcare | Achievement of targets and delivery of high quality care | ^471^ |
| Enhanced mutual recovery | A more fully social understanding of recovery processes, encompassing groups including patients, informal carers, healthcare professionals and paraprofessionals | ^472^ |
| Removing barriers to healthcare |  |  |
| Reduced health discrimination | The unjust or prejudicial treatment of different groups such as those defined by race, age, disability, religion, sex, gender, illness etc | ^113^ |
| Increased engagement with hard-to-reach groups | Contact and collaborative work with minority populations or those with specialist needs who may find mainstream assistance or healthcare inappropriate or difficult to access | ^473^ |
| Increased conversations about health | Discussions between individuals or groups about symptoms, experiences or actions relating to health | ^474^ |
| Decreased accumulation of health hazards | Exposure to occupational hazards (e.g. pollution, chemicals, ergonomic strain, noise) and social hazards (discrimination, harassment, abuse). Health hazards tends to occur more frequently amongst groups with limited power and resources | ^475^ |
| Decreased clustering of negative health behaviours | The patterning of negative health behaviours as a result of choices made available to individuals being patterned based on factors such as class, gender, and cultural background (see also health lifestyle theory) | ^476^ |
| Reduced health inequities | Unfair differences in health status or the distribution of health determinants arising from avoidable factors such as poor governance, corruption or cultural exclusion | ^313^ |
| Reduced health inequalities | The uneven distribution of health or health resources as a result of a lack of resources or genetic factors | ^313^ |

# **Literature linking leisure, mechanisms, and health**

| Mechanism | Literature linking these mechanisms to leisure | Literature linking these mechanisms to health outcomes |
| --- | --- | --- |
| Affective States | Mechanisms relating to affective responses have been the subject of an extensive body of research. For example, interventional and observational studies have found changes and associations with various leisure activities in regards to improved mood, affect, and emotion regulation strategies across different population^477–481^. These findings have been shown in a number of randomised controlled trials showing increases in positive emotions and reductions in negative emotions ^482^, and meta-analyses incorporating different study designs showing a relationship between physical activity and positive affect ^483–485^. There is also an extensive body of evidence on leisure activities in regards to psychological stress and anxiety across different groups, including meta-analyses showing reduction in psychological stress and anxiety via music exposure (including music therapy) ^486,487^, and reductions in anxiety following physical activity ^488^. | Affective states have been linked directly to mental health and physical health as well as indirectly via other mechanisms such as developing a group self, modifying group emotions, increasing brain activation, changing hormone levels, building capital, and enhancing behavioural activation ^6,489–491^. |
| Resilience | Numerous studies have investigated leisure activities in regards to resilience mechanisms. For example, intervention studies have shown increases in resilience after leisure engagement ^492–494^, observational studies have also shown associations between leisure activities and resilience ^495–498^, and intervention studies have shown that leisure activities can improve coping ^499^, although the strongest evidence specifically utilises therapy models in those facing illness ^500,501^. Numerous studies have also shown how leisure activities contribute to psychological strength, such as vitality ^502^, with a particularly large body of intervention studies focusing on how leisure-time physical activity increases vitality ^503–506^. | Resilience has been linked directly to mental health and physical health as well as indirectly via other mechanisms such as eliciting affective responses, modifying brain biomarkers, altering immune function, increasing social contact, increasing motivation, and increasing behaviours relating to the prevention of ill health ^507–511^. |
| Sense Of Self | A large number of studies have investigated leisure engagement in regards to mechanisms of sense of self. For example, mixed-methods systematic reviews have highlighted associations between leisure activities (including creative therapies) and improved self-expression and self-esteem ^512–514^. Intervention studies have also shown that different leisure activities can improve sense of identity and self-acceptance, with physical activity improving self-concept and self-esteem ^515,516^, and therapeutic creative activities enhancing self-esteem ^517,518^. Meta-analyses have found the strongest evidence for leisure-time physical activity in improving self-worth and self-concept ^519–521^. | Sense of self is linked both directly and indirectly to health, for example via mechanisms such as increasing brain activation, building social learning and traits, building social identity, increasing social responsibility, and building group learning ^522–524^. |
| Personal Transformation | A number of studies have looked at how leisure activities can affect personal transformation. For example, a large number of mixed-method studies have investigated how engagement with leisure activities can be used to understand experiences of one’s own narrative and identity, particularly surrounding perceptions of health and illness via creative engagement and literature ^525–530^. A smaller body of intervention studies have shown that leisure-time physical activity increases personal growth ^531^ and improves self-regulation ^532^. | Personal transformation is directly associated with mental and physical health as well as indirectly via other mechanisms such as supporting cognitive and emotional learning, building social learning and traits, influencing individual choice, and increasing behaviours relating to the management of ill health ^533–536^. |
| Flourishing | There is a large body of literature on how leisure activities can enable flourishing. Human flourishing encompasses aspects of meaning, purpose and life satisfaction ^537^, so whilst complex to measure, a number of conceptual studies have explored this relationship ^538,539^. Observational studies have shown associations between leisure engagement and higher ratings of life being worthwhile ^540^. Additionally, mixed-design evidence syntheses have highlighted how leisure activities promote meaning-making and purpose ^541,542^, with these findings echoed by conceptual studies ^543^ and multicultural perspectives ^544^. Systematic reviews of observational studies have also shown associations between different leisure activities and life-satisfaction ^545,546^, mirrored by intervention studies also showing improvements ^547^. | Flourishing is linked with health directly and via a range of other mechanisms such as aiding personal evolution, altering immune function, modulating cardiovascular factors, building capital, enhancing behavioural activation, supporting achievement and reducing engagement in unhealthy activities ^548–552^. |
| Psychological Capabilities | A large body of evidence has investigation at how leisure activities are related to mechanisms of psychological capabilities, with substantial research related to improved cognition and cognitive processes. For example, a number of observational studies have looked at how leisure engagement is associated with reduced cognitive decline and the maintenance of cognitive reserve ^553–556^, which is supported in part by a number of meta-analyses, particularly for physical activity ^557–559^. Additionally, there is also some research for leisure activities supporting mechanisms such as divergent thinking ^560–562^. | Enhanced psychological capabilities are associated directly with health as well as indirectly via mechanisms such as developing autonomy, activating perceptual processes, improving physical function, catalysing social actions, supporting social bonding, building group learning, and improving healthcare delivery ^563–567^. |
| Psychological Resources | There is a large body of research on how leisure engagement can build psychological resources. For example, a particularly well-evidenced area is health literacy and understanding of health via engagement with leisure-based activities, supported by an array of mixed-methods intervention studies ^568–573^. As further examples, meta-analyses have explored more specific activities and types of health literature, such as studies showing a relationship between storyline engagement and sexual health literacy ^574^. There is also an extensive body of literature that has explored how leisure participation is embedded within and contributes to different forms of capital ^575–579^. | Psychological resources are directly linked to health outcomes as well as being linked indirectly through effects on other mechanisms such as building identity, reducing load, disrupting hierarchies, building capital, increasing health promotion, increasing healthcare assets and developing behaviours to support the delivery of healthcare ^580,581^. |
| Group Mind | Overall, group-level psychological processes have received less attention than many of the individual-level processes discussed. However, a number of studies have researched leisure engagement in relation to mechanisms of group mind. For example, qualitative analyses have reported that group leisure activities can build collective sense, through themes such as shared responsibility ^582^, fieldwork reflections have highlighted the interplay between leisure and collective consciousness in respect to ethnic minority groups ^583^, and an observational study has reported that cultural consonance related to leisure activities is an important aspect of leisure satisfaction ^584^. Research has also explored sense-making via leisure engagement through interactions with one’s environment ^585,586^. | Group mind is associated with other mechanisms such as supporting changing identity, building general resources, supporting social cohesion, encouraging adaptive group behaviours, and improving healthcare performance ^102,587–589^. |
| Group Attitudes | Numerous studies have explored leisure activities in relation to mechanisms of group attitudes. For example, a number of intervention studies have shown how leisure-based activities can help to reduce stigma and negative stereotypes within different contexts ^530,590–593^. There is also a number of intervention studies that have looked at how leisure-based activities used within clinical settings can contribute to increased satisfaction with healthcare ^594–598^. | Group attitudes can affect health through mechanisms such as changing hormone levels, modulating brain biomarkers, supporting social bonding, improving equality, increasing social responsibility, and removing barriers to healthcare ^599–602^. |
| Language | There is a large body of literature on leisure activities in regards to mechanisms of group communication and group emotions. Much of the work in this space is theoretical and conceptual, based on observations. For example, studies have discussed the role of music and the arts in building collective effervescence ^603,604^ and community resilience ^605^. Observational studies have reported how music is associated with the formation of collective memories ^606^, how attending music festivals is related to the building of collective emotion and collective effervescence ^607^, and how physical activity can support collective effervescence ^608^. But there are also some preliminary intervention studies, such as showing how leisure activities can help in collective recovery from trauma ^609^. | Language has been shown to affect health through activating mechanisms including supporting emotional learning, eliciting affective responses, supporting emotion regulation, supporting social bonding, building social identity, and supporting achievement ^610–612^. |
| Nervous System | There is a large body of experimental research demonstrating associations between leisure activity interventions such as exercise ^613^ mindfulness ^614^ and music ^615^ and changes to neurophysiology, and many observational analyses have further found associations between creativity and neurophysiology ^613–619^. Diverse nervous system mechanisms activated by music ^620,621^, arts ^622^, socialising ^623^, exercise ^624,625^, dance ^618^ and spirituality have been summarised in a range of reviews and theory articles ^626,627^. | Activities within the nervous system have been linked either directly to health or indirectly to other mechanisms such as eliciting affective responses, improved sensory perception, supporting cognitive learning and cognitive processes, influencing individual choice, increasing motivation, and modulating cardiovascular and metabolic factors ^171,623,628,629^. |
| Endocrine & Immune Systems | There is a substantial literature reporting links between leisure-related interventions such as singing or concert attendance ^630–632^, art making ^633^ and competitive games ^634^ and changes to stress hormones. In addition, multiple leisure activities have been shown in intervention studies to affect cytokine responses ^635,636^ and social leisure activities have been associated in observation studies with changes to immune gene expression ^637^. These findings have been summarised in a number of review publications ^622,624,627,638,639^. | Endocrine and immune responses have been linked to mental and physical health directly as well as via wider mechanisms such as eliciting affective responses, enhancing social engagement, enhancing behavioural activation and increasing behaviours relating to the prevention and management of ill health e.g. ^640–646^. |
| Cardiometabolic System | Leisure activity interventions have been shown to affect cardiometabolic factors, such as improved cardiac output ^647,648^, vagal tone ^649,650^ and reduced arterial stiffness ^651^. These findings are supported by a number of longitudinal observational studies ^652–654^. In addition, some reviews articles have synthesised evidence between features of leisure such as socialisation ^655^, music ^656^ or spirituality ^627^ and improved cardiometabolic health. Improved cardiometabolic outcomes have been additionally summarised in systematic reviews of arts interventions ^638^. | Cardiometabolic factors have been shown to affect health directly and via other mechanisms including supporting emotion regulation, building social identity, enhancing behavioural activation, and reducing engagement in unhealthy behaviours and via body composition and associated physical and psychosocial health ^171,657–664^. |
| Performance | A small number of randomised control trials have found links between leisure interventions and physical performance, especially in older people ^665,666^. These are supported by a number of systematic reviews of mixed observational and interventional evidence for leisure activities such as music ^667^, singing ^668^ and physical activity ^669^ across all age groups. In addition, a number of systematic reviews of leisure interventions have been conducted for outcomes such as frailty ^670^ and general physical performance ^671,672^. Volunteering interventions have also been reported to improve performance in older adults ^673,674^. | Physical performance is often considered a measure of physical health outcomes as well as being linked with a range of health conditions ^638,667,675,676^. Further, physical performance is related to other mechanisms including enhancing meaning in life, building psychological strength, supporting cognitive processes, building social identity, improving equality, supporting the prediction of behaviours, and increasing social contact ^677–680^. |
| Multi-System | Whilst intervention studies are limited, several cross-sectional observational studies have identified associations between multisystem biological responses and leisure. This includes studies on epigenetic systems such as histone modification and leisure ^681^, and telomere length and leisure time physical activity ^682–684^, meditation ^685^ and green space exposure ^686^. Longitudinal studies have further identified observational associations between improved allostatic load and leisure time physical activity ^687,688^ and socialising ^689^. | Multi-system responses have been associated with a range of health outcomes and other mechanisms including through supporting coping, altering immune function, improving physical function, altering generational transmission, encouraging adaptive group behaviours, and reducing engagement in unhealthy activities ^213,215,220,690–693^. |
| Environmental Diversity | Whilst much of the research in this area focuses on the health benefits of green spaces instead of links between leisure and environmental biodiversity, a few case studies have observed improved community level biodiversity from both public and private horticulture interventions ^694,695^ and some articles have reviewed theories and observational evidence on how leisure may be associated with local and global environmental diversity ^696–698^. In addition, many leisure activities occur outdoors, so a range of observational analyses have found associations between leisure activities, such as cycling and walking, and increased exposure to nature and green spaces ^699–701^. | A broad literature of review articles ^702–708^ and number of intervention studies ^709–711^ have linked exposure to green spaces or improved biodiversity to physical and mental health directly, as well as via mechanisms such as improved health behaviours ^698,712–714^ and social capital within neighbourhoods ^696,703^. |
| Disease Susceptibility | Observational analyses have found longitudinal associations between leisure time physical activity and non-communicable diseases ^715,716,717^, disease clusters ^718^, and mortality ^719,720^. In addition, much theoretical work has framed arts and cultural activities as evolutionarily advantageous ^721–723^. However, this area of research remains less well developed than for many of the other mechanisms. | There is a direct link between disease patterns, disease transmission and health, as well as indirect links via mechanisms such as changing hormone levels, altering immune function, modulating cardiovascular and metabolic factors, changing exposure, affecting genetic and epigenetic factors, and changing behaviours relating to the prevention and management of ill health ^724–728^. |
| Social Activity | Observational studies have found associations between social activity outcomes in young people, such as reduced antisocial behaviours ^729^, crime ^730^ and improved social and academic engagement ^731^ and leisure activities. Other observational studies have identified associations between volunteering and pro-social attitudes ^732^ and between art-making and civic engagement ^733^ and pro-sociality ^734^. A number of papers have reviewed theory and observational evidence for leisure and social activity ^735–738^. Although interventional evidence is limited, exposure to pro-social song lyrics has been shown to promote sociality ^739^ and leisure interventions have been linked to social activity in children with autism ^740–742^. | Social activity is related to health directly and via mechanisms such as supporting coping, building general resources, altering immune function, modulating cardiovascular factors, increasing positive exposures, supporting the prediction of behaviours, increasing social control, reducing engagement in unhealthy activities, and improving healthcare performance ^743–749^. |
| Social Relationships | A number of intervention studies have shown that leisure engagement can increase social engagement and quality of social relationships ^750–752^ and these effects are supported by some systematic reviews of interventions ^694,694^. In addition, there are a range of observational ^753–755^ and many theoretical publications in this area ^621,622,625,638^, which suggest similar findings. | Social relationships are linked to health in a number of ways including through changing hormone levels, modulating cardiovascular factors, supporting emotion regulation and coping, supporting achievement, and increasing behaviours relating to the prevention of ill health ^240,655,756–758^. |
| Learning And Traits | There is a significant body of literature on how leisure activities can build learning and traits (in both children and adults), including systematic reviews, multiple qualitative studies and case studies, and experimental studies ^638,759–769^. For example, intervention studies have shown arts and leisure programmes can improve social skills and participation in adolescents with brain injuries, and decreased hyperactivity and problem behaviours in children, as well as improved motor skills ^759,762,770^. | Social and cultural learning are related to health outcomes via mechanisms such as building identity, supporting cognitive and emotional learning, activating perceptual processes, improving voice, enhancing social engagement, encouraging adaptive group behaviours, and removing barriers to healthcare ^771–775^. |
| Social Resources | Many observational studies have suggested associations between leisure activities can increased social resources ^776–780^ and this work is reported in a number of review articles ^781^. This area has been discussed extensively in theoretical publications too ^782–785^. However, there are limited intervention studies. | Social resources have been linked directly with health as well as indirectly via affecting mechanisms such as building identity, changing exposure, altering brain physiology, catalysing social actions, supporting behavioural adjustment, increasing health promotion, building group learning, increasing healthcare assets, and influencing healthcare delivery ^784,784,786–791^. |
| Group Strength | There is a growing literature on how leisure engagement can build group strength and group cohesion, including ethnographic, qualitative-interview, and intervention studies ^638,792–801^. For example, mixed methods intervention studies have found increased sense of group cohesion, social support and sense of community experienced through group leisure ^794–796^. Theoretical work has explored how ‘focal practices’ such as singing in a choir might contribute to enhanced group strength ^792^. | Group strength can affect health through mechanisms such as building general and health-related resources, decreasing negative exposures, affecting disease patterns, building capital, building group learning, increasing social control, encouraging adaptive group behaviours and reducing engagement in unhealthy activities ^790,802–804^. |
| Group Power | There is a small but growing literature on how leisure activities can enhance group power, especially within ethnic studies and feminist literature. This includes mostly in-depth qualitative studies alongside also some observational studies ^805–813^. However, to date there is a paucity of intervention studies on these mechanisms. | Factors relating to power are linked to health in a number of ways, including through reducing stigma, changing attitudes to health, increasing social responsibility, increasing health promotion, influencing healthcare delivery and improving healthcare performance ^814–817^. |
| Development Of Habit | A number of studies have shown how leisure engagement can affect the development of leisure-specific habits and thus longer-term leisure engagement. Most of these are observational studies including longitudinal analyses and cross-sectional studies ^818–824^, but early results from intervention studies including several randomised control trials (especially those addressing specific conditions such as contextual repetitions, cues and scripts) also find increases in habit formation after leisure activity interventions ^822,825–827^. | The development of habits is associated directly and indirectly with health outcomes, including through supporting emotion regulation, developing autonomy, modifying arousal, modulating brain biomarkers, catalysing social actions, enhancing behavioural activation, influencing individual choice, encouraging adaptive group behaviours, and increasing behaviours relating to the prevention and management of ill health ^323,828–832^. |
| Behavioural Decisions | There has been some research on leisure and behavioural decisions, especially focusing on the prediction of behaviours, mediated via enhanced inhibitory control, offsetting of delayed neural timing, and physiological effects. This is supported through numerous intervention studies, which demonstrate reductions in aggressive and agitated behaviour and increases in cooperation ^833–836^. Experimental studies examining the impact of music listening on the pre-frontal cortex have also reported behaviour is affected by enhanced learning and implementation of inhibitory control ^837,838^. Further, systematic reviews have suggested active leisure engagement can reduce adolescent risk behaviours ^839^. | Behavioural decisions have been related to health outcomes via mechanisms such as supporting emotion regulation, building identity, supporting overarching cognitive processes, changing attitudes to health, increasing brain activation, changing exposure, improving equality, enhancing behavioural activation, encouraging adaptive group behaviours, developing behaviours to support the delivery of healthcare, and influencing healthcare delivery ^840–845^. |
| Behavioural Drive | Aspects of behavioural engagement have been researched in relation to leisure in a number of studies, including cross-sectional studies demonstrating leisure activities can improve school achievement ^846^, and qualitative interview studies showing leisure activities can enhance generativity and inter-generational bonding ^847^. A growing number of intervention studies are now demonstrating leisure activities such as music, physical activity and virtual reality can enhance goal orientation and intrinsic motivation ^848–851^. Longitudinal analyses also seem to support these findings ^852,853^. However, there has been more of a focus on how to motivate leisure engagement rather than how leisure engagement could motivate individuals in other aspects of their lives or support wider achievement. | Behavioural drive has been associated with health both directly and via mechanisms such as developing autonomy, building general resources, modifying arousal, modulating brain biomarkers, catalysing social actions, enhancing social engagement, and increasing behaviours relating to the prevention and management of ill health ^854–857^. |
| Behavioural Development | There is a large literature on how leisure activities can support behavioural development in children, comprising experimental ^545,625,858–861^ and observational studies ^545,625,862–864^. But systematic reviews and meta-analyses of physical leisure activity in children found less research to date on behavioural mechanisms ^545,625^. | Behavioural development is itself associated with health both directly and via mechanisms such as building identity, building psychological strength, altering brain physiology, building social learning and traits, enhancing behavioural activation and supporting achievement ^865–870^. |
| Personal Location | A wide range of leisure activities have been found to affect a sense of time and place, most commonly researched through qualitative studies, longitudinal and secondary data analyses ^871–879^. For example, cross-sectional studies have reported that leisure activities can improve mental health by providing a sense of daily structure and enhancing individuals’ sense of spending their time effectively ^880^. However, to date there is limited data from intervention studies on these mechanisms, although leisure activities have been shown to positively impact participants’ relationship with the past and future amongst individuals struggling with addiction ^881^. | Personal location has been shown to affect health via mechanisms such as building identity, enhancing meaning in life, modifying arousal, changing exposure, building social identity, assisting in the formation of new habits, and increasing behaviours relating to the prevention of ill health ^882–887^. |
| Cooperation | Leisure activities have been shown to be associated with multiple aspects of cooperation. Longitudinal and qualitative studies have suggested this is mediated through enhanced social capital, trust and sense of community and reductions in anti-social behaviour and social isolation ^404,737,888–892^. There are a small number of intervention studies on these mechanisms, demonstrating exercise classes can enhance mutual support and group cohesion ^795,893^ | Cooperation is associated with health via mechanisms such as developing group values and understanding, changing attitudes to health, changing communication, building capital, increasing motivation, increasing health promotion, increasing behaviours relating to the prevention and management of ill health, and removing barriers to healthcare ^894–898^. |
| Approaches To Health | There is a growing body of largely qualitative studies and quantitative survey data showing that the leisure industry and particular leisure programmes can alter behavioural processes relating to health ^899–905^. However, longitudinal and intervention studies are scarce. | Behavioural processes relating to approaches to health are linked directly to health outcomes as well as indirectly via mechanisms such as building psychological strength, building general and health-related resources, reducing load, improving physical function, building power, improving equality, and influencing healthcare delivery ^906–909^. |
| Availability Of Assets | Whilst evidence - largely from secondary data analyses - has shown a link between leisure engagement and both leisure and healthcare assets ^910–913^, these mechanisms remain less well researched. | Increased availability of leisure assets has been linked with health via mechanisms such as eliciting affective responses, supporting group cohesion, developing the leisure industry, increasing social responsibility, and increasing behaviours relating to the prevention of ill health ^914–916^. |
| Engagement In Healthy Behaviours | There is a substantial literature on how leisure activities can increase healthy behaviours. This includes large cohort studies that demonstrate associations between leisure activities and remaining active in later life, medication adherence, better sleep quality and adolescent healthy behaviours ^917–920^. Cross-sectional data also supports this, for example finding associations between leisure in green spaces and general health ^921,922^. Ethnographies and intervention studies also provide evidence for the use of leisure activities such as arts and music to enhance healthy behaviours ^923–926^ | Health behaviours are all strongly related to mental and physical health outcomes ^927,928^. |
| Disengagement In Unhealthy Behaviours | A large number of mostly cross-sectional studies have suggested that certain types of positive leisure engagement are associated with protection against engaging in unhealthy behaviours, such as anti-social behaviour ^862,917,929–934^. There is a small but growing body of evidence from intervention studies on these mechanisms, for example through arts, music and physical activity programmes decreasing problem behaviour and sedentary activity ^833–835,935^. | Maladaptive health behaviours are also strongly related to mental and physical health outcomes ^928,936^. |
| Healthcare | Multiple reviews involving mainly intervention and qualitative studies have shown how leisure can affect healthcare delivery and performance, for example by utilising leisure activities (such as music and arts) in healthcare spaces, or utilising leisure spaces (such as museums and parks) for healthcare delivery ^937–945^ . | The delivery and performance of healthcare is strongly linked with health outcomes ^946–949^. |

# **Factors that can predict leisure engagement or moderate the mechanisms linking leisure activities with health**

Whilst remembering that boundary between leisure activities and their context is blurred ^950^, it is helpful to consider which factors could affect leisure participation and/or moderate the activation and impact of any mechanisms. A summary of these is presented below.

| Micro-level | *References:* ^951–954^ ^955^ |
| --- | --- |
| CAPABILITIES |  |
| Psychological capabilities | E.g. personality traits, attitudes, cognitive abilities and mental health |
| Physical capabilities | E.g. physical health, physical ability, and biological predispositions |
| OPPORTUNITIES |  |
| Physical opportunities | E.g. early life exposure to leisure, socio-economic resources, education |
| Social opportunities | E.g. education, family engagement with leisure |
| MOTIVATIONS |  |
| Automatic motivations | E.g. likes and dislikes, individual talent, perceptions of benefits, and previous childhood and adulthood leisure patterns |
| Reflective motivations | E.g. preconceptions or beliefs about leisure, self-identity and goals |
| Meso-level | *References:* ^240,951–953^ ^884^ |
| SPACE & PLACE |  |
| Access to leisure | E.g. the quantity, quality, diversity and accessibility of activities available |
| Physical environment | E.g. green spaces, community spaces, play areas, climate, pollution and natural resources |
| Infrastructure | E.g. education, transport, street cleaning, lighting, policing, health services and welfare services, housing, employment and area deprivation |
| PEOPLE |  |
| Social networks | E.g. frequency of social contact, extent of reciprocity within social relationships, and the types of social transactions and support |
| Social influence | E.g. norms and values, social cohesion, peer pressure and social comparisons |
| Community culture | E.g. community integration, levels of crime, threats to personal safety, networks of community support, and the political, ethnic, economic and religious history of a community |
| Macro-level | *References:* ^240,951–953^ |
| Socio-economic factors | E.g. employment, inequality, conflict, poverty, economic stability, and the structure of the labour market |
| Cultural factors | E.g. discrimination, racism, sexism and equal opportunities |
| Political factors | E.g. power, agendas, laws, tariffs, policies and restrictions |

# **References**

1 Cohn MA, Fredrickson BL. Positive Emotions. *The Oxford Handbook of Positive Psychology* 2009; published online July 30. DOI:10.1093/oxfordhb/9780195187243.013.0003.

2 Selye H. Stress without Distress. In: Serban G, ed. Psychopathology of Human Adaptation. Boston, MA: Springer US, 1976: 137–46.

3 Kuppens P, Tuerlinckx F, Russell JA, Barrett LF. The relation between valence and arousal in subjective experience. *Psychological Bulletin* 2013; **139**: 917–40.

4 Bramble B. The distinctive feeling theory of pleasure. *Philos Stud* 2013; **162**: 201–17.

5 Gross JJ, Thompson R. Emotion Regulation: Conceptual Foundations. *Handbook of Emotion Regulation* 2007; : 3–27.

6 Koole SL. The psychology of emotion regulation: An integrative review. *Cognition and Emotion* 2009; **23**: 4–41.

7 Rothbart MK, Ziaie H, O’Boyle CG. Self-regulation and emotion in infancy. *New Directions for Child and Adolescent Development* 1992; **1992**: 7–23.

8 Lochner K. Affect, Mood, and Emotions. In: Lochner K, ed. Successful Emotions: How Emotions Drive Cognitive Performance. Wiesbaden: Springer Fachmedien, 2016: 43–67.

9 Fredrickson BL. The broaden-and-build theory of positive emotions. *Philos Trans R Soc Lond B Biol Sci* 2004; **359**: 1367–78.

10 Lazarus RS, Folkman S. Stress, appraisal, and coping. Springer Pub. Co., 1984.

11 Alicke MD, Sedikides C. Self-enhancement and self-protection: What they are and what they do. *European Review of Social Psychology* 2009; **20**: 1–48.

12 Cohen S, Wills TA. Stress, social support, and the buffering hypothesis. *Psychol Bull* 1985; **98**: 310–57.

13 Vasey MW, Borkovec TD. A catastrophizing assessment of worrisome thoughts. *Cognitive Therapy and Research* 1992; **16**: 505–520.

14 Klonsky ED, May AM, Saffer BY. Suicide, Suicide Attempts, and Suicidal Ideation. *Annu Rev Clin Psychol* 2016; **12**: 307–30.

15 Greenberg J, Solomon S, Pyszczynski T. Terror Management Theory of Self-Esteem and Cultural Worldviews: Empirical Assessments and Conceptual Refinements. In: Zanna MP, ed. Advances in Experimental Social Psychology. Academic Press, 1997: 61–139.

16 Shuchter SR, Zisook S. The course of normal grief. In: Handbook of bereavement: Theory, research, and intervention. New York, NY, US: Cambridge University Press, 1993: 23–43.

17 Schwartz RM. The internal dialogue: On the asymmetry between positive and negative coping thoughts. *Cognitive therapy and research* 1986; **10**: 591–605.

18 Radley A, Green R. Illness as adjustment: a methodology and conceptual framework. *Sociology of Health & Illness* 1987; **9**: 179–207.

19 Alkhalifa EM. Towards demonstrating limited cognitive reception bandwidth. In: 2008 Eighth IEEE International Conference on Advanced Learning Technologies. IEEE, 2008: 913–916.

20 Bénabou R, Tirole J. Self-confidence and personal motivation. *The Quarterly Journal of Economics* 2002; **117**: 871–915.

21 Rutter M. Resilience: Some conceptual considerations. *Journal of Adolescent Health* 1993; **14**: 626–31.

22 Ryan RM, Deci EL. From Ego Depletion to Vitality: Theory and Findings Concerning the Facilitation of Energy Available to the Self. *Social and Personality Psychology Compass* 2008; **2**: 702–17.

23 Agnati LF, Barlow PW, Baluška F, *et al.* A new theoretical approach to the functional meaning of sleep and dreaming in humans based on the maintenance of ‘predictive psychic homeostasis’. *Communicative & Integrative Biology* 2011; **4**: 640–54.

24 Kleiber DA, Hutchinson SL, Williams R. Leisure as a resource in transcending negative life events: Self-protection, self-restoration, and personal transformation. *Leisure Sciences* 2002; **24**: 219–235.

25 Dugas MJ, Freeston MH, Ladouceur R. Intolerance of uncertainty and problem orientation in worry. *Cognitive therapy and research* 1997; **21**: 593–606.

26 Moors A, De Houwer J. Automatic processing of dominance and submissiveness. *Experimental Psychology* 2005; **52**: 296–302.

27 Wolkon GH. Crisis theory, the application for treatment, and dependency. *Comprehensive Psychiatry* 1972; **13**: 459–64.

28 Pearlin LI. The sociological study of stress. *Journal of health and social behavior* 1989; : 241–256.

29 Hobfoll SE. Social and Psychological Resources and Adaptation. *Review of General Psychology* 2002; **6**: 307–24.

30 Giddens A. Modernity and Self-identity: Self and Society in the Late Modern Age. Stanford University Press, 1991.

31 Markus HR, Kitayama S. Culture and the self: Implications for cognition, emotion, and motivation. *Psychological review* 1991; **98**: 224.

32 Bem DJ. Self-Perception Theory. In: Berkowitz L, ed. Advances in Experimental Social Psychology. Academic Press, 1972: 1–62.

33 Higgins ET. Self-Discrepancy Theory: What Patterns of Self-Beliefs Cause People to Suffer? In: Berkowitz L, ed. Advances in Experimental Social Psychology. Academic Press, 1989: 93–136.

34 Vilanova F, Beria FM, Costa ÂB, Koller SH. Deindividuation: From Le Bon to the social identity model of deindividuation effects. *Cogent Psychology* 2017; **4**: 1308104.

35 Green M, Green MS. Self-Expression. OUP Oxford, 2007.

36 Atance CM, Meltzoff AN. My future self: Young children’s ability to anticipate and explain future states. *Cognitive Development* 2005; **20**: 341–361.

37 Wicklund RA. Objective Self-Awareness11Much of the research reported in this paper as well as the writing of this paper were supported by NSF Grant GS-31890. Sharon S. Brehm, William J. Ickes, Michael F. Scheier, and Melvin L. Snyder are acknowledged for their suggestions and insightful criticisms. In: Berkowitz L, ed. Advances in Experimental Social Psychology. Academic Press, 1975: 233–75.

38 Crocker J, Knight KM. Contingencies of self-worth. *Current directions in psychological science* 2005; **14**: 200–203.

39 Scambler G. Health-related stigma. *Sociology of Health & Illness* 2009; **31**: 441–55.

40 Steele CM. The Psychology of Self-Affirmation: Sustaining the Integrity of the Self. In: Berkowitz L, ed. Advances in Experimental Social Psychology. Academic Press, 1988: 261–302.

41 Kaplan HB, Pokorny AD. Self-derogation and psychosocial adjustment. *Journal of Nervous and Mental Disease* 1969; **149**: 421–34.

42 Harter S. Authenticity. In: Handbook of positive psychology. New York, NY, US: Oxford University Press, 2002: 382–94.

43 Levinson W, Dunn PM. Coping With Fallibility. *JAMA* 1989; **261**: 2252–2252.

44 Charmaz K. Loss of self: a fundamental form of suffering in the chronically ill. *Sociology of Health & Illness* 1983; **5**: 168–95.

45 Bury M. Chronic illness as biographical disruption. *Sociology of health & illness* 1982; **4**: 167–182.

46 Bluck S, Levine LJ. Reminiscence as autobiographical memory: a catalyst for reminiscence theory development. *Ageing & Society* 1998; **18**: 185–208.

47 Levine S, Kozloff MA. The sick role: Assessment and overview. *Annual Review of Sociology* 1978; **4**: 317–343.

48 Bury M. Illness narratives: fact or fiction? *Sociology of Health & Illness* 2001; **23**: 263–85.

49 Whitehead A. The medical humanities: A literary perspective. In: Medicine, Health and the Arts. Routledge, 2013: 119–139.

50 Zillig LMP, Hemenover SH, Dienstbier RA. What Do We Assess when We Assess a Big 5 Trait? A Content Analysis of the Affective, Behavioral, and Cognitive Processes Represented in Big 5 Personality Inventories. *Pers Soc Psychol Bull* 2002; **28**: 847–58.

51 Schaefer JA, Moos RH. Bereavement experiences and personal growth. 2001.

52 Higgins ET. Regulatory focus theory. In: Handbook of theories of social psychology, Vol. 1. Thousand Oaks, CA: Sage Publications Ltd, 2012: 483–504.

53 Levenson MR, Jennings PA, Aldwin CM, Shiraishi RW. Self-Transcendence: Conceptualization and Measurement. *Int J Aging Hum Dev* 2005; **60**: 127–43.

54 Seligman ME, Csikszentmihalyi M. Positive psychology. An introduction. *Am Psychol* 2000; **55**: 5–14.

55 Antonovsky A. The Sense of Coherence as a determinant health. *Behavioral health* 1984.

56 Strauss AL. Social Psychology and Human Values. Routledge, 2017 DOI:10.4324/9781315129730.

57 Emmons RA, McCullough ME. The Psychology of Gratitude. Oxford University Press, 2004.

58 Erikson EH. Insight and responsibility. WW Norton & Company, 1994.

59 Buss DM, Scheier MF. Self-consciousness, self-awareness, and self-attribution. *Journal of Research in Personality* 1976; **10**: 463–468.

60 Deci EL, Ryan RM. Human autonomy. In: Efficacy, agency, and self-esteem. Springer, 1995: 31–49.

61 Bandura A. Toward a psychology of human agency. *Perspectives on psychological science* 2006; **1**: 164–180.

62 Zimmerman MA. Psychological empowerment: Issues and illustrations. *American journal of community psychology* 1995; **23**: 581–599.

63 Karasek Jr RA. Job demands, job decision latitude, and mental strain: Implications for job redesign. *Administrative science quarterly* 1979; : 285–308.

64 Solso RL, MacLin MK, MacLin OH. Cognitive psychology. Pearson Education New Zealand, 2005.

65 Eysenck HJ. Creativity, personality and the convergent-divergent continuum. 2003.

66 Vernon MD. Cognitive inference in perceptual activity. *British Journal of Psychology* 1957; **48**: 35–47.

67 Sherman SJ, Corty E. Cognitive heuristics. In: Handbook of social cognition, Vol 1. Mahwah, NJ, US: Lawrence Erlbaum Associates Publishers, 1984: 189–286.

68 Noack H, Lövdén M, Schmiedek F. On the validity and generality of transfer effects in cognitive training research. *Psychological research* 2014; **78**: 773–789.

69 Zangwill N. Aesthetic Judgment. In: Zalta EN, ed. The Stanford Encyclopedia of Philosophy, Spring 2019. Metaphysics Research Lab, Stanford University, 2019. https://plato.stanford.edu/archives/spr2019/entries/aesthetic-judgment/ (accessed Feb 17, 2020).

70 Heberlein AS, Atkinson AP. Neuroscientific Evidence for Simulation and Shared Substrates in Emotion Recognition: Beyond Faces. *Emotion Review* 2009; **1**: 162–77.

71 Carruthers P, Smith PK. Theories of theories of mind. Cambridge University Press, 1996.

72 Katz RL. Empathy: Its nature and uses. Oxford, England: Free Press Glencoe, 1964.

73 Batson CD, Fultz J, Schoenrade PA. Distress and Empathy: Two Qualitatively Distinct Vicarious Emotions with Different Motivational Consequences. *Journal of Personality* 1987; **55**: 19–39.

74 Cassell EJ. Compassion. 2002.

75 Kochanska G. Beyond cognition: Expanding the search for the early roots of internalization and conscience. 1994.

76 Piaget J. The construction of reality in the child. New York, NY, US: Basic Books, 1954 DOI:10.1037/11168-000.

77 Holmes J. Mentalisation and metaphor in poetry and psychotherapy. *Advances in Psychiatric Treatment* 2008; **14**: 167–171.

78 Meins E, Fernyhough C, Arnott B, Leekam SR, de Rosnay M. Mind-mindedness and theory of mind: Mediating roles of language and perspectival symbolic play. *Child development* 2013; **84**: 1777–1790.

79 Wänke M, Hansen J. Relative Processing Fluency. *Curr Dir Psychol Sci* 2015; **24**: 195–9.

80 Piaget J. Part I: Cognitive development in children: Piaget development and learning. *Journal of research in science teaching* 1964; **2**: 176–186.

81 Heimberg RG. Cognitive-behavioral therapy for social anxiety disorder: current status and future directions. *Biological psychiatry* 2002; **51**: 101–108.

82 Mezirow J. Transformative Learning: Theory to Practice. *New Directions for Adult and Continuing Education* 1997; **1997**: 5–12.

83 Gardner H. Multiple intelligences. Minnesota Center for Arts Education, 1992.

84 Berlyne DE. A Theory of Human Curiosity. *British Journal of Psychology General Section* 1954; **45**: 180–91.

85 Rhodes M. An analysis of creativity. *The Phi Delta Kappan* 1961; **42**: 305–310.

86 Latour B. Visualisation and cognition: Drawing things together. 2012.

87 Morgan JJ. Imagination. 1931.

88 Li J-Q, Tan L, Wang H-F, *et al.* Risk factors for predicting progression from mild cognitive impairment to Alzheimer’s disease: a systematic review and meta-analysis of cohort studies. *J Neurol Neurosurg Psychiatry* 2016; **87**: 476–84.

89 Wechsler D. Intellectual Development and Psychological Maturity. *Child Development* 1950; **21**: 45–50.

90 Bourdieu P. The forms of capital. *Cultural theory: An anthology* 1986; **1**: 81–93.

91 Alloway TP, Alloway RG. Investigating the predictive roles of working memory and IQ in academic attainment. *Journal of Experimental Child Psychology* 2010; **106**: 20–9.

92 Sen AK. Inequality re-examined. Oxford: Clarendon Press, 1992.

93 Dweck CS. Implicit theories as organizers of goals and behavior. In: The psychology of action: Linking cognition and motivation to behavior. New York, NY, US: Guilford Press, 1996: 69–90.

94 Leventhal H, Diefenbach M, Leventhal EA. Illness cognition: Using common sense to understand treatment adherence and affect cognition interactions. *Cogn Ther Res* 1992; **16**: 143–63.

95 Gould SJ. Health Consciousness and Health Behavior: The Application of a New Health Consciousness Scale. *American Journal of Preventive Medicine* 1990; **6**: 228–37.

96 Nutbeam D. The evolving concept of health literacy. *Social science & medicine* 2008; **67**: 2072–2078.

97 Voronka J. The Politics of ‘people with lived experience’ Experiential Authority and the Risks of Strategic Essentialism. *Philosophy, Psychiatry, & Psychology* 2016; **23**: 189–201.

98 Vlaev I, King D, Dolan P, Darzi A. The Theory and Practice of “Nudging”: Changing Health Behaviors. *Public Administration Review* 2016; **76**: 550–61.

99 Kirmayer LJ. The cultural diversity of healing: meaning, metaphor and mechanism. *Br Med Bull* 2004; **69**: 33–48.

100 Nussbaum P of L and EU of CLSM, Nussbaum M, Sen A, Sen MA. The Quality of Life. Oxford University Press, 1993.

101 Karterud S, Stone WN. The Group Self: A Neglected Aspect of Group Psychotherapy: *Group Analysis* 2016; published online June 30. DOI:10.1177/0533316403036001198.

102 McMahon C. Collective Rationality and Collective Reasoning. Cambridge University Press, 2001.

103 Hedden T, Ketay S, Aron A, Markus HR, Gabrieli JDE. Cultural influences on neural substrates of attentional control. *Psychol Sci* 2008; **19**: 12–7.

104 Stahl G. Building Collaborative Knowing. In: Strijbos J-W, Kirschner PA, Martens RL, eds. What We Know About CSCL: And Implementing It In Higher Education. Dordrecht: Springer Netherlands, 2004: 53–85.

105 Baerveldt C, Verheggen T. Enactivism. *The Oxford Handbook of Culture and Psychology* 2012; published online May 2. DOI:10.1093/oxfordhb/9780195396430.013.0009.

106 Kurzman C. Meaning-making in social movements. *Anthropological Quarterly* 2008; **81**: 5–15.

107 Cialdini RB, Trost MR. Social influence: Social norms, conformity and compliance. 1998.

108 Merton RK. Social Structure and Anomie. *American Sociological Review* 1938; **3**: 672–82.

109 Teymoori A, Bastian B, Jetten J. Towards a Psychological Analysis of Anomie. *Political Psychology* 2017; **38**: 1009–23.

110 Feinberg J. Collective responsibility. *The Journal of Philosophy* 1968; **65**: 674–688.

111 Freire P. Cultural Action for Freedom. Penguin, 1972.

112 Becker HS. Labelling theory reconsidered 1. In: Deviance and social control. Routledge, 2018: 41–66.

113 Fiske ST. Stereotyping, prejudice, and discrimination. *The handbook of social psychology* 1998; **2**: 357–411.

114 Králová J. What is social death? *Contemporary Social Science* 2015; **10**: 235–48.

115 Conrad P, Barker KK. The Social Construction of Illness: Key Insights and Policy Implications. *J Health Soc Behav* 2010; **51**: S67–79.

116 Ballard K, Elston MA. Medicalisation: A Multi-dimensional Concept. *Soc Theory Health* 2005; **3**: 228–41.

117 Davis DL, Guarnaccia PJ. Health, culture and the nature of nerves: introduction. 1989.

118 Moon G. Risk and protection: the discourse of confinement in contemporary mental health policy. *Health & place* 2000; **6**: 239–250.

119 Faezipour M, Ferreira S. A System Dynamics Perspective of Patient Satisfaction in Healthcare. In: CSER. 2013: 148–156.

120 Wirth L. The Problem of Minority Groups. Irvington Pub, 1993.

121 Foulkes SH. The group as matrix of the individual’s mental life. *Group therapy* 1973; : 211–220.

122 Buck R, VanLear CA. Verbal and Nonverbal Communication: Distinguishing Symbolic, Spontaneous, and Pseudo-Spontaneous Nonverbal Behavior. *J Commun* 2002; **52**: 522–41.

123 Aunger R. Darwinizing culture: The status of memetics as a science. 2001.

124 Hewitt JP, Shulman D. Self and society: A symbolic interactionist social psychology. Allyn and Bacon Boston, 1979.

125 von Scheve C, Ismer S. Towards a Theory of Collective Emotions. *Emotion Review* 2013; **5**: 406–13.

126 Gillespie DF. Barton’s theory of collective stress is a classic and worth testing. *International journal of mass emergencies and disasters* 1988; **6**: 345–361.

127 Shilling C, Mellor PA. Durkheim, Morality and Modernity: Collective Effervescence, Homo Duplex and the Sources of Moral Action. *The British Journal of Sociology* 1998; **49**: 193–209.

128 Gotow N, Kobayakawa T. Simultaneity judgment using olfactory–visual, visual–gustatory, and olfactory–gustatory combinations. *PLoS One* 2017; **12**. DOI:10.1371/journal.pone.0174958.

129 Tuthill JC, Azim E. Proprioception. *Current Biology* 2018; **28**: R194–203.

130 Vaitl D. Interoception. *Biological Psychology* 1996; **42**: 1–27.

131 Proske U. Kinesthesia: The role of muscle receptors. *Muscle & Nerve* 2006; **34**: 545–58.

132 Nebylitsyn VD, Rozhdestvenskaya VI, Teplov BM. Concerning the Interrelation between Absolute Sensitivity and Strength of the Nervous System. *Quarterly Journal of Experimental Psychology* 1960; **12**: 17–25.

133 Norwich K. Le Chatelier’s principle in sensation and perception: fractal-like enfolding at different scales. *Front Physiol* 2010; **1**. DOI:10.3389/fphys.2010.00017.

134 Solomon P, Leiderman PH, Mendelson J, Wexler D. Sensory deprivation. *AJP* 1957; **114**: 357–63.

135 Stein BE, Stanford TR. Multisensory integration: current issues from the perspective of the single neuron. *Nature Reviews Neuroscience* 2008; **9**: 255–266.

136 Latremoliere A, Woolf CJ. Central Sensitization: A Generator of Pain Hypersensitivity by Central Neural Plasticity. *J Pain* 2009; **10**: 895–926.

137 Moayedi M, Davis KD. Theories of pain: from specificity to gate control. *Journal of Neurophysiology* 2012; **109**: 5–12.

138 Santangelo V, Cavallina C, Colucci P, *et al.* Enhanced brain activity associated with memory access in highly superior autobiographical memory. *PNAS* 2018; **115**: 7795–800.

139 Strandgaard S, Paulson OB. Regulation of cerebral blood flow in health and disease. *J Cardiovasc Pharmacol* 1992; **19 Suppl 6**: S89-93.

140 Joris PJ, Mensink RP, Adam TC, Liu TT. Cerebral Blood Flow Measurements in Adults: A Review on the Effects of Dietary Factors and Exercise. *Nutrients* 2018; **10**. DOI:10.3390/nu10050530.

141 Ishizu T. Functional Neuroimaging in Empirical Aesthetics and Neuroaesthetics. *The Oxford Handbook of Empirical Aesthetics* 2019; published online Aug 12. DOI:10.1093/oxfordhb/9780198824350.013.14.

142 Chatterjee A, Vartanian O. Neuroscience of aesthetics. *Annals of the New York Academy of Sciences* 2016; **1369**: 172–194.

143 Rizzolatti G, Craighero L. The mirror-neuron system. *Annu Rev Neurosci* 2004; **27**: 169–92.

144 Lu LH, Dapretto M, O’Hare ED, *et al.* Relationships between Brain Activation and Brain Structure in Normally Developing Children. *Cereb Cortex* 2009; **19**: 2595–604.

145 Purves D, Augustine GJ, Fitzpatrick D, *et al.* Neuroscience, 2008. *De Boeck, Sinauer, Sunderland, Mass* 2014; : 15–16.

146 Gage FH. Neurogenesis in the adult brain. *Journal of Neuroscience* 2002; **22**: 612–613.

147 Pittenger C, Duman RS. Stress, Depression, and Neuroplasticity: A Convergence of Mechanisms. *Neuropsychopharmacology* 2008; **33**: 88–109.

148 Lupien SJ, Juster R-P, Raymond C, Marin M-F. The effects of chronic stress on the human brain: From neurotoxicity, to vulnerability, to opportunity. *Frontiers in Neuroendocrinology* 2018; **49**: 91–105.

149 Stern Y. Cognitive reserve in ageing and Alzheimer’s disease. *Lancet Neurol* 2012; **11**: 1006–12.

150 Wu Y-T, Teale J, Matthews FE, Brayne C, Woods B, Clare L. Lifestyle factors, cognitive reserve, and cognitive function: results from the Cognitive Function and Ageing Study Wales, a population-based cohort. *The Lancet* 2016; **388**: S114.

151 Nestler EJ, Carlezon Jr WA. The mesolimbic dopamine reward circuit in depression. *Biological psychiatry* 2006; **59**: 1151–1159.

152 Volkow ND, Wang G-J, Fowler JS, Tomasi D, Telang F. Addiction: beyond dopamine reward circuitry. *Proceedings of the National Academy of Sciences* 2011; **108**: 15037–15042.

153 Lucki I. The spectrum of behaviors influenced by serotonin. *Biological psychiatry* 1998.

154 Carter CS. Oxytocin and sexual behavior. *Neuroscience & Biobehavioral Reviews* 1992; **16**: 131–144.

155 Bartz JA, Zaki J, Bolger N, Ochsner KN. Social effects of oxytocin in humans: context and person matter. *Trends Cogn Sci (Regul Ed)* 2011; **15**: 301–9.

156 Akil H. On the role of endorphins in pain modulation. *The Neural Basis of Behavior* 2012; : 311.

157 Hegadoren KM, O’Donnell T, Lanius R, Coupland NJ, Lacaze-Masmonteil N. The role of β-endorphin in the pathophysiology of major depression. *Neuropeptides* 2009; **43**: 341–53.

158 Bisogno T. Endogenous cannabinoids: structure and metabolism. *Journal of Neuroendocrinology* 2008; **20**: 1–9.

159 A. Quera Salva M, Hartley S, Barbot F, C. Alvarez J, Lofaso F, Guilleminault C. Circadian Rhythms, Melatonin and Depression. 2011. DOI:info:doi/10.2174/138161211796197188.

160 Hampe CS, Mitoma H, Manto M. GABA and Glutamate: Their Transmitter Role in the CNS and Pancreatic Islets. *GABA And Glutamate - New Developments In Neurotransmission Research* 2017; published online Dec 20. DOI:10.5772/intechopen.70958.

161 Lurie DI. An Integrative Approach to Neuroinflammation in Psychiatric disorders and Neuropathic Pain. *J Exp Neurosci* 2018; **12**. DOI:10.1177/1179069518793639.

162 Koch CE, Leinweber B, Drengberg BC, Blaum C, Oster H. Interaction between circadian rhythms and stress. *Neurobiology of Stress* 2017; **6**: 57–67.

163 Harrison L, Loui P. Thrills, chills, frissons, and skin orgasms: toward an integrative model of transcendent psychophysiological experiences in music. *Frontiers in psychology* 2014; **5**: 790.

164 Prokasy W. Electrodermal activity in psychological research. Elsevier, 2012.

165 McCorry LK. Physiology of the Autonomic Nervous System. *Am J Pharm Educ* 2007; **71**. https://www.ncbi.nlm.nih.gov/pmc/articles/PMC1959222/ (accessed Jan 17, 2020).

166 Curtis BM, O’Keefe JH. Autonomic tone as a cardiovascular risk factor: the dangers of chronic fight or flight. *Mayo Clin Proc* 2002; **77**: 45–54.

167 Schnitzler A, Gross J. Normal and pathological oscillatory communication in the brain. *Nature Reviews Neuroscience* 2005; **6**: 285–96.

168 Will U, Berg E. Brain wave synchronization and entrainment to periodic acoustic stimuli. *Neuroscience Letters* 2007; **424**: 55–60.

169 Brown BB. Stress and the art of biofeedback. Harper & Row, 1977.

170 Groves DA, Brown VJ. Vagal nerve stimulation: a review of its applications and potential mechanisms that mediate its clinical effects. *Neuroscience & Biobehavioral Reviews* 2005; **29**: 493–500.

171 Ader R. Psychoneuroimmunology. Academic Press, 2007.

172 Chrousos GP. Stress and disorders of the stress system. *Nature Reviews Endocrinology* 2009; **5**: 374–81.

173 Skuse D, Albanese A, Stanhope R, Gilmour J, Voss L. A new stress-related syndrome of growth failure and hyperphagia in children, associated with reversibility of growth-hormone insufficiency. *The Lancet* 1996; **348**: 353–358.

174 Handa RJ, Weiser MJ. Gonadal steroid hormones and the hypothalamo–pituitary–adrenal axis. *Frontiers in Neuroendocrinology* 2014; **35**: 197–220.

175 Rosano GM, Panina G. Oestrogens and the heart. *Therapie* 1999; **54**: 381–5.

176 Segerstrom SC, Miller GE. Psychological stress and the human immune system: a meta-analytic study of 30 years of inquiry. *Psychol Bull* 2004; **130**: 601–30.

177 DiLoreto R, Murphy CT. The cell biology of aging. *Mol Biol Cell* 2015; **26**: 4524–31.

178 Glaser R, Kiecolt-Glaser JK. Stress-induced immune dysfunction: implications for health. *Nat Rev Immunol* 2005; **5**: 243–51.

179 Friedman W. Chapter 29 - Growth Factors. In: Brady ST, Siegel GJ, Albers RW, Price DL, eds. Basic Neurochemistry (Eighth Edition). New York: Academic Press, 2012: 546–57.

180 Dowlati Y, Herrmann N, Swardfager W, *et al.* A Meta-Analysis of Cytokines in Major Depression. *Biological Psychiatry* 2010; **67**: 446–57.

181 Aschbacher K, O’Donovan A, Wolkowitz OM, Dhabhar FS, Su Y, Epel E. Good stress, bad stress and oxidative stress: insights from anticipatory cortisol reactivity. *Psychoneuroendocrinology* 2013; **38**: 1698–708.

182 Moan J, Porojnicu AC, Dahlback A, Setlow RB. Addressing the health benefits and risks, involving vitamin D or skin cancer, of increased sun exposure. *Proceedings of the National Academy of Sciences* 2008; **105**: 668–673.

183 Kudielka BM, Schommer NC, Hellhammer DH, Kirschbaum C. Acute HPA axis responses, heart rate, and mood changes to psychosocial stress (TSST) in humans at different times of day. *Psychoneuroendocrinology* 2004; **29**: 983–92.

184 Mather M, Thayer JF. How heart rate variability affects emotion regulation brain networks. *Current Opinion in Behavioral Sciences* 2018; **19**: 98–104.

185 Lacy CR, Contrada RJ, Robbins ML, *et al.* Coronary vasoconstriction induced by mental stress (simulated public speaking). *The American Journal of Cardiology* 1995; **75**: 503–5.

186 Chen X, Wang Y. Tracking of blood pressure from childhood to adulthood: A systematic review and meta-regression analysis. *Circulation* 2008; **117**: 3171–80.

187 Light KC. Hypertension and the Reactivity Hypothesis: The Next Generation. *Psychosomatic Medicine* 2001; **63**: 744.

188 Zanstra YJ, Johnston DW. Cardiovascular reactivity in real life settings: Measurement, mechanisms and meaning. *Biol Psychol* 2011; **86**: 98–105.

189 Adair TH, Montani J-P. Overview of Angiogenesis. Morgan & Claypool Life Sciences, 2010 https://www.ncbi.nlm.nih.gov/books/NBK53238/ (accessed Jan 17, 2020).

190 Vlachopoulos C, Kosmopoulou F, Alexopoulos N, Ioakeimidis N, Siasos G, Stefanadis C. Acute mental stress has a prolonged unfavorable effect on arterial stiffness and wave reflections. *Psychosom Med* 2006; **68**: 231–7.

191 Young DB. Cardiac Function. Morgan & Claypool Life Sciences, 2010 https://www.ncbi.nlm.nih.gov/books/NBK54477/ (accessed Feb 23, 2020).

192 Goetsch VL, Wiebe DJ, Veltum LG, van Dorsten B. Stress and blood glucose in type II diabetes mellitus. *Behaviour Research and Therapy* 1990; **28**: 531–7.

193 Grant N, Hamer M, Steptoe A. Social Isolation and Stress-related Cardiovascular, Lipid, and Cortisol Responses. *Ann Behav Med* 2009; **37**: 29–37.

194 Steptoe A, Brydon L. Associations between acute lipid stress responses and fasting lipid levels 3 years later. *Health Psychol* 2005; **24**: 601–7.

195 Hamer M, Molloy GJ. Cross-sectional and longitudinal associations between anemia and depressive symptoms in the English Longitudinal Study of Ageing. *J Am Geriatr Soc* 2009; **57**: 948–9.

196 Cornelisse-Vermaat JR, Antonides G, Van Ophem JAC, Van Den Brink HM. Body Mass Index, Perceived Health, and Happiness: Their Determinants and Structural Relationships. *Soc Indic Res* 2006; **79**: 143–58.

197 Harris TB, Visser M, Everhart J, *et al.* Waist circumference and sagittal diameter reflect total body fat better than visceral fat in older men and women: the Health, Aging and Body Composition Study. *Annals of the New York Academy of Sciences* 2000; **904**: 462–473.

198 Flandroy L, Poutahidis T, Berg G, *et al.* The impact of human activities and lifestyles on the interlinked microbiota and health of humans and of ecosystems. *Science of The Total Environment* 2018; **627**: 1018–38.

199 Poutahidis T, Varian BJ, Levkovich T, *et al.* Dietary microbes modulate transgenerational cancer risk. *Cancer research* 2015; **75**: 1197–1204.

200 Turnbaugh PJ, Ley RE, Hamady M, Fraser-Liggett CM, Knight R, Gordon JI. The Human Microbiome Project. *Nature* 2007; **449**: 804–10.

201 Hochschild J. Functional Anatomy for Physical Therapists, 1 edition. Stuttgart: Thieme, 2015.

202 Niemi P, Näätänen R. Foreperiod and simple reaction time. *Psychological Bulletin* 1981; **89**: 133–62.

203 Layne JE, Nelson ME. The effects of progressive resistance training on bone density: a review. *Medicine & Science in Sports & Exercise* 1999; **31**: 25.

204 LaPlante MP. The Classic Measure of Disability in Activities of Daily Living Is Biased by Age but an Expanded IADL/ADL Measure Is Not. *J Gerontol B Psychol Sci Soc Sci* 2010; **65B**: 720–32.

205 Robbins AS, Rubenstein LZ, Josephson KR, Schulman BL, Osterweil D, Fine G. Predictors of falls among elderly people. Results of two population-based studies. *Arch Intern Med* 1989; **149**: 1628–33.

206 Hughes JMB. Physiology and practice of pulmonary function. Association for Respiratory Technology and Physiology, 2009.

207 Shipley KG, McAfee JG. Assessment in speech-language pathology: A resource manual. Nelson Education, 2015.

208 Chrousos GP, Gold PW. The Concepts of Stress and Stress System Disorders: Overview of Physical and Behavioral Homeostasis. *JAMA* 1992; **267**: 1244–52.

209 Sherrington CS. Experiments on the value of vascular and visceral factors for the genesis of emotion. *Proceedings of the Royal Society of London* 1900; **66**: 390–403.

210 Chalder T, Berelowitz G, Pawlikowska T, *et al.* Development of a fatigue scale. *Journal of Psychosomatic Research* 1993; **37**: 147–53.

211 McEwen BS. Allostasis and Allostatic Load: Implications for Neuropsychopharmacology. *Neuropsychopharmacology* 2000; **22**: 108–24.

212 Gunnar MR, Herrera AM. The Development of Stress Reactivity. *The Oxford Handbook of Developmental Psychology, Vol 2* 2013; published online March 14. DOI:10.1093/oxfordhb/9780199958474.013.0003.

213 Geronimus AT. The weathering hypothesis and the health of African-American women and infants: evidence and speculations. *Ethnicity & disease* 1992; **2**: 207–221.

214 Foster H, Hagan J, Brooks-Gunn J. Growing up Fast: Stress Exposure and Subjective “Weathering” in Emerging Adulthood. *J Health Soc Behav* 2008; **49**: 162–77.

215 Hertzman C. The biological embedding of early experience and its effects on health in adulthood. *Annals of the New York Academy of Sciences* 1999; **896**: 85–95.

216 Clegg A, Young J, Iliffe S, Rikkert MO, Rockwood K. Frailty in elderly people. *The Lancet* 2013; **381**: 752–762.

217 Flies EJ, Skelly C, Negi SS, *et al.* Biodiverse green spaces: a prescription for global urban health. *Frontiers in Ecology and the Environment* 2017; **15**: 510–516.

218 Aerts R, Honnay O, Van Nieuwenhuyse A. Biodiversity and human health: mechanisms and evidence of the positive health effects of diversity in nature and green spaces. *Br Med Bull* 2018; **127**: 5–22.

219 Li Q, Kobayashi M, Wakayama Y, *et al.* Effect of Phytoncide from Trees on Human Natural Killer Cell Function. *Int J Immunopathol Pharmacol* 2009; **22**: 951–9.

220 Jiang S-Y, Ma A, Ramachandran S. Negative Air Ions and Their Effects on Human Health and Air Quality Improvement. *Int J Mol Sci* 2018; **19**. DOI:10.3390/ijms19102966.

221 Ege MJ, Mayer M, Normand A-C, *et al.* Exposure to environmental microorganisms and childhood asthma. *New England Journal of Medicine* 2011; **364**: 701–709.

222 Mathur MB, Epel E, Kind S, *et al.* Perceived Stress and Telomere Length: A Systematic Review, Meta-Analysis, and Methodologic Considerations for Advancing the Field. *Brain Behav Immun* 2016; **54**: 158–69.

223 Kim D, Kubzansky LD, Baccarelli A, *et al.* Psychological factors and DNA methylation of genes related to immune/inflammatory system markers: the VA Normative Aging Study. *BMJ Open* 2016; **6**: e009790.

224 Turecki G, Meaney MJ. Effects of the Social Environment and Stress on Glucocorticoid Receptor Gene Methylation: A Systematic Review. *Biological Psychiatry* 2016; **79**: 87–96.

225 Vinkers CH, Kalafateli AL, Rutten BP, *et al.* Traumatic stress and human DNA methylation: a critical review. *Epigenomics* 2015; **7**: 593–608.

226 Cole SW, Hawkley LC, Arevalo JM, Sung CY, Rose RM, Cacioppo JT. Social regulation of gene expression in human leukocytes. *Genome biology* 2007; **8**: R189.

227 Wilson EO. Biophilia. Harvard University Press, 1984.

228 Lepczyk CA, Aronson MF, Evans KL, Goddard MA, Lerman SB, MacIvor JS. Biodiversity in the city: fundamental questions for understanding the ecology of urban green spaces for biodiversity conservation. *BioScience* 2017; **67**: 799–807.

229 Costanza R, d’Arge R, de Groot R, *et al.* The value of the world’s ecosystem services and natural capital. *Nature* 1997; **387**: 253–60.

230 Manes F, Marando F, Capotorti G, *et al.* Regulating ecosystem services of forests in ten Italian metropolitan cities: air quality improvement by PM10 and O3 removal. *Ecological Indicators* 2016; **67**: 425–440.

231 Hu Y, Wang D, Wei L, Zhang X, Song B. Bioaccumulation of heavy metals in plant leaves from Yan׳an city of the Loess Plateau, China. *Ecotoxicology and Environmental Safety* 2014; **110**: 82–8.

232 Gamper-Rabindran S, Timmins C. Hazardous Waste Cleanup, Neighborhood Gentrification, and Environmental Justice: Evidence from Restricted Access Census Block Data. *American Economic Review* 2011; **101**: 620–4.

233 Keesing F, Holt RD, Ostfeld RS. Effects of species diversity on disease risk. *Ecology letters* 2006; **9**: 485–498.

234 Hay SI, Tatem AJ, Graham AJ, Goetz SJ, Rogers DJ. Global environmental data for mapping infectious disease distribution. *Advances in parasitology* 2006; **62**: 37–77.

235 Krieger N. Theories for social epidemiology in the 21st century: an ecosocial perspective. *International journal of epidemiology* 2001; **30**: 668–677.

236 Zucchi FCR, Yao Y, Metz GAS. The secret language of destiny: stress imprinting and transgenerational origins of disease. *Front Genet* 2012; **3**. DOI:10.3389/fgene.2012.00096.

237 Stock JT. Are humans still evolving? *EMBO Rep* 2008; **9**: S51–4.

238 Griffiths AJ, Miller JH, Suzuki DT, Lewontin RC, Gelbart WM. Penetrance and expressivity. *An Introduction to Genetic Analysis 7th edition* 2000. https://www.ncbi.nlm.nih.gov/books/NBK22090/ (accessed Jan 20, 2020).

239 Barton N, Partridge L. Limits to natural selection. *BioEssays* 2000; **22**: 1075–84.

240 Berkman LF, Glass T, Brissette I, Seeman TE. From social integration to health: Durkheim in the new millennium. *Social science & medicine* 2000; **51**: 843–857.

241 Thibault A. Public and Civic Leisure in Quebec: Dynamic, Democratic, Passion-Driven, and Fragile. PUQ, 2008.

242 Triandis HC. Some Universals of Social Behavior. *Pers Soc Psychol Bull* 1978; **4**: 1–16.

243 Maddox GL. Disengagement theory: A critical evaluation. *The Gerontologist* 1964; **4**: 80–2, 103.

244 Rosenthal L, Levy SR. The relation between polyculturalism and intergroup attitudes among racially and ethnically diverse adults. *Cultural Diversity and Ethnic Minority Psychology* 2012; **18**: 1–16.

245 Moynihan M. Social Mimicry; Character Convergence Versus Character Displacement. *Evolution* 1968; **22**: 315–31.

246 Chartrand TL, Bargh JA. The chameleon effect: the perception-behavior link and social interaction. *J Pers Soc Psychol* 1999; **76**: 893–910.

247 Turner JC. Social influence. Belmont, CA, US: Thomson Brooks/Cole Publishing Co, 1991.

248 Christakis NA, Fowler JH. Social contagion theory: examining dynamic social networks and human behavior. *Statistics in Medicine* 2012; **43**: 556–77.

249 Triplett N. The dynamogenic factors in pacemaking and competition. *The American journal of psychology* 1898; **9**: 507–533.

250 Belletier C, Normand A, Huguet P. Social-Facilitation-and-Impairment Effects: From Motivation to Cognition and the Social Brain. *Curr Dir Psychol Sci* 2019; **28**: 260–5.

251 Bierhoff H-W. Prosocial Behaviour. Psychology Press, 2005 DOI:10.4324/9780203989425.

252 Fehr E, Fischbacher U. The nature of human altruism. *Nature* 2003; **425**: 785–91.

253 Cropanzano R, Mitchell MS. Social Exchange Theory: An Interdisciplinary Review. *Journal of Management* 2005; **31**: 874–900.

254 Maslow AH. A theory of human motivation. Simon and Schuster, 2013.

255 Doef MV der, Maes S. The Job Demand-Control (-Support) Model and psychological well-being: A review of 20 years of empirical research. *Work & Stress* 1999; **13**: 87–114.

256 Falk A, Fischbacher U. A theory of reciprocity. *Games and Economic Behavior* 2006; **54**: 293–315.

257 Roberts SG, Dunbar RI. Communication in social networks: Effects of kinship, network size, and emotional closeness. *Personal Relationships* 2011; **18**: 439–452.

258 Dunbar RIM. The Social Brain: Mind, Language, and Society in Evolutionary Perspective. *Annual Review of Anthropology* 2003; **32**: 163–81.

259 Carnelley KB, Ruscher JB. Adult Attachment and Exploratory Behavior in Leisure. *Journal of Social Behavior and Personality; Corte Madera, CA* 2000; **15**: 153–165.

260 Bretherton I. The origins of attachment theory: John Bowlby and Mary Ainsworth. *Developmental psychology* 1992; **28**: 759.

261 Weiss RS. Loneliness: The experience of emotional and social isolation. 1973.

262 Rokach A. Loneliness then and now: Reflections on social and emotional alienation in everyday life. *Current Psychology* 2004; **23**: 24–40.

263 Long CR, Averill JR. Solitude: An exploration of benefits of being alone. *Journal for the Theory of Social Behaviour* 2003; **33**: 21–44.

264 Yuval-Davis N. Belonging and the politics of belonging. *Patterns of Prejudice* 2006; **40**: 197–214.

265 Gilbert P. Defense, safe (ty) and biosocial goals in relation to the agonic and hedonic social modes. *World Futures: Journal of General Evolution* 1992; **35**: 31–70.

266 McFall RM. A review and reformulation of the concept of social skills. *Behavioral Assessment* 1982; **4**: 1–33.

267 Bos AER, Pryor JB, Reeder GD, Stutterheim SE. Stigma: Advances in Theory and Research. *Basic and Applied Social Psychology* 2013; **35**: 1–9.

268 Lukes S. Individualism. ECPR Press, 2006.

269 Welzel C. Freedom Rising: Human Empowerment And The Quest For Emancipation. Cambridge: Cambridge University Press, 2014 https://www.amazon.co.uk/Freedom-Rising-Human-Empowerment-Emancipation/dp/1107664837 (accessed Jan 2, 2020).

270 Earle TC, Cvetkovich G. Social Trust: Toward a Cosmopolitan Society. Greenwood Publishing Group, 1995.

271 Staub S, Green P. Psychology and Social Responsibility: Facing Global Challenges. NYU Press, 1992.

272 Goffman E. Frame analysis: An essay on the organization of experience. Harvard University Press, 1974.

273 Hong Y, Fang Y, Yang Y, Phua DY. Cultural Attachment: A New Theory and Method to Understand Cross-Cultural Competence. *Journal of Cross-Cultural Psychology* 2013; **44**: 1024–44.

274 Tomasello M, Kruger AC, Ratner HH. Cultural learning. *Behavioral and Brain Sciences* 1993; **16**: 495–511.

275 Ward C. The A, B, Cs of Acculturation. In: The handbook of culture and psychology. New York, NY, US: Oxford University Press, 2001: 411–45.

276 Berry JW. Acculturative Stress. In: Wong PTP, Wong LCJ, eds. Handbook of Multicultural Perspectives on Stress and Coping. Boston, MA: Springer US, 2006: 287–98.

277 Deardorff DK. Identification and Assessment of Intercultural Competence as a Student Outcome of Internationalization. *Journal of Studies in International Education* 2006; **10**: 241–66.

278 Csordas TJ. Embodiment and cultural phenomenology. na, 1999.

279 meeting A historical association A, Torpey P of SJ. Documenting Individual Identity: The Development of State Practices in the Modern World. Princeton University Press, 2001.

280 Hogg MA. Social identity theory. In: Burke PJ, ed. Contemporary Social Psychological Theories. Stanford University Press, 2006.

281 Heiss J. Social roles. In: Social psychology: Sociological perspectives. Piscataway, NJ, US: Transaction Publishers, 1990: 94–129.

282 Kralik D. The quest for ordinariness: transition experienced by midlife women living with chronic illness. *Journal of advanced nursing* 2002; **39**: 146–154.

283 Rogers KM, Ashforth BE. Respect in Organizations: Feeling Valued as “We” and “Me”. *Journal of Management* 2017; **43**: 1578–608.

284 Diener E, Fujita F. Social comparisons and subjective well-being. In: Health, coping, and well-being: Perspectives from social comparison theory. Mahwah, NJ, US: Lawrence Erlbaum Associates Publishers, 1997: 329–57.

285 Demakakos P, Nazroo J, Breeze E, Marmot M. Socioeconomic status and health: The role of subjective social status. *Social Science & Medicine* 2008; **67**: 330–40.

286 Krieger N, Williams DR, Moss NE. Measuring Social Class in US Public Health Research: Concepts, Methodologies, and Guidelines. *Annual Review of Public Health* 1997; **18**: 341–78.

287 Antonucci TC, Birditt KS, Akiyama H. Convoys of social relations: An interdisciplinary approach. In: Handbook of theories of aging, 2nd ed. New York, NY, US: Springer Publishing Co, 2009: 247–60.

288 Gould LJ, Ebers R, Clinchy RM. The Systems Psychodynamics of a Joint Venture: Anxiety Social Defenses, and the Management of Mutual Dependence. *Human Relations* 1999; **52**: 697–722.

289 Stern PC. Toward a social psychology of solidarity. *American Psychologist* 1986; **41**: 229–31.

290 Schwartz SH. Individualism-Collectivism: Critique and Proposed Refinements. *Journal of Cross-Cultural Psychology* 2016; published online July 26. DOI:10.1177/0022022190212001.

291 Stanley D. What Do We Know about Social Cohesion: The Research Perspective of the Federal Government’s Social Cohesion Research Network. *The Canadian Journal of Sociology / Cahiers canadiens de sociologie* 2003; **28**: 5–17.

292 Cantle T. Community Cohesion: A New Framework for Race and Diversity. Springer, 2018.

293 Williams KD. Ostracism: The Kiss of Social Death. *Social and Personality Psychology Compass* 2007; **1**: 236–47.

294 Jackson CL, Colquitt JA, Wesson MJ, Zapata-Phelan CP. Psychological collectivism: A measurement validation and linkage to group member performance. *Journal of Applied Psychology* 2006; **91**: 884–99.

295 Ferguson R, Gever M, Minh-Ha TT, González-Torres F, West C. Out There: Marginalization and Contemporary Cultures. MIT Press, 1990.

296 Kuran T, Sandholm WH. Cultural Integration and Its Discontents. *Rev Econ Stud* 2008; **75**: 201–28.

297 Goodenough WH. Multiculturalism as the Normal Human Experience. *Anthropology & Education Quarterly* 1976; **7**: 4–7.

298 Salemink O. Who decides who preserves what? Cultural preservation and cultural representation. *Vietnam’s cultural diversity: Approaches to preservation* 2001; : 205–226.

299 Rosenthal L, Levy SR. The colorblind, multicultural, and polycultural ideological approaches to improving intergroup attitudes and relations. *Social Issues and Policy Review* 2010; **4**: 215–46.

300 Adger WN. Social and ecological resilience: are they related? *Progress in Human Geography* 2000; **24**: 347–64.

301 Magis K. Community resilience: An indicator of social sustainability. *Society and Natural Resources* 2010; **23**: 401–416.

302 Turiel E, Wainryb C. Social Life in Cultures: Judgments, Conflict, and Subversion. *Child Development* 2000; **71**: 250–6.

303 Sidanius J, Pratto F. Social dominance: An intergroup theory of social hierarchy and oppression. Cambridge University Press, 2001.

304 Sorokin PA. Social mobility. Taylor & Francis, 1998.

305 Moore WE. Social Change, Foundations of Modern Sociology. Prentice Hall, Englewoods Cliffs, NJ, 1974.

306 Laverack G. Improving health outcomes through community empowerment: a review of the literature. *Journal of Health, Population and Nutrition* 2006; : 113–120.

307 Fiske ST, Berdahl J. Social power. *Social psychology: Handbook of basic principles* 2007; **2**: 678–692.

308 Lewens T. Cultural evolution: conceptual challenges. OUP Oxford, 2015.

309 Tyler T, Boeckmann RJ, Smith HJ, *et al.* Social Justice In A Diverse Society. Routledge, 2019 DOI:10.4324/9780429306310.

310 Williams RM. Relative deprivation. In: The idea of social structure. Routledge, 2017: 355–378.

311 Anderson RT, Sorlie P, Backlund E, Johnson N, Kaplan GA. Mortality Effects of Community Socioeconomic Status. *Epidemiology* 1997; **8**: 42–7.

312 Innes M, Jones V. Neighbourhood security and urban change: risk, resilience and recovery. 2006.

313 Marmot M. Income Inequality, Social Environment, and Inequalities in Health. *Journal of Policy Analysis and Management* 2001; **20**: 156–9.

314 Marmot MG. Understanding social inequalities in health. *Perspectives in biology and medicine* 2003; **46**: S9–S23.

315 Marmot M, Health C on SD of. Achieving health equity: from root causes to fair outcomes. *The Lancet* 2007; **370**: 1153–1163.

316 Cooke P, Propris LD. A policy agenda for EU smart growth: the role of creative and cultural industries. *Policy Studies* 2011; **32**: 365–75.

317 Weiermair K, Mathies C, Chon KS. The Tourism and Leisure Industry: Shaping the Future. Haworth Press, 2004.

318 Lorenz E, Valeyre A. Organisational Innovation, Human Resource Management and Labour Market Structure: A Comparison of the EU-15. *Journal of Industrial Relations* 2005; **47**: 424–42.

319 Marmot M. Social justice, epidemiology and health inequalities. *Eur J Epidemiol* 2017; **32**: 537–46.

320 Papies EK, Aarts H. Nonconscious self-regulation or the automatic pilot of human behavior. *Handbook of self-regulation: Research, theory, and applications* 2011; **2**: 125–142.

321 Verplanken B, Walker I, Davis A, Jurasek M. Context change and travel mode choice: Combining the habit discontinuity and self-activation hypotheses. *Journal of Environmental Psychology* 2008; **28**: 121–7.

322 Byng‐Hall J. The family script: a useful bridge between theory and practice. *Journal of Family Therapy* 1985; **7**: 301–5.

323 Samuelson W, Zeckhauser R. Status quo bias in decision making. *J Risk Uncertainty* 1988; **1**: 7–59.

324 Brehm SS, Brehm JW. Psychological reactance: A theory of freedom and control. Academic Press, 2013.

325 Skinner BF. The Selection of Behavior: The Operant Behaviorism of B. F. Skinner: Comments and Consequences. CUP Archive, 1988.

326 Clark RE. The classical origins of Pavlov’s conditioning. *Integr psych behav* 2004; **39**: 279–94.

327 Heckhausen H, Beckmann J. Intentional action and action slips. *Psychological Review* 1990; **97**: 36–48.

328 Lally P, Gardner B. Promoting habit formation. *Health Psychology Review* 2013; **7**: S137–58.

329 Tsaur S-H, Liang Y-W. Serious Leisure and Recreation Specialization. *Leisure Sciences* 2008; **30**: 325–41.

330 Atchley RC. A Continuity Theory of Normal Aging. *Gerontologist* 1989; **29**: 183–90.

331 Vallerand RJ, Blanchard C, Mageau GA, *et al.* Les passions de l’ame: on obsessive and harmonious passion. *Journal of personality and social psychology* 2003; **85**: 756.

332 Brehm JW, Cohen AR. Explorations in cognitive dissonance. Hoboken, NJ, US: John Wiley & Sons Inc, 1962 DOI:10.1037/11622-000.

333 Barkan R, Busemeyer JR. Changing plans: Dynamic inconsistency and the effect of experience on the reference point. *Psychonomic Bulletin & Review* 1999; **6**: 547–54.

334 Haselton MG, Nettle D, Murray DR. The Evolution of Cognitive Bias. In: The Handbook of Evolutionary Psychology. American Cancer Society, 2015: 1–20.

335 Edwards K, Smith EE. A disconfirmation bias in the evaluation of arguments. *Journal of Personality and Social Psychology* 1996; **71**: 5–24.

336 Von Winterfeldt D, Fischer GW. Multi-Attribute Utility Theory: Models and Assessment Procedures. In: Wendt D, Vlek C, eds. Utility, Probability, and Human Decision Making: Selected Proceedings of an Interdisciplinary Research Conference, Rome, 3–6 September, 1973. Dordrecht: Springer Netherlands, 1975: 47–85.

337 Reesor L, Vaughan EM, Hernandez DC, Johnston CA. Addressing Outcomes Expectancies in Behavior Change. *Am J Lifestyle Med* 2017; **11**: 430–2.

338 Fishbein M, Ajzen I. Belief, attitude, intention, and behavior: an introduction to theory and research. Addison-Wesley Pub. Co., 1975.

339 McIntyre C. Survival theory: tourist consumption as a beneficial experiential process in a limited risk setting. *International Journal of Tourism Research* 2007; **9**: 115–30.

340 Hartmann E, Harrison R, Zborowski M. Boundaries in the mind: Past research and future directions. *North American Journal of Psychology* 2001; **3**: 347–368.

341 Dinner I, Johnson EJ, Goldstein DG, Liu K. Partitioning default effects: Why people choose not to choose. *Journal of Experimental Psychology: Applied* 2011; **17**: 332–41.

342 Fershtman C, Seidmann DJ. Deadline Effects and Inefficient Delay in Bargaining with Endogenous Commitment. *Journal of Economic Theory* 1993; **60**: 306–21.

343 Cialdini RB, Kallgren CA, Reno RR. A Focus Theory of Normative Conduct: A Theoretical Refinement and Reevaluation of the Role of Norms in Human Behavior. 1991. DOI:10.1016/s0065-2601(08)60330-5.

344 Jones RA. Self-fulfilling prophecies: Social, psychological, and physiological effects of expectancies. Oxford, England: Lawrence Erlbaum Associates, 1977.

345 Fishbein M. An integrative model for behavioral prediction and its application to health promotion. In: Emerging theories in health promotion practice and research, 2nd ed. San Francisco, CA, US: Jossey-Bass, 2009: 215–34.

346 Mostofsky SH, Simmonds DJ. Response inhibition and response selection: two sides of the same coin. *J Cogn Neurosci* 2008; **20**: 751–61.

347 Logan GD, Schachar RJ, Tannock R. Impulsivity and Inhibitory Control. *Psychol Sci* 1997; **8**: 60–4.

348 Cummings S, Bridgman T, Brown KG. Unfreezing change as three steps: Rethinking Kurt Lewin’s legacy for change management. *Human Relations* 2016; **69**: 33–60.

349 Watts CE, Caldwell LL. Self-Determination and Free Time Activity Participation as Predictors of Initiative. *Journal of Leisure Research; Urbana* 2008; **40**: 156–81.

350 Locke EA, Latham GP. New Directions in Goal-Setting Theory. *Curr Dir Psychol Sci* 2006; **15**: 265–8.

351 Bandura A. Self-efficacy: Toward a unifying theory of behavioral change. *Psychological Review* 1977; **84**: 191–215.

352 Prochaska JO, Velicer WF. The Transtheoretical Model of Health Behavior Change. *Am J Health Promot* 1997; **12**: 38–48.

353 Taylor SE, Pham LB, Rivkin ID, Armor DA. Harnessing the imagination: Mental simulation, self-regulation, and coping. *American psychologist* 1998; **53**: 429.

354 Lowe R, Ziemke T. The Feeling of Action Tendencies: On the Emotional Regulation of Goal-Directed Behavior. *Front Psychol* 2011; **2**. DOI:10.3389/fpsyg.2011.00346.

355 Ryan RM, Deci EL. Self-determination theory and the facilitation of intrinsic motivation, social development, and well-being. *Am Psychol* 2000; **55**: 68–78.

356 Miller HL, Delgado PL, Salomon RM, et al. CLinical and biochemical effects of catecholamine depletion on antidepressant-induced remission of depression. *Arch Gen Psychiatry* 1996; **53**: 117–28.

357 Gürtler O, Münster J. Rational self-sabotage. *Mathematical Social Sciences* 2013; **65**: 1–4.

358 Goldberg YK, Eastwood JD, LaGuardia J, Danckert J. Boredom: An Emotional Experience Distinct from Apathy, Anhedonia, or Depression. *Journal of Social and Clinical Psychology* 2011; **30**: 647–66.

359 Ryan RM, Deci EL. Intrinsic and Extrinsic Motivations: Classic Definitions and New Directions. *Contemporary Educational Psychology* 2000; **25**: 54–67.

360 Seward JP. Drive, incentive, and reinforcement. *Psychological Review* 1956; **63**: 195–203.

361 Vroom VH. Motivation in management. American Foundation for Management Research, 1965.

362 Fried Y, Ferris GR. The Validity of the Job Characteristics Model: A Review and Meta-Analysis. *Personnel Psychology* 1987; **40**: 287–322.

363 Oyserman D. Identity-Based Motivation. In: Emerging Trends in the Social and Behavioral Sciences. American Cancer Society, 2015: 1–11.

364 Rothblum ED. Fear of Failure. In: Leitenberg H, ed. Handbook of Social and Evaluation Anxiety. Boston, MA: Springer US, 1990: 497–537.

365 Sandberg T, Conner M. Anticipated regret as an additional predictor in the theory of planned behaviour: A meta-analysis. *British Journal of Social Psychology* 2008; **47**: 589–606.

366 Cottrell NB. Social facilitation. *Experimental social psychology* 1972; : 185–236.

367 Gollwitzer PM. Implementation intentions: Strong effects of simple plans. *American Psychologist* 1999; **54**: 493–503.

368 Munns G, McFadden M. First Chance, Second Chance or Last Chance? Resistance and response to education. *British Journal of Sociology of Education* 2000; **21**: 59–75.

369 Berridge KC, Robinson TE. Parsing reward. *Trends in Neurosciences* 2003; **26**: 507–13.

370 Rogers T, Milkman KL, Volpp KG. Commitment Devices: Using Initiatives to Change Behavior. *JAMA* 2014; **311**: 2065–6.

371 Duckworth AL, Peterson C, Matthews MD, Kelly DR. Grit: Perseverance and passion for long-term goals. *Journal of Personality and Social Psychology* 2007; **92**: 1087–101.

372 Weinzimmer LG, Esken CA. Learning From Mistakes: How Mistake Tolerance Positively Affects Organizational Learning and Performance. *The Journal of Applied Behavioral Science* 2017; **53**: 322–48.

373 Zimmerman BJ, Kitsantas A. The Hidden Dimension of Personal Competence: Self-Regulated Learning and Practice. In: Handbook of competence and motivation. New York, NY, US: Guilford Publications, 2005: 509–26.

374 McAdams DP, Logan RL. What is generativity? In: The generative society: Caring for future generations. Washington, DC, US: American Psychological Association, 2004: 15–31.

375 McCallum SM, Rowe HJ, Gurrin L, Quinlivan JA, Rosenthal DA, Fisher JR. Unsettled infant behaviour and health service use: A cross-sectional community survey in Melbourne, Australia. *Journal of Paediatrics and Child Health* 2011; **47**: 818–823.

376 Walden TA, Ogan TA. The development of social referencing. *Child Dev* 1988; **59**: 1230–40.

377 Duncan GJ, Dowsett CJ, Claessens A, *et al.* School readiness and later achievement. *Developmental Psychology* 2007; **43**: 1428–46.

378 Reid K. Truancy and schools. Routledge, 2002.

379 Rose J, Roman N, Mwaba K, Ismail K. The relationship between parenting and internalizing behaviours of children: a systematic review. *Early Child Development and Care* 2018; **188**: 1468–86.

380 Millar S. The psychology of play. Oxford, England: Penguin Books, 1968.

381 Achenbach TM, Edelbrock CS. The classification of child psychopathology: A review and analysis of empirical efforts. *Psychological Bulletin* 1978; **85**: 1275–301.

382 Shonkoff JP. Building a new biodevelopmental framework to guide the future of early childhood policy. *Child Dev* 2010; **81**: 357–67.

383 Farrington DP. Understanding and Preventing Bullying. *Crime and Justice* 1993; **17**: 381–458.

384 Kon I. The Psychology of Independence. *International Journal of Sociology* 1987; **17**: 45–55.

385 Csikszentmihalyi M. Flow: The psychology of optimal performance. New York: Harper and Row, 1990.

386 Iso-Ahola SE, Dotson CO. Psychological momentum: Why success breeds success. *Review of General Psychology* 2014; **18**: 19–33.

387 Hodgson GM. The concept of a routine. *Handbook of organizational routines* 2008; **15**.

388 Putnam RD. Bowling alone: The collapse and revival of American community. Simon and Schuster, 2001.

389 Allan LG. The perception of time. *Perception & Psychophysics* 1979; **26**: 340–54.

390 O’Donoghue T, Rabin M. Doing it now or later. *American Economic Review* 1999; **89**: 103–124.

391 Gilbert DT, Wilson TD. Prospection: Experiencing the Future. *Science* 2007; **317**: 1351–4.

392 Buttimer A. Home, reach, and the sense of place. *The human experience of space and place* 1980; **3**: 166–187.

393 Arao B, Clemens K. From safe spaces to brave spaces. *The art of effective facilitation: Reflections from social justice educators* 2013; : 135–150.

394 Schroeder HW, Anderson LM. Perception of personal safety in urban recreation sites. *Journal of leisure research* 1984; **16**: 178–194.

395 Tuan Y-F. Space and Place: Humanistic Perspective. In: Gale S, Olsson G, eds. Philosophy in Geography. Dordrecht: Springer Netherlands, 1979: 387–427.

396 Barker RG. Ecological Psychology: Concepts and Methods for Studying the Environment of Human Behavior. Stanford University Press, 1968.

397 Ellegård K, Vilhelmson B. Home as a Pocket of Local Order: Everyday Activities and The Friction of Distance. *Geografiska Annaler: Series B, Human Geography* 2004; **86**: 281–96.

398 BUTTIMER A. Grasping the Dynamism of Lifeworld. *Annals of the Association of American Geographers* 1976; **66**: 277–92.

399 Cialdini RB. Influence. A. Michel Port Harcourt, 1987.

400 Rojek C. From Ritual Culture to Performative Culture. In: Leisure and Culture. Springer, 2000: 1–50.

401 Taylor S, Gonzaga G. Evolution, relationships, and health: The social shaping hypothesis. *Evolution and social psychology* 2006; : 211–236.

402 Coan JA, Sbarra DA. Social Baseline Theory: The Social Regulation of Risk and Effort. *Curr Opin Psychol* 2015; **1**: 87–91.

403 Sack RD. Human Territoriality: Its Theory and History. CUP Archive, 1986.

404 O’Reilly CA, Chatman JA. Culture as social control: Corporations, cults, and commitment. In: Research in organizational behavior: An annual series of analytical essays and critical reviews, Vol. 18. US: Elsevier Science/JAI Press, 1996: 157–200.

405 Agnew R. Revitalizing merton: General strain theory. In: The origins of American criminology. Routledge, 2017: 137–158.

406 Bandura A, Walters RH. Social learning theory. Prentice-hall Englewood Cliffs, NJ, 1977.

407 Boyd R, Richerson PJ, Henrich J. The cultural niche: Why social learning is essential for human adaptation. *PNAS* 2011; **108**: 10918–25.

408 Leibenstein H. Bandwagon, snob, and Veblen effects in the theory of consumers’ demand. *The quarterly journal of economics* 1950; **64**: 183–207.

409 Bouma J, Bulte E, van Soest D. Trust and cooperation: Social capital and community resource management. *Journal of Environmental Economics and Management* 2008; **56**: 155–66.

410 Dressler WW. Cultural consonance: Linking culture, the individual and health. *Preventive Medicine* 2012; **55**: 390.

411 Tomasello M, Rakoczy H. What Makes Human Cognition Unique? From Individual to Shared to Collective Intentionality. *Mind & Language* 2003; **18**: 121–47.

412 Bennett JW. CHAPTER 8 - Adaptation and Human Behavior. In: Bennett JW, ed. The Ecological Transition. Pergamon, 1976: 243–64.

413 Kearns A. Active citizenship and local governance: political and geographical dimensions. *Political Geography* 1995; **14**: 155–75.

414 van Zomeren M, Postmes T, Spears R. Toward an integrative social identity model of collective action: A quantitative research synthesis of three socio-psychological perspectives. *Psychological Bulletin* 2008; **134**: 504–35.

415 Drury J. Social Movements: A Social Psychological Perspective. In: Wright JD, ed. International Encyclopedia of the Social & Behavioral Sciences (Second Edition). Oxford: Elsevier, 2015: 447–53.

416 Bee C, Guerrina R. Framing Civic Engagement, Political Participation and Active Citizenship in Europe. Routledge, 2014 http://epubs.surrey.ac.uk/820637/ (accessed Jan 9, 2020).

417 Schachter O. Sharing the world’s resources. Columbia University Press, 1977.

418 Braha D. Global Civil Unrest: Contagion, Self-Organization, and Prediction. *PLOS ONE* 2012; **7**: e48596.

419 Rubin JZ, Pruitt DG, Kim SH. Social conflict: Escalation, stalemate, and settlement, 2nd ed. New York, NY, England: Mcgraw-Hill Book Company, 1994.

420 Deckers RJ, Eysenck HJ, Gudjonsson GH. The causes and cures of criminality. Springer Science & Business Media, 1989.

421 Barker RG, Gump PV. Big school, small school: High school size and student behavior. 1964.

422 Newton LH. Collective Responsibility in Healthcare. *J Med Philos* 1982; **7**: 11–22.

423 Meili R, O’Hearn S, Maeseneer JD, *et al.* How social accountability in medical education can contribute to global health. Global Health Experiential Education. 2017; published online Sept 14. DOI:10.4324/9781315107844-17.

424 Bandura A. Exercise of personal and collective efficacy in changing societies. *Self-efficacy in changing societies* 1995; **15**: 334.

425 Germov J, Freij M, Richmond K. A sociology of health promotion. 2014. https://nova.newcastle.edu.au/vital/access/manager/Repository/uon:18313 (accessed Jan 10, 2020).

426 Sallis JF, Owen N, Fisher E. Ecological models of health behavior. *Health behavior: Theory, research, and practice* 2015; **5**: 43–64.

427 Healey P, Stager ML, Woodmass K, *et al.* Cultural adaptations to augment health and mental health services: a systematic review. *BMC Health Services Research* 2017; **17**: 8.

428 Spracklen K, Lashua B, Sharpe E, Swain S. The Palgrave Handbook of Leisure Theory. Springer, 2017.

429 The Assets of Community Value (England) Regulations 2012. http://www.legislation.gov.uk/uksi/2012/2421/contents/made (accessed Jan 8, 2020).

430 Carter B, Grimwade G. Balancing use and preservation in cultural heritage management. *International Journal of Heritage Studies* 1997; **3**: 45–53.

431 Martin DG. “Place-Framing” as Place-Making: Constituting a Neighborhood for Organizing and Activism. *Annals of the Association of American Geographers* 2003; **93**: 730–50.

432 Dawson SL. Recruitment and retention of paraprofessionals. *Presentation to the Institute of Medicine’s Committee on the Future Healthcare Workforce for Older Americans Accessed April* 2007; **20**: 2009.

433 Nickel S, Trojan A, Kofahl C. Involving self-help groups in healthcare institutions: the patients’ contribution to and their view of ‘self-help friendliness’ as an approach to implement quality criteria of sustainable co-operation. *Health Expectations* 2017; **20**: 274–287.

434 Caldwell LL. Leisure and health: Why is leisure therapeutic? *British Journal of Guidance & Counselling* 2005; **33**: 7–26.

435 Drinkwater C, Wildman J, Moffatt S. Social prescribing. *BMJ* 2019; **364**: l1285.

436 Kelly JR. Socialization Toward Leisure: A Developmental Approach. *Journal of Leisure Research* 1974; **6**: 181–93.

437 Pate RR, Mitchell JA, Byun W, Dowda M. Sedentary behaviour in youth. *British journal of sports medicine* 2011; **45**: 906–913.

438 Mead MP, Irish LA. Application of health behaviour theory to sleep health improvement. *Journal of Sleep Research*; **n/a**: e12950.

439 Matarazzo JD. Behavioral immunogens and pathogens in health and illness. In: Hammonds BL, Scheirer CJ, eds. Psychology and health. Washington, DC, US: American Psychological Association, 1984: 9–43.

440 Floyd DL, Prentice‐Dunn S, Rogers RW. A Meta-Analysis of Research on Protection Motivation Theory. *Journal of Applied Social Psychology* 2000; **30**: 407–29.

441 Rosenstock IM. Historical Origins of the Health Belief Model: *Health Education Monographs* 1974; published online Dec 1. DOI:10.1177/109019817400200403.

442 Schwarzer R. Health action process approach (HAPA) as a theoretical framework to understand behavior change. *Actualidades en Psicología* 2016; **30**: 119–30.

443 Flay BR. Theory of Triadic Influence. In: Encyclopedia of Health and Behavior. Thousand Oaks: SAGE Publications, Inc., 2004: 715–8.

444 Petty RE, Cacioppo JT. The Elaboration Likelihood Model of Persuasion. In: Petty RE, Cacioppo JT, eds. Communication and Persuasion: Central and Peripheral Routes to Attitude Change. New York, NY: Springer, 1986: 1–24.

445 RYAN P. Integrated Theory of Health Behavior Change. *Clin Nurse Spec* 2009; **23**: 161–72.

446 Brown MT, Bussell JK. Medication Adherence: WHO Cares? *Mayo Clin Proc* 2011; **86**: 304–14.

447 Larimer ME, Palmer RS, Marlatt GA. Relapse prevention. An overview of Marlatt’s cognitive-behavioral model. *Alcohol Res Health* 1999; **23**: 151–60.

448 Weinstein ND. The precaution adoption process. *Health Psychol* 1988; **7**: 355–86.

449 Broese van Groenou MI, De Boer A. Providing informal care in a changing society. *Eur J Ageing* 2016; **13**: 271–9.

450 Michels MEJ, Evans DE, Blok GA. What is a clinical skill? Searching for order in chaos through a modified Delphi process. *Med Teach* 2012; **34**: e573-581.

451 Barker HR, Griffiths P, Mesa-Eguiagaray I, Pickering R, Gould L, Bridges J. Quantity and quality of interaction between staff and older patients in UK hospital wards: A descriptive study. *Int J Nurs Stud* 2016; **62**: 100–7.

452 Oyebode F. Clinical Errors and Medical Negligence. *MPP* 2013; **22**: 323–33.

453 Maslach C, Leiter MP. Burnout. *Stress and Quality of Working Life: Current Perspectives in Occupational Health* 2006; **37**: 42–49.

454 Zakay D. Post-decisional confidence and conflict experienced in a choice process. *Acta Psychologica* 1985; **58**: 75–80.

455 Rabia M, Knäuper B, Miquelon P. The eternal quest for optimal balance between maximizing pleasure and minimizing harm: The compensatory health beliefs model. *British Journal of Health Psychology* 2006; **11**: 139–53.

456 Jessor R. Problem-Behavior Theory, Psychosocial Development, and Adolescent Problem Drinking. *British Journal of Addiction* 1987; **82**: 331–42.

457 Hawkins JD, Weis JG. The social development model: An integrated approach to delinquency prevention. *J Primary Prevent* 1985; **6**: 73–97.

458 Kaplan HB, Martin SS, Robbins C. Application of a General Theory of Deviant Behavior: Self-Derogation and Adolescent Drug use. *Journal of Health and Social Behavior* 1982; **23**: 274–94.

459 Aguinaldo JP, Myers T. A Discursive Approach to Disinhibition Theory: The Normalization of Unsafe Sex Among Gay Men. *Qual Health Res* 2008; **18**: 167–81.

460 Buchanan LR, Rooks-Peck CR, Finnie RK, *et al.* Reducing recreational sedentary screen time: a community guide systematic review. *American journal of preventive medicine* 2016; **50**: 402–415.

461 Flay BR, Petraitis J, Hu FB. Psychosocial risk and protective factors for adolescent tobacco use. *Nicotine & tobacco research : official journal of the Society for Research on Nicotine and Tobacco* 1999; **1**: NaN-NaN.

462 Solomon RL. The opponent-process theory of acquired motivation: The costs of pleasure and the benefits of pain. *American Psychologist* 1980; **35**: 691–712.

463 Gesler WM. Healing Places. Rowman & Littlefield, 2003.

464 Taylor Salisbury T, Killaspy H, King M. The relationship between deinstitutionalization and quality of care in longer-term psychiatric and social care facilities in Europe: A cross-sectional study. *European Psychiatry* 2017; **42**: 95–102.

465 Bambs C, Reis SE. Embracing primordial prevention for ideal cardiovascular health. *Future Cardiology* 2011; **7**: 447–50.

466 Hornik R. Public Health Communication: Evidence for Behavior Change. Routledge, 2002.

467 Coulter A, Entwistle VA, Eccles A, Ryan S, Shepperd S, Perera R. Personalised care planning for adults with chronic or long-term health conditions. *Cochrane Database of Systematic Reviews* 2015.

468 Babalola O. Consumers and Their Demand for Healthcare. *Journal of Health & Medical Economics* 2017; **3**. DOI:10.21767/2471-9927.100032.

469 Goodman B. Erving Goffman and the total institution. *Nurse Educ Today* 2013; **33**: 81–2.

470 Shea S, Wynyard R, Lionis C. Providing Compassionate Healthcare: Challenges in Policy and Practice. Routledge, 2014.

471 Scott T, Mannion R, Davies H, *et al.* Healthcare Performance and Organisational Culture. CRC Press, 2018 DOI:10.1201/9781315376202.

472 Crawford P, Lewis L, Brown B, Manning N. Creative practice as mutual recovery in mental health. *Mental Health Review Journal* 2013; **18**: 55–64.

473 Cortis N. Overlooked and under-served? Promoting service use and engagement among ‘hard-to-reach’ populations. *International Journal of Social Welfare* 2012; **21**: 351–60.

474 Parrott R. Talking about Health: Why Communication Matters. John Wiley & Sons, 2009.

475 Krieger N, Chen JT, Waterman PD, *et al.* The inverse hazard law: blood pressure, sexual harassment, racial discrimination, workplace abuse and occupational exposures in US low-income black, white and Latino workers. *Soc Sci Med* 2008; **67**: 1970–81.

476 Cockerham WC. Health lifestyle theory and the convergence of agency and structure. *Journal of health and social behavior* 2005; **46**: 51–67.

477 Dingle GA, Williams E, Jetten J, Welch J. Choir singing and creative writing enhance emotion regulation in adults with chronic mental health conditions. *British Journal of Clinical Psychology* 2017; **56**: 443–57.

478 Kleinstäuber M, Reuter M, Doll N, Fallgatter AJ. Rock climbing and acute emotion regulation in patients with major depressive disorder in the context of a psychological inpatient treatment: a controlled pilot trial. *Psychology research and behavior management* 2017; **10**: 277–81.

479 Yamashita T, Bardo AR, Liu D. Experienced Subjective Well-Being During Physically Active and Passive Leisure Time Activities Among Adults Aged 65 Years and Older. *The Gerontologist* 2019; **59**: 718–26.

480 Fancourt D, Garnett C, Spiro N, West R, Müllensiefen D. How do artistic creative activities regulate our emotions? Validation of the Emotion Regulation Strategies for Artistic Creative Activities Scale (ERS-ACA). *PLOS ONE* 2019; **14**: e0211362.

481 Wang F, Orpana HM, Morrison H, de Groh M, Dai S, Luo W. Long-term Association Between Leisure-time Physical Activity and Changes in Happiness: Analysis of the Prospective National Population Health Survey. *American Journal of Epidemiology* 2012; **176**: 1095–100.

482 Kushnir J, Friedman A, Ehrenfeld M, Kushnir T. Coping with Preoperative Anxiety in Cesarean Section: Physiological, Cognitive, and Emotional Effects of Listening to Favorite Music. *Birth* 2012; **39**: 121–7.

483 Buecker S, Simacek T, Ingwersen B, Terwiel S, Simonsmeier BA. Physical activity and subjective well-being in healthy individuals: a meta-analytic review. *Health Psychology Review* 2020; : 1–19.

484 Wiese CW, Kuykendall L, Tay L. Get active? A meta-analysis of leisure-time physical activity and subjective well-being. *Journal of Positive Psychology* 2018; **13**: 57–66.

485 Kuykendall L, Tay L, Ng V. Leisure engagement and subjective well-being: A meta-analysis. *Psychological Bulletin* 2015; **141**: 364–403.

486 de Witte M, Spruit A, van Hooren S, Moonen X, Stams G-J. Effects of music interventions on stress-related outcomes: a systematic review and two meta-analyses. *Health Psychology Review* 2019; : 1–31.

487 Panteleeva Y, Ceschi G, Glowinski D, Courvoisier DS, Grandjean D. Music for anxiety? Meta-analysis of anxiety reduction in non-clinical samples. *Psychology of Music* 2017; **46**: 473–87.

488 Stubbs B, Vancampfort D, Rosenbaum S, *et al.* An examination of the anxiolytic effects of exercise for people with anxiety and stress-related disorders: A meta-analysis. *Psychiatry research* 2017; **249**: 102–8.

489 Aldao A, Nolen-Hoeksema S, Schweizer S. Emotion-regulation strategies across psychopathology: A meta-analytic review. *Clin Psychol Rev* 2010; **30**: 217–37.

490 Lewis M, Haviland-Jones JM, Barrett LF. Handbook of Emotions. Guilford Press, 2010.

491 Steptoe A, Wardle J, Marmot M. Positive affect and health-related neuroendocrine, cardiovascular, and inflammatory processes. *PNAS* 2005; **102**: 6508–12.

492 Çulhacik GD, Durat G, Eren N. Effects of activity groups, in which art activities are used, on resilience and related factors in families with disabled children. *Perspectives in Psychiatric Care* 2020; **n/a**. DOI:10.1111/ppc.12569.

493 Fancourt D, Perkins R, Ascenso S, Carvalho LA, Steptoe A, Williamon A. Effects of Group Drumming Interventions on Anxiety, Depression, Social Resilience and Inflammatory Immune Response among Mental Health Service Users. *PloS one* 2016; **11**: e0151136–e0151136.

494 Wong PKS, Fong KW, Lam TL. Enhancing the Resilience of Parents of Adults With Intellectual Disabilities through Volunteering: An Exploratory Study. *Journal of Policy and Practice in Intellectual Disabilities* 2015; **12**: 20–6.

495 Cocozza S, Sacco PL, Matarese G, Maffulli GD, Maffulli N, Tramontano D. Participation to Leisure Activities and Well-Being in a Group of Residents of Naples-Italy: The Role of Resilience. *International journal of environmental research and public health* 2020; **17**: 1895.

496 Diamond S, Shrira A. Psychological vulnerability and resilience of Holocaust survivors engaged in creative art. *Psychiatry Research* 2018; **264**: 236–43.

497 Román-Mata SS, Puertas-Molero P, Ubago-Jiménez JL, González-Valero G. Benefits of physical activity and its associations with resilience, emotional intelligence, and psychological distress in university students from southern spain. *International Journal of Environmental Research and Public Health* 2020; **17**: 1–12.

498 Llenares II, Sario JA, Bialba D, Dela Cruz JM. Volunteerism influences on student resilience and gratitude. *Journal of Psychology in Africa* 2020; **30**: 211–6.

499 Wang C, Schmid CH, Fielding RA, *et al.* Effect of tai chi versus aerobic exercise for fibromyalgia: comparative effectiveness randomized controlled trial. *BMJ (Clinical research ed)* 2018; **360**: k851.

500 ÖSTER I, SVENSK A-C, MAGNUSSON EVA, *et al.* Art therapy improves coping resources: A randomized, controlled study among women with breast cancer. *Palliative and Supportive Care* 2006; **4**: 57–64.

501 Robb SL, Burns DS, Stegenga KA, *et al.* Randomized clinical trial of therapeutic music video intervention for resilience outcomes in adolescents/young adults undergoing hematopoietic stem cell transplant: A report from the Children’s Oncology Group. *Cancer* 2014; **120**: 909–17.

502 Bygren LO, Weissglas G, Wikström B-M, *et al.* Cultural Participation and Health: A Randomized Controlled Trial Among Medical Care Staff. *Psychosomatic Medicine* 2009; **71**.

503 Kovács E, Prókai L, Mészáros L, Gondos T. Adapted physical activity is beneficial on balance, functional mobility, quality of life and fall risk in community-dwelling older women: a randomized single-blinded controlled trial. *European journal of physical and rehabilitation medicine* 2013; **49**: 301–10.

504 Sivaramakrishnan D, Fitzsimons C, Kelly P, *et al.* The effects of yoga compared to active and inactive controls on physical function and health related quality of life in older adults- systematic review and meta-analysis of randomised controlled trials. *The international journal of behavioral nutrition and physical activity* 2019; **16**: 33.

505 Cugusi L, Prosperini L, Mura G. Exergaming for Quality of Life in persons living with chronic diseases: A systematic review and meta-analysis. *PM&R* 2020; **n/a**. DOI:10.1002/pmrj.12444.

506 Pisu M, Demark-Wahnefried W, Kenzik KM, *et al.* A dance intervention for cancer survivors and their partners (RHYTHM). *Journal of cancer survivorship : research and practice* 2017; **11**: 350–9.

507 Davydov DM, Stewart R, Ritchie K, Chaudieu I. Resilience and mental health. *Clinical Psychology Review* 2010; **30**: 479–95.

508 Folkman S, Lazarus RS, Gruen RJ, DeLongis A. Appraisal, coping, health status, and psychological symptoms. *Journal of personality and social psychology* 1986; **50**: 571.

509 Franklin TB, Saab BJ, Mansuy IM. Neural Mechanisms of Stress Resilience and Vulnerability. *Neuron* 2012; **75**: 747–61.

510 Haglund MEM, Nestadt PS, Cooper NS, Southwick SM, Charney DS. Psychobiological mechanisms of resilience: Relevance to prevention and treatment of stress-related psychopathology. *Development and Psychopathology* 2007; **19**: 889–920.

511 Russo SJ, Murrough JW, Han M-H, Charney DS, Nestler EJ. Neurobiology of resilience. *Nature Neuroscience* 2012; **15**: 1475–84.

512 Schwender TM, Spengler S, Oedl C, Mess F. Effects of Dance Interventions on Aspects of the Participants’ Self: A Systematic Review. *Frontiers in psychology* 2018; **9**: 1130.

513 Dunphy K, Baker FA, Dumaresq E, *et al.* Creative Arts Interventions to Address Depression in Older Adults: A Systematic Review of Outcomes, Processes, and Mechanisms . Frontiers in Psychology . 2019; **9**: 2655.

514 Bungay H, Vella-Burrows T. The effects of participating in creative activities on the health and well-being of children and young people: a rapid review of the literature. *Perspectives in Public Health* 2013; **133**: 44–52.

515 García-Martínez AM, De Paz JA, Márquez S. Effects of an exercise programme on self-esteem, self-concept and quality of life in women with fibromyalgia: a randomized controlled trial. *Rheumatology International* 2012; **32**: 1869–76.

516 Legrand FD. Effects of Exercise on Physical Self-Concept, Global Self-Esteem, and Depression in Women of Low Socioeconomic Status With Elevated Depressive Symptoms. *Journal of Sport and Exercise Psychology* 2014; **36**: 357–65.

517 Chang B-H, Chen B-W, Beckstead JW, Yang C-Y. Effects of a music-creation programme on the anxiety, self-esteem, and quality of life of people with severe mental illness: A quasi-experimental design. *International journal of mental health nursing* 2018; **27**: 1066–76.

518 Porter S, McConnell T, McLaughlin K, *et al.* Music therapy for children and adolescents with behavioural and emotional problems: a randomised controlled trial. *Journal of Child Psychology and Psychiatry* 2017; **58**: 586–94.

519 Liu M, Wu L, Ming Q. How Does Physical Activity Intervention Improve Self-Esteem and Self-Concept in Children and Adolescents? Evidence from a Meta-Analysis. *PloS one* 2015; **10**: e0134804.

520 Spruit A, Assink M, van Vugt E, van der Put C, Stams GJ. The effects of physical activity interventions on psychosocial outcomes in adolescents: A meta-analytic review. *Clinical psychology review* 2016; **45**: 56–71.

521 Babic MJ, Morgan PJ, Plotnikoff RC, Lonsdale C, White RL, Lubans DR. Physical activity and physical self-concept in youth: systematic review and meta-analysis. *Sports medicine (Auckland, NZ)* 2014; **44**: 1589–601.

522 Bernard ME. The strength of self-acceptance: Theory, practice and research. Springer, 2014.

523 Burke PJ, Owens TJ, Serpe RT, Thoits PA. Advances in identity theory and research. Springer, 2003.

524 Haslam C, Jetten J, Alexander SH. The social cure: Identity, health and well-being. Psychology press, 2012.

525 Kaptein AA, Meulenberg F, Smyth JM. A breath of fresh air: images of respiratory illness in novels, poems, films, music, and paintings. *Journal of health psychology* 2015; **20**: 246—258.

526 Greenhalgh T. Cultural contexts of health: the use of narrative research in the health sector. 2016.

527 Cheung MMY, Saini B, Smith L. Using drawings to explore patients’ perceptions of their illness: a scoping review. *Journal of multidisciplinary healthcare* 2016; **9**: 631–46.

528 Broadbent E, Schoones JW, Tiemensma J, Kaptein AA. A systematic review of patients’ drawing of illness: implications for research using the Common Sense Model. *Health Psychology Review* 2019; **13**: 406–26.

529 Kabel A, Teti M, Zhang N. The art of resilience: photo-stories of inspiration and strength among people with HIV/AIDS. *Visual Studies* 2016; **31**: 221–30.

530 Michalak EE, Livingston JD, Maxwell V, Hole R, Hawke LD, Parikh S V. Using theatre to address mental illness stigma: a knowledge translation study in bipolar disorder. *International Journal of Bipolar Disorders* 2014; **2**: 1.

531 Morgan AL, Tobar DA, Snyder L. Walking toward a new me: the impact of prescribed walking 10,000 steps/day on physical and psychological well-being. *Journal of physical activity & health* 2010; **7**: 299–307.

532 Joseph RP, Pekmezi D, Dutton GR, *et al.* Results of a Culturally Adapted Internet-Enhanced Physical Activity Pilot Intervention for Overweight and Obese Young Adult African American Women. *Journal of transcultural nursing : official journal of the Transcultural Nursing Society* 2016; **27**: 136–46.

533 Compton WC. Toward a Tripartite Factor Structure of Mental Health: Subjective Well-Being, Personal Growth, and Religiosity. *The Journal of Psychology* 2001; **135**: 486–500.

534 Hampson SE, Goldberg LR, Vogt TM, Dubanoski JP. Mechanisms by which childhood personality traits influence adult health status: Educational attainment and healthy behaviors. *Health Psychology* 2007; **26**: 121–5.

535 Kearney MH, O’Sullivan J. Identity Shifts as Turning Points in Health Behavior Change. *West J Nurs Res* 2003; **25**: 134–52.

536 Robitschek C, Keyes CLM. Keyes’s model of mental health with personal growth initiative as a parsimonious predictor. *Journal of Counseling Psychology* 2009; **56**: 321–9.

537 VanderWeele TJ. On the promotion of human flourishing. *Proceedings of the National Academy of Sciences* 2017; **114**: 8148 LP – 8156.

538 Anderson LS, Heyne LA. Flourishing through Leisure: An Ecological Extension of the Leisure and Well-Being Model in Therapeutic Recreation Strengths-Based Practice. *Therapeutic Recreation Journal; Vol 46, No 2 (2012)* 2012.

539 Tay L, Pawelski JO, Keith MG. The role of the arts and humanities in human flourishing: A conceptual model. *Journal of Positive Psychology* 2018; **13**: 215–25.

540 Steptoe A, Fancourt D. Leading a meaningful life at older ages and its relationship with social engagement, prosperity, health, biology, and time use. *Proceedings of the National Academy of Sciences* 2019; **116**: 1207 LP – 1212.

541 Iwasaki Y. Contributions of leisure to “meaning-making” and its implications for leisure studies and services. *Annals of Leisure Research* 2017; **20**: 416–26.

542 Morgan GS, Willmott M, Ben-Shlomo Y, Haase AM, Campbell RM. A life fulfilled: positively influencing physical activity in older adults - a systematic review and meta-ethnography. *BMC public health* 2019; **19**: 362.

543 Iwasaki Y, Messina ES, Hopper T. The role of leisure in meaning-making and engagement with life. *The Journal of Positive Psychology* 2018; **13**: 29–35.

544 Iwasaki Y. Pathways to meaning-making through leisure-like pursuits in global contexts. *Journal of Leisure Research* 2008; **40**: 231–49.

545 Rodriguez-Ayllon M, Cadenas-Sánchez C, Estévez-López F, *et al.* Role of Physical Activity and Sedentary Behavior in the Mental Health of Preschoolers, Children and Adolescents: A Systematic Review and Meta-Analysis. *Sports Medicine* 2019; **49**: 1383–410.

546 Jenkinson CE, Dickens AP, Jones K, *et al.* Is volunteering a public health intervention? A systematic review and meta-analysis of the health and survival of volunteers. *BMC public health* 2013; **13**: 773.

547 Cruz-Ferreira A, Marmeleira J, Formigo A, Gomes D, Fernandes J. Creative Dance Improves Physical Fitness and Life Satisfaction in Older Women. *Research on Aging* 2015; **37**: 837–55.

548 Ong AD, Patterson A. Eudaimonia, Aging, and Health: A Review of Underlying Mechanisms. In: Vittersø J, ed. Handbook of Eudaimonic Well-Being. Cham: Springer International Publishing, 2016: 371–8.

549 Ryff CD. Psychological Well-Being Revisited: Advances in the Science and Practice of Eudaimonia. *PPS* 2014; **83**: 10–28.

550 Steptoe A, Deaton A, Stone AA. Subjective wellbeing, health, and ageing. *The Lancet* 2015; **385**: 640–8.

551 Steptoe A, Fancourt D. Leading a meaningful life at older ages and its relationship with social engagement, prosperity, health, biology, and time use. *Proceedings of the National Academy of Sciences* 2019; **116**: 1207–1212.

552 Stone A, Mackie C, editors. Subjective Well-Being: Measuring Happiness, Suffering, and Other Dimensions of Experience. Washington, DC: National Academies Press (US), 2013 DOI:10.17226/18548.

553 Fancourt D, Steptoe A, Cadar D. Cultural engagement and cognitive reserve: Museum attendance and dementia incidence over a 10-year period. *British Journal of Psychiatry* 2018; **213**: 661–3.

554 Fancourt D, Steptoe A, Cadar D. Community engagement and dementia risk: Time-to-event analyses from a national cohort study. *Journal of Epidemiology and Community Health* 2020; **74**: 71–7.

555 Fancourt D, Steptoe A. Cultural engagement predicts changes in cognitive function in older adults over a 10 year period: Findings from the English Longitudinal Study of Ageing. *Scientific Reports* 2018; **8**. DOI:10.1038/s41598-018-28591-8.

556 Cadar D, Hackett RA, Mischie M, Llewellyn DJ, Batty GD, Steptoe A. Association of physical activity as a distinctive feature of clustering of lifestyle behaviours with dementia risk: evidence from the English Longitudinal Study of Ageing. *The Lancet* 2017; **390**: S29.

557 Gheysen F, Poppe L, DeSmet A, *et al.* Physical activity to improve cognition in older adults: can physical activity programs enriched with cognitive challenges enhance the effects? A systematic review and meta-analysis. *International Journal of Behavioral Nutrition and Physical Activity* 2018; **15**: 63.

558 Sanders LMJ, Hortobágyi T, la Bastide-van Gemert S, van der Zee EA, van Heuvelen MJG. Dose-response relationship between exercise and cognitive function in older adults with and without cognitive impairment: A systematic review and meta-analysis. *PLOS ONE* 2019; **14**: e0210036.

559 Falck RS, Davis JC, Best JR, Crockett RA, Liu-Ambrose T. Impact of exercise training on physical and cognitive function among older adults: a systematic review and meta-analysis. *Neurobiology of Aging* 2019; **79**: 119–30.

560 Bollimbala A, James PS, Ganguli S. Impact of Acute Physical Activity on Children’s Divergent and Convergent Thinking: The Mediating Role of a Low Body Mass Index. *Perceptual and Motor Skills* 2019; **126**: 603–22.

561 He W-J, Wong W-C, Hui AN-N. Emotional Reactions Mediate the Effect of Music Listening on Creative Thinking: Perspective of the Arousal-and-Mood Hypothesis . Frontiers in Psychology . 2017; **8**: 1680.

562 Ritter SM, Ferguson S. Happy creativity: Listening to happy music facilitates divergent thinking. *PLOS ONE* 2017; **12**: e0182210.

563 Barulli D, Stern Y. Efficiency, capacity, compensation, maintenance, plasticity: emerging concepts in cognitive reserve. *Trends in Cognitive Sciences* 2013; **17**: 502–9.

564 Craik FIM, Bialystok E. Cognition through the lifespan: mechanisms of change. *Trends in Cognitive Sciences* 2006; **10**: 131–8.

565 Gonzalez-Liencres C, Shamay-Tsoory SG, Brüne M. Towards a neuroscience of empathy: Ontogeny, phylogeny, brain mechanisms, context and psychopathology. *Neuroscience & Biobehavioral Reviews* 2013; **37**: 1537–48.

566 Heilman KM. Possible Brain Mechanisms of Creativity. *Arch Clin Neuropsychol* 2016; **31**: 285–96.

567 Weare K. Mental Health and Social and Emotional Learning: Evidence, Principles, Tensions, Balances. *Advances in School Mental Health Promotion* 2010; **3**: 5–17.

568 Koh E, Shrimpton B. Art promoting mental health literacy and a positive attitude towards people with experience of mental illness. *The International journal of social psychiatry* 2014; **60**: 169–74.

569 Biglino G, Layton S, Lee M, Sophocleous F, Hall S, Wray J. ‘Making the Invisible Visible’: an audience response to an art installation representing the complexity of congenital heart disease and heart transplantation. *Medical humanities* 2019; **45**: 399–405.

570 Harris MW, Barnett T, Bridgman H. Rural Art Roadshow: a travelling art exhibition to promote mental health in rural and remote communities. *Arts & Health* 2018; **10**: 57–64.

571 Schachter SC. Epilepsy and art: Windows into complexity and comorbidities. *Epilepsy &amp; behavior : E&amp;B* 2016; **57**: 265–9.

572 Nyamathi A, Slagle A, Thomas A, *et al.* Art messaging to engage homeless young adults. *Progress in community health partnerships : research, education, and action* 2011; **5**: 9–18.

573 Rosenberg M, Ferguson R. Maintaining relevance: an evaluation of health message sponsorship at Australian community sport and arts events. *BMC Public Health* 2014; **14**: 1242.

574 Orozco-Olvera V, Shen F, Cluver L. The effectiveness of using entertainment education narratives to promote safer sexual behaviors of youth: A meta-analysis, 1985-2017. *PLOS ONE* 2019; **14**: e0209969.

575 Glover TD, Hemingway JL. Locating Leisure in the Social Capital Literature. *Journal of Leisure Research* 2005; **37**: 387–401.

576 Blackshaw T, Long J. What’s the Big Idea? A Critical Exploration of the Concept of Social Capital and its Incorporation into Leisure Policy Discourse. *Leisure Studies* 2005; **24**: 239–58.

577 Warde A. Cultural capital and the place of sport. *Cultural Trends* 2006; **15**: 107–22.

578 Stalker GJ. Leisure Diversity as an Indicator of Cultural Capital. *Leisure Sciences* 2011; **33**: 81–102.

579 Horolets A, Stodolska M, Peters K. Natural Environments and Leisure among Rural–to–Urban Immigrants: An Application of Bourdieu’s Concepts of Habitus, Social and Cultural Capital, and Field. *Leisure Sciences* 2019; **41**: 313–29.

580 Osborn CY, Paasche-Orlow MK, Bailey SC, Wolf MS. The Mechanisms Linking Health Literacy to Behavior and Health Status. 2011; published online Jan. DOI:info:doi/10.5993/AJHB.35.1.11.

581 Shim JK. Cultural Health Capital: A Theoretical Approach to Understanding Healthcare Interactions and the Dynamics of Unequal Treatment. *J Health Soc Behav* 2010; **51**: 1–15.

582 Kouhia A. Crafting the collective sense: A descriptive case study on recreational textile craft-making in Finnish adult education. *International Journal of Education Through Art* 2015; **11**: 7–20.

583 HYLTON CLA. African-Caribbean group activities, individual and collective consciousness, and enforced ‘leisure’. *Community, Work & Family* 2003; **6**: 103–13.

584 Chick G, Hsu Y-C, Yeh C-K, Hsieh C-M, Bae SY, Iarmolenko S. Cultural Consonance in Leisure, Leisure Satisfaction, Life Satisfaction, and Self-Rated Health in Urban Taiwan. *Leisure Sciences* 2016; **38**: 402–23.

585 Andrieu B, Parry J, Porrovecchio A, Sirost O. Body ecology and emersive leisure. 2018 DOI:10.4324/9780203704059.

586 Stevenson N, Farrell H. Taking a hike: exploring leisure walkers embodied experiences . *Social and Cultural Geography* 2018; **19**: 429–47.

587 Frith CD, Frith U. Mechanisms of Social Cognition. *Annual Review of Psychology* 2012; **63**: 287–313.

588 Hilbert RA. Durkheim and Merton on Anomie: An Unexplored Contrast and Its Derivatives. *Soc Probl* 1989; **36**: 242–50.

589 Kandori M. Social Norms and Community Enforcement. *Rev Econ Stud* 1992; **59**: 63–80.

590 Cockrill K, Biggs A. Can stories reduce abortion stigma? Findings from a longitudinal cohort study. *Culture, health & sexuality* 2018; **20**: 335–50.

591 Twardzicki M. Challenging stigma around mental illness and promoting social inclusion using the performing arts. *The journal of the Royal Society for the Promotion of Health* 2008; **128**: 68–72.

592 Logie CH, Dias L V, Jenkinson J, *et al.* Exploring the Potential of Participatory Theatre to Reduce Stigma and Promote Health Equity for Lesbian, Gay, Bisexual, and Transgender (LGBT) People in Swaziland and Lesotho. *Health education & behavior : the official publication of the Society for Public Health Education* 2019; **46**: 146–56.

593 Flanagan EH, Buck T, Gamble A, Hunter C, Sewell I, Davidson L. ‘Recovery Speaks’: A Photovoice Intervention to Reduce Stigma Among Primary Care Providers. *Psychiatric services (Washington, DC)* 2016; **67**: 566–9.

594 Karnik M, Printz B, Finkel J. A Hospital’s Contemporary Art Collection: Effects on Patient Mood, Stress, Comfort, and Expectations. *HERD: Health Environments Research & Design Journal* 2014; **7**: 60–77.

595 van der Wal-Huisman H, Dons KSK, Smilde R, Heineman E, van Leeuwen BL. The effect of music on postoperative recovery in older patients: A systematic review. *Journal of geriatric oncology* 2018; **9**: 550–9.

596 Slater JK, Braverman MT, Meath T. Patient satisfaction with a hospital’s arts-enhanced environment as a predictor of the likelihood of recommending the hospital. *Arts & Health* 2017; **9**: 97–110.

597 Teksoz E, Bilgin I, Madzwamuse SE, Oscakci AF. The impact of a creative play intervention on satisfaction with nursing care: A mixed-methods study. *Journal for Specialists in Pediatric Nursing* 2017; **22**: e12169.

598 Kyriakides R, Jones P, Geraghty R, *et al.* Effect of Music on Outpatient Urological Procedures: A Systematic Review and Meta-Analysis from the European Association of Urology Section of Uro-Technology. *The Journal of Urology* 2018; **199**: 1319–27.

599 Barker KK. The social construction of illness. *Handbook of medical sociology* 2010; : 147–62.

600 Chaudoir SR, Earnshaw VA, Andel S. “Discredited” Versus “Discreditable”: Understanding How Shared and Unique Stigma Mechanisms Affect Psychological and Physical Health Disparities. *Basic and Applied Social Psychology* 2013; **35**: 75–87.

601 Hatzenbuehler ML, Phelan JC, Link BG. Stigma as a Fundamental Cause of Population Health Inequalities. *Am J Public Health* 2013; **103**: 813–21.

602 Link BG. Stigma: many mechanisms require multifaceted responses. *Epidemiology and Psychiatric Sciences* 2001; **10**: 8–11.

603 Heider A, Warner RS. Bodies in sync: Interaction ritual theory applied to sacred harp singing. *Sociology of Religion: A Quarterly Review* 2010; **71**: 76–97.

604 Plancke C. Affect, creativity, and community-making in a Congolese song-dance performance: or how to follow the movement of the social. *Journal of the Royal Anthropological Institute* 2014; **20**: 653–69.

605 van der Vaart G, van Hoven B, Huigen PPP. The value of participatory community arts for community resilience. In: Governing for Resilience in Vulnerable Places. 2017: 186–204.

606 Bryant LO. Music, Memory, and Nostalgia: Collective Memories of Cultural Revolution Songs in Contemporary China. *China Review* 2005; **5**: 151–75.

607 Levang LE, Bye TA, Hirrich A, Røkkum NHA, Torp TU, Tjora A. The collective effervescence of the music festival . *Tidsskrift for Samfunnsforskning* 2017; **58**: 62–83.

608 Bowness J, Tulle E, McKendrick J. Understanding the parkrun community; sacred Saturdays and organic solidarity of parkrunners. *European Journal for Sport and Society* 2020; : 1–20.

609 Escueta M, Butterwick S. The power of popular education and visual arts for trauma survivors’ critical consciousness and collective action. *International Journal of Lifelong Education* 2012; **31**: 325–40.

610 Berry DS, Pennebaker JW. Nonverbal and Verbal Emotional Expression and Health. *PPS* 1993; **59**: 11–9.

611 Luszczynska A, Benight CC, Cieslak R. Self-Efficacy and Health-Related Outcomes of Collective Trauma. *European Psychologist* 2009; **14**: 51–62.

612 Szanto T. Collective Emotions, Normativity, and Empathy: A Steinian Account. *Hum Stud* 2015; **38**: 503–27.

613 Colcombe SJ, Erickson KI, Scalf PE, *et al.* Aerobic Exercise Training Increases Brain Volume in Aging Humans. *J Gerontol A Biol Sci Med Sci* 2006; **61**: 1166–70.

614 Desbordes G, Negi LT, Pace TWW, Wallace BA, Raison CL, Schwartz EL. Effects of mindful-attention and compassion meditation training on amygdala response to emotional stimuli in an ordinary, non-meditative state. *Front Hum Neurosci* 2012; **6**. DOI:10.3389/fnhum.2012.00292.

615 Pacchetti C, Mancini F, Aglieri R, Fundarò C, Martignoni E, Nappi G. Active music therapy in Parkinson’s disease: an integrative method for motor and emotional rehabilitation. *Psychosom Med* 2000; **62**: 386–93.

616 Beaty RE, Kenett YN, Christensen AP, *et al.* Robust prediction of individual creative ability from brain functional connectivity. *PNAS* 2018; **115**: 1087–92.

617 Jung RE, Segall JM, Jeremy Bockholt H, *et al.* Neuroanatomy of creativity. *Hum Brain Mapp* 2009; **31**: 398–409.

618 Karpati FJ, Giacosa C, Foster NEV, Penhune VB, Hyde KL. Dance and the brain: a review: Dance and the brain: a review. *Ann NY Acad Sci* 2015; **1337**: 140–6.

619 Ueno K, Takahashi T, Takahashi K, Mizukami K, Tanaka Y, Wada Y. Neurophysiological basis of creativity in healthy elderly people: A multiscale entropy approach. *Clinical Neurophysiology* 2015; **126**: 524–31.

620 Boso M, Politi P, Barale F, Enzo E. Neurophysiology and neurobiology of the musical experience. *Funct Neurol* 2006; **21**: 187–91.

621 Koelsch S. Brain correlates of music-evoked emotions. *Nature Reviews Neuroscience* 2014; **15**: 170.

622 Cohen G. New theories and research findings on the positive influence of music and art on health with ageing. *Arts & Health* 2009; **1**: 48–62.

623 Eisenberger NI, Cole SW. Social neuroscience and health: neurophysiological mechanisms linking social ties with physical health. *Nature neuroscience* 2012; **15**: 669.

624 Gligoroska JP, Manchevska S. The Effect of Physical Activity on Cognition – Physiological Mechanisms. *Mater Sociomed* 2012; **24**: 198–202.

625 Lubans D, Richards J, Hillman C, *et al.* Physical Activity for Cognitive and Mental Health in Youth: A Systematic Review of Mechanisms. *Pediatrics* 2016; **138**. DOI:10.1542/peds.2016-1642.

626 Martindale C, Locher P, Petrov VM, Berleant A. Evolutionary and Neurocognitive Approaches to Aesthetics, Creativity and the Arts. Routledge, 2019.

627 Seybold KS. Physiological Mechanisms Involved in Religiosity/Spirituality and Health. *J Behav Med* 2007; **30**: 303–9.

628 Cotman CW, Berchtold NC, Christie L-A. Exercise builds brain health: key roles of growth factor cascades and inflammation. *Trends in Neurosciences* 2007; **30**: 464–72.

629 Erickson KG, Crosnoe R, Dornbusch SM. A Social Process Model of Adolescent Deviance: Combining Social Control and Differential Association Perspectives. *Journal of Youth and Adolescence* 2000; **29**: 395–425.

630 Fancourt D, Aufegger L, Williamson A. Low-stress and high-stress singing have contrasting effects on glucocorticoid response. *Front Psychol* 2015; **6**: 1242.

631 Fancourt D, Perkins R. The effects of mother–infant singing on emotional closeness, affect, anxiety, and stress hormones. *Music & Science* 2018; **1**: 2059204317745746.

632 Fancourt D, Williamon A. Attending a concert reduces glucocorticoids, progesterone and the cortisol/DHEA ratio. *Public Health* 2016; **132**: 101–4.

633 Kaimal G, Ray K, Muniz J. Reduction of Cortisol Levels and Participants’ Responses Following Art Making. *Art Therapy* 2016; **33**: 74–80.

634 Edwards DA, Wetzel K, Wyner DR. Intercollegiate soccer: Saliva cortisol and testosterone are elevated during competition, and testosterone is related to status and social connectedness with teammates. *Physiology & Behavior* 2006; **87**: 135–43.

635 Leelarungrayub D, Saidee K, Pothongsunun P, Pratanaphon S, YanKai A, Bloomer RJ. Six weeks of aerobic dance exercise improves blood oxidative stress status and increases interleukin-2 in previously sedentary women. *Journal of Bodywork and Movement Therapies* 2011; **15**: 355–62.

636 Santos RVT, Viana VAR, Boscolo RA, *et al.* Moderate exercise training modulates cytokine profile and sleep in elderly people. *Cytokine* 2012; **60**: 731–5.

637 Cole SW, Hawkley LC, Arevalo JM, Sung CY, Rose RM, Cacioppo JT. Social regulation of gene expression in human leukocytes. *Genome Biol* 2007; **8**: R189.

638 Dunphy K, Baker FA, Dumaresq E, *et al.* Creative Arts Interventions to Address Depression in Older Adults: A Systematic Review of Outcomes, Processes, and Mechanisms. *Front Psychol* 2019; **9**. DOI:10.3389/fpsyg.2018.02655.

639 Fancourt D, Ockelford A, Belai A. The psychoneuroimmunological effects of music: A systematic review and a new model. *Brain, Behavior, and Immunity* 2014; **36**: 15–26.

640 Aschbacher K, O’Donovan A, Wolkowitz OM, Dhabhar FS, Su Y, Epel E. Good stress, bad stress and oxidative stress: Insights from anticipatory cortisol reactivity. *Psychoneuroendocrinology* 2013; **38**: 1698–708.

641 Hannestad J, DellaGioia N, Bloch M. The effect of antidepressant medication treatment on serum levels of inflammatory cytokines: a meta-analysis. *Neuropsychopharmacology* 2011; **36**: 2452–9.

642 Hawkley LC, Cole SW, Capitanio JP, Norman GJ, Cacioppo JT. Effects of Social Isolation on Glucocorticoid Regulation in Social Mammals. *Horm Behav* 2012; **62**: 314–23.

643 Inagaki TK, Muscatell KA, Irwin MR, *et al.* The role of the ventral striatum in inflammatory-induced approach toward support figures. *Brain Behav Immun* 2015; **44**: 247–52.

644 Moieni M, Eisenberger NI. Effects of inflammation on social processes and implications for health. *Annals of the New York Academy of Sciences* 2018; **1428**: 5–13.

645 Snyder CR, Lopez SJ. Oxford Handbook of Positive Psychology. Oxford University Press, 2009.

646 Sonntag WE, Ramsey M, Carter CS. Growth hormone and insulin-like growth factor-1 (IGF-1) and their influence on cognitive aging. *Ageing Research Reviews* 2005; **4**: 195–212.

647 Martin W H, Montgomery J, Snell P G, *et al.* Cardiovascular adaptations to intense swim training in sedentary middle-aged men and women. *Circulation* 1987; **75**: 323–30.

648 Montoya P, Campos JJ, Schandry R. See red? Turn pale? Unveiling Emotions through Cardiovascular and Hemodynamic Changes. *The Spanish Journal of Psychology* 2005; **8**: 79–85.

649 Spalding TW, Jeffers LS, Porges SW, Hatfield BD. Vagal and cardiac reactivity to psychological stressors in trained and untrained men. *Medicine & Science in Sports & Exercise* 2000; **32**: 581–91.

650 Spalding TW, Lyon LA, Steel DH, Hatfield BD. Aerobic exercise training and cardiovascular reactivity to psychological stress in sedentary young normotensive men and women. *Psychophysiology* 2004; **41**: 552–62.

651 Vlachopoulos C, Xaplanteris P, Alexopoulos N, *et al.* Divergent Effects of Laughter and Mental Stress on Arterial Stiffness and Central Hemodynamics. *Psychosomatic Medicine* 2009; **71**: 446–453.

652 Haapanen N, Miilunpalo S, Vuori I, Oja P, Pasanen M. Characteristics of Leisure Time Physical Activity Associated with Decreased Risk of Premature All-Cause and Cardiovascular Disease Mortality in Middle-aged Men. *Am J Epidemiol* 1996; **143**: 870–80.

653 Steptoe A, Wardle J. Positive affect and biological function in everyday life. *Neurobiology of Aging* 2005; **26**: 108–12.

654 Wang Z, Marseglia A, Shang Y, Dintica C, Patrone C, Xu W. Leisure activity and social integration mitigate the risk of dementia related to cardiometabolic diseases: A population-based longitudinal study. *Alzheimer’s & Dementia* 2019; published online Nov 9. DOI:10.1016/j.jalz.2019.09.003.

655 Cacioppo JT, Hawkley LC. Social Isolation and Health, with an Emphasis on Underlying Mechanisms. *Perspectives in Biology and Medicine* 2003; **46**: S39–52.

656 Ellis RJ, Thayer JF. Music and Autonomic Nervous System (Dys)function. *Music Percept* 2010; **27**: 317–26.

657 Alenghat T. Epigenomics and the microbiota. *Toxicologic pathology* 2015; **43**: 101–106.

658 Carney R, Cotter J, Bradshaw T, Firth J, Yung AR. Cardiometabolic risk factors in young people at ultra-high risk for psychosis: A systematic review and meta-analysis. *Schizophrenia Research* 2016; **170**: 290–300.

659 Curtis BM, O’Keefe JH. Autonomic Tone as a Cardiovascular Risk Factor: The Dangers of Chronic Fight or Flight. *Mayo Clinic Proceedings* 2002; **77**: 45–54.

660 Health I of M (US) C on, Practice, Policy. Health and behavior: The interplay of biological, behavioral, and societal influences. National Academies Press, 2001.

661 Hjemdahl P, Rosengren A, Steptoe A. Stress and cardiovascular disease. Springer Science & Business Media, 2011.

662 Janssen I, Katzmarzyk PT, Ross R. Body Mass Index, Waist Circumference, and Health Risk: Evidence in Support of Current National Institutes of Health Guidelines. *Arch Intern Med* 2002; **162**: 2074–9.

663 Jones RM. The Influence of the Gut Microbiota on Host Physiology: In Pursuit of Mechanisms. *Yale J Biol Med* 2016; **89**: 285–97.

664 Logi Kristjánsson Á, Dóra Sigfúsdóttir I, Allegrante JP. Health Behavior and Academic Achievement Among Adolescents: The Relative Contribution of Dietary Habits, Physical Activity, Body Mass Index, and Self-Esteem. *Health Educ Behav* 2010; **37**: 51–64.

665 Bonnefoy M, Boutitie F, Mercier C, *et al.* Efficacy of a home-based intervention programme on the physical activity level and functional ability of older people using domestic services: A randomised study. *J Nutr Health Aging* 2012; **16**: 370–7.

666 Granbom M, Kristensson J, Sandberg M. Effects on leisure activities and social participation of a case management intervention for frail older people living at home: a randomised controlled trial. *Health & Social Care in the Community* 2017; **25**: 1416–29.

667 Hurkmans J, Bruijn M de, Boonstra AM, *et al.* Music in the treatment of neurological language and speech disorders: A systematic review. *Aphasiology* 2012; **26**: 1–19.

668 Lewis A, Cave P, Stern M, *et al.* Singing for Lung Health-a systematic review of the literature and consensus statement. *NPJ Prim Care Respir Med* 2016; **26**: 16080.

669 Fallahpour M, Borell L, Luborsky M, Nygård L. Leisure-activity participation to prevent later-life cognitive decline: a systematic review. *Scandinavian Journal of Occupational Therapy* 2016; **23**: 162–97.

670 Chen Y-C, Lin K, Chen C-J, Wang C-H. Effects of leisure-time physical activity interventions on frailty-related characteristics of frail older adults in long-term care: a systematic review. *Contemporary Nurse* 2020; **0**: 1–15.

671 Fong Yan A, Cobley S, Chan C, *et al.* The Effectiveness of Dance Interventions on Physical Health Outcomes Compared to Other Forms of Physical Activity: A Systematic Review and Meta-Analysis. *Sports Med* 2018; **48**: 933–51.

672 Hwang PW-N, Braun KL. The Effectiveness of Dance Interventions to Improve Older Adults’ Health: A Systematic Literature Review. *Altern Ther Health Med* 2015; **21**: 64–70.

673 Barron JS, Tan EJ, Yu Q, Song M, McGill S, Fried LP. Potential for Intensive Volunteering to Promote the Health of Older Adults in Fair Health. *J Urban Health* 2009; **86**: 641–53.

674 Chen J-C, Chang Q-X, Liang C-C, *et al.* Potential benefits of environmental volunteering programs of the health of older adults: a pilot study. *Archives of Gerontology and Geriatrics* 2020; **90**: 104113.

675 Lewis A, Cave P, Stern M, *et al.* Singing for Lung Health—a systematic review of the literature and consensus statement. *NPJ Prim Care Respir Med* 2016; **26**: 16080.

676 Penedo FJ, Dahn JR. Exercise and well-being: a review of mental and physical health benefits associated with physical activity. *Current Opinion in Psychiatry* 2005; **18**: 189–193.

677 Calderón‐Larrañaga A, Vetrano DL, Ferrucci L, *et al.* Multimorbidity and functional impairment–bidirectional interplay, synergistic effects and common pathways. *Journal of Internal Medicine* 2019; **285**: 255–71.

678 Fabrício D de M, Chagas MHN, Diniz BS. Frailty and cognitive decline. *Translational Research* 2020; published online Jan 23. DOI:10.1016/j.trsl.2020.01.002.

679 Fowers BJ, Richardson FC, Slife BD. Frailty, suffering, and vice: Flourishing in the face of human limitations. American Psychological Association, 2017.

680 Keevil VL, Luben R, Hayat S, Sayer AA, Wareham NJ, Khaw K-T. Physical capability predicts mortality in late mid-life as well as in old age: Findings from a large British cohort study. *Archives of Gerontology and Geriatrics* 2018; **74**: 77–82.

681 Toyokawa S, Uddin M, Koenen KC, Galea S. How does the social environment ‘get into the mind’? Epigenetics at the intersection of social and psychiatric epidemiology. *Soc Sci Med* 2012; **74**: 67–74.

682 Cherkas LF, Hunkin JL, Kato BS, *et al.* The association between physical activity in leisure time and leukocyte telomere length. *Arch Intern Med* 2008; **168**: 154–8.

683 Shadyab AH, LaMonte MJ, Kooperberg C, *et al.* Leisure-time physical activity and leukocyte telomere length among older women. *Exp Gerontol* 2017; **95**: 141–7.

684 Sillanpää E, Ollikainen M, Kaprio J, *et al.* Leisure-time physical activity and DNA methylation age—a twin study. *Clin Epigenet* 2019; **11**: 12.

685 Hoge EA, Chen MM, Orr E, *et al.* Loving-Kindness Meditation practice associated with longer telomeres in women. *Brain, Behavior, and Immunity* 2013; **32**: 159–63.

686 Woo J, Tang N, Suen E, Leung J, Wong M. Green space, psychological restoration, and telomere length. *The Lancet* 2009; **373**: 299–300.

687 Forrester SN, Leoutsakos J-M, Gallo JJ, Thorpe RJ, Seeman TE. Association between allostatic load and health behaviours: a latent class approach. *J Epidemiol Community Health* 2019; **73**: 340–5.

688 Upchurch DM, Rainisch BW, Chyu L. Greater Leisure Time Physical Activity Is Associated with Lower Allostatic Load in White, Black, and Mexican American Midlife Women: Findings from the National Health and Nutrition Examination Survey, 1999 through 2004. *Women’s Health Issues* 2015; **25**: 680–7.

689 Wiley JF, Bei B, Bower JE, Stanton AL. Relationship of Psychosocial Resources with Allostatic Load: A Systematic Review. *Psychosom Med* 2017; **79**: 283–92.

690 Bergman Y, Cedar H. DNA methylation dynamics in health and disease. *Nature Structural & Molecular Biology* 2013; **20**: 274–81.

691 Bojesen SE. Telomeres and human health. *Journal of Internal Medicine* 2013; **274**: 399–413.

692 Cesari M, Prince M, Thiyagarajan JA, *et al.* Frailty: An Emerging Public Health Priority. *Journal of the American Medical Directors Association* 2016; **17**: 188–92.

693 Sasaki A, Vega WC de, McGowan PO. Biological embedding in mental health: An epigenomic perspective. *Biochem Cell Biol* 2013; **91**: 14–21.

694 MacKinnon K, van Ham C, Reilly K, Hopkins J. Nature-Based Solutions and Protected Areas to Improve Urban Biodiversity and Health. In: Marselle MR, Stadler J, Korn H, Irvine KN, Bonn A, eds. Biodiversity and Health in the Face of Climate Change. Cham: Springer International Publishing, 2019: 363–80.

695 Marselle MR. Theoretical Foundations of Biodiversity and Mental Well-being Relationships. In: Marselle MR, Stadler J, Korn H, Irvine KN, Bonn A, eds. Biodiversity and Health in the Face of Climate Change. Cham: Springer International Publishing, 2019: 133–58.

696 Burls A. People and green spaces: promoting public health and mental well‐being through ecotherapy. *Journal of Public Mental Health* 2007; **6**: 24–39.

697 Butler CD, Friel S. Time to Regenerate: Ecosystems and Health Promotion. *PLOS Medicine* 2006; **3**: e394.

698 Marselle M, Stadler J, Korn H, Irvine K, Bonn A, editors. Biodiversity and Health in the Face of Climate Change. Springer International Publishing, 2019 DOI:10.1007/978-3-030-02318-8.

699 Lucas RM, Ponsonby A-L, Dear K, *et al.* Sun exposure and vitamin D are independent risk factors for CNS demyelination. *Neurology* 2011; **76**: 540–8.

700 Rui MD, Toffanello ED, Veronese N, *et al.* Vitamin D Deficiency and Leisure Time Activities in the Elderly: Are All Pastimes the Same? *PLOS ONE* 2014; **9**: e94805.

701 Scragg R, Camargo CA. Frequency of Leisure-Time Physical Activity and Serum 25-Hydroxyvitamin D Levels in the US Population: Results from the Third National Health and Nutrition Examination Survey. *Am J Epidemiol* 2008; **168**: 577–86.

702 Bowler DE, Buyung-Ali LM, Knight TM, Pullin AS. A systematic review of evidence for the added benefits to health of exposure to natural environments. *BMC Public Health* 2010; **10**: 456.

703 Kuo FE, Sullivan WC, Coley RL, Brunson L. Fertile Ground for Community: Inner-City Neighborhood Common Spaces. *Am J Community Psychol* 1998; **26**: 823–51.

704 Lachowycz K, Jones AP. Greenspace and obesity: a systematic review of the evidence. *Obes Rev* 2011; **12**: e183-189.

705 Marselle MR, Martens D, Dallimer M, Irvine KN. Review of the Mental Health and Well-being Benefits of Biodiversity. In: Marselle MR, Stadler J, Korn H, Irvine KN, Bonn A, eds. Biodiversity and Health in the Face of Climate Change. Cham: Springer International Publishing, 2019: 175–211.

706 Pearson DG, Craig T. The great outdoors? Exploring the mental health benefits of natural environments. *Front Psychol* 2014; **5**. DOI:10.3389/fpsyg.2014.01178.

707 Prescott SL, Millstein RA, Katzman MA, Logan AC. Biodiversity, the Human Microbiome and Mental Health: Moving toward a New Clinical Ecology for the 21st Century? *International Journal of Biodiversity* 2016; **2016**: 1–18.

708 Yin J, Zhu S, MacNaughton P, Allen JG, Spengler JD. Physiological and cognitive performance of exposure to biophilic indoor environment. *Building and Environment* 2018; **132**: 255–62.

709 Clatworthy J, Hinds J, M. Camic P. Gardening as a mental health intervention: a review. *Mental Health Review Journal* 2013; **18**: 214–25.

710 Park S-A, Lee A-Y, Son K-C, Lee W-L, Kim D-S. Gardening Intervention for Physical and Psychological Health Benefits in Elderly Women at Community Centers. *HortTechnology* 2016; **26**: 474–83.

711 Park S-A, Lee A-Y, Park H-G, Son K-C, Kim D-S, Lee W-L. Gardening Intervention as a Low- to Moderate-Intensity Physical Activity for Improving Blood Lipid Profiles, Blood Pressure, Inflammation, and Oxidative Stress in Women over the Age of 70: A Pilot Study. *HortScience* 2017; **52**: 200–5.

712 Bail JR, Frugé AD, Cases MG, *et al.* A home-based mentored vegetable gardening intervention demonstrates feasibility and improvements in physical activity and performance among breast cancer survivors. *Cancer* 2018; **124**: 3427–35.

713 Christian MS, Evans CE, Nykjaer C, Hancock N, Cade JE. Evaluation of the impact of a school gardening intervention on children’s fruit and vegetable intake: a randomised controlled trial. *Int J Behav Nutr Phys Act* 2014; **11**: 99.

714 Hunter RF, Christian H, Veitch J, Astell-Burt T, Hipp JA, Schipperijn J. The impact of interventions to promote physical activity in urban green space: A systematic review and recommendations for future research. *Social Science & Medicine* 2015; **124**: 246–56.

715 Rovio S, Kåreholt I, Helkala E-L, *et al.* Leisure-time physical activity at midlife and the risk of dementia and Alzheimer’s disease. *The Lancet Neurology* 2005; **4**: 705–11.

716 Roe JJ, Thompson CW, Aspinall PA, *et al.* Green Space and Stress: Evidence from Cortisol Measures in Deprived Urban Communities. *Int J Environ Res Public Health* 2013; **10**: 4086–103.

717 Hu Z, Liebens J, Rao KR. Linking stroke mortality with air pollution, income, and greenness in northwest Florida: an ecological geographical study. *International Journal of Health Geographics* 2008; **7**: 20.

718 Maas J, Verheij RA, Vries S de, Spreeuwenberg P, Schellevis FG, Groenewegen PP. Morbidity is related to a green living environment. *Journal of Epidemiology & Community Health* 2009; **63**: 967–73.

719 Arem H, Moore SC, Patel A, *et al.* Leisure time physical activity and mortality: a detailed pooled analysis of the dose-response relationship. *JAMA Intern Med* 2015; **175**: 959–67.

720 Takano T, Nakamura K, Watanabe M. Urban residential environments and senior citizens’ longevity in megacity areas: the importance of walkable green spaces. *Journal of Epidemiology & Community Health* 2002; **56**: 913–8.

721 Huron D. Is Music an Evolutionary Adaptation? *Annals of the New York Academy of Sciences* 2001; **930**: 43–61.

722 Masataka N. Music, evolution and language. *Developmental Science* 2007; **10**: 35–9.

723 Schulkin J, Raglan GB. The evolution of music and human social capability. *Front Neurosci* 2014; **8**. DOI:10.3389/fnins.2014.00292.

724 Ambeskovic M, Roseboom TJ, Metz GAS. Transgenerational effects of early environmental insults on aging and disease incidence. *Neuroscience & Biobehavioral Reviews* 2017; published online Aug 12. DOI:10.1016/j.neubiorev.2017.08.002.

725 Babenko O, Kovalchuk I, Metz GAS. Stress-induced perinatal and transgenerational epigenetic programming of brain development and mental health. *Neuroscience & Biobehavioral Reviews* 2015; **48**: 70–91.

726 Cowan CP, Cowan PA. Two central roles for couple relationships: breaking negative intergenerational patterns and enhancing children’s adaptation. *Sexual and Relationship Therapy* 2005; **20**: 275–88.

727 Gracey M, King M. Indigenous health part 1: determinants and disease patterns. *The Lancet* 2009; **374**: 65–75.

728 Rodgers A, Ezzati M, Vander Hoorn S, *et al.* Distribution of major health risks: findings from the Global Burden of Disease study. *PLoS medicine* 2004; **1**.

729 Mahoney JL, Stattin H. Leisure activities and adolescent antisocial behavior: The role of structure and social context. *Journal of Adolescence* 2000; **23**: 113–27.

730 Messner SF, Blau JR. Routine Leisure Activities and Rates of Crime: A Macro-Level Analysis. *Soc Forces* 1987; **65**: 1035–52.

731 Sauerwein M, Theis D, Fischer N. How Youths’ Profiles of Extracurricular and Leisure Activity Affect Their Social Development and Academic Achievement. *IJREE – International Journal for Research on Extended Education* 2016; **4**. https://www.budrich-journals.de/index.php/IJREE/article/view/24778 (accessed July 24, 2020).

732 Kahana E, Bhatta T, Lovegreen LD, Kahana B, Midlarsky E. Altruism, Helping, and Volunteering: Pathways to Well-Being in Late Life. *J Aging Health* 2013; **25**: 159–87.

733 Leroux K, Bernadska A. Impact of the Arts on Individual Contributions to US Civil Society. *Journal of Civil Society* 2014; **10**: 144–64.

734 Van de Vyver J, Abrams D. The Arts as a Catalyst for Human Prosociality and Cooperation. *Social Psychological and Personality Science* 2018; **9**: 664–74.

735 Coghlan A. Prosocial behaviour in volunteer tourism. *Annals of Tourism Research* 2015; **55**: 46–60.

736 Coleman D, Iso-Ahola SE. Leisure and Health: The Role of Social Support and Self-Determination. *Journal of Leisure Research* 1993; **25**: 111–28.

737 Hemingway JL. Leisure, Social Capital, and Democratic Citizenship. *Journal of Leisure Research* 1999; **31**: 150–65.

738 Hogg J. Creative, Personal and Social Engagement in the Later Years: Realisation Through Leisure. *The Irish Journal of Psychology* 1993; **14**: 204–18.

739 Greitemeyer T. Effects of Songs With Prosocial Lyrics on Prosocial Behavior: Further Evidence and a Mediating Mechanism: *Personality and Social Psychology Bulletin* 2009; published online July 31. DOI:10.1177/0146167209341648.

740 Goldstein H, Wickstrom S, Hoyson M, Jamieson B, Odom SL. EFFECTS OF SOCIODRAMATIC SCRIPT TRAINING ON SOCIAL AND COMMUNICATIVE INTERACTION. *Education and Treatment of Children* 1988; **11**: 97–117.

741 Haring TG, Breen CG. A Peer-Mediated Social Network Intervention to Enhance the Social Integration of Persons with Moderate and Severe Disabilities. *Journal of Applied Behavior Analysis* 1992; **25**: 319–33.

742 Rogers SJ. Interventions That Facilitate Socialization in Children with Autism. *J Autism Dev Disord* 2000; **30**: 399–409.

743 Ar H, Mb O, Lm W. Social engagement and its relationship to health. *Clin Geriatr Med* 2002; **18**: 593–609, ix.

744 Cruwys T, Bevelander KE, Hermans RCJ. Social modeling of eating: A review of when and why social influence affects food intake and choice. *Appetite* 2015; **86**: 3–18.

745 Eisenberg D, Golberstein E, Whitlock JL, Downs MF. Social Contagion of Mental Health: Evidence from College Roommates. *Health Economics* 2013; **22**: 965–86.

746 Hsu C-P, Chang C-W, Huang H-C, Chiang C-Y. The relationships among social capital, organisational commitment and customer-oriented prosocial behaviour of hospital nurses. *Journal of Clinical Nursing* 2011; **20**: 1383–92.

747 Li Y-P, Chen Y-M, Chen C-H. Volunteer transitions and physical and psychological health among older adults in Taiwan. *J Gerontol B Psychol Sci Soc Sci* 2013; **68**: 997–1008.

748 Post SG. Altruism and health: Perspectives from empirical research. Oxford University Press, 2007.

749 Wood W. Attitude Change: Persuasion and Social Influence. *Annual Review of Psychology* 2000; **51**: 539–70.

750 Camic PM, Tischler V, Pearman CH. Viewing and making art together: a multi-session art-gallery-based intervention for people with dementia and their carers. *Aging & Mental Health* 2014; **18**: 161–8.

751 Dadswell A, Bungay H, Wilson C, Munn-Giddings C. The impact of participatory arts in promoting social relationships for older people within care homes. *Perspect Public Health* 2020; : 1757913920921204.

752 Machtinger EL, Lavin SM, Hilliard S, *et al.* An Expressive Therapy Group Disclosure Intervention for Women Living With HIV Improves Social Support, Self-efficacy, and the Safety and Quality of Relationships: A Qualitative Analysis. *Journal of the Association of Nurses in AIDS Care* 2015; **26**: 187–98.

753 Chang P-J, Wray L, Lin Y. Social relationships, leisure activity, and health in older adults. *Health Psychology* 2014; **33**: 516–23.

754 Fitzpatrick TR. The Quality of Dyadic Relationships, Leisure Activities and Health Among Older Women. *Healthcare for Women International* 2009; **30**: 1073–92.

755 Smart E, Edwards B, Kingsnorth S, *et al.* Creating an inclusive leisure space: strategies used to engage children with and without disabilities in the arts-mediated program Spiral Garden. *Disability and Rehabilitation* 2018; **40**: 199–207.

756 Berkman LF, Krishna A. Social Network Epidemiology. In: Kawachi I, Glymour M, Berkman LF, eds. Social Epidemiology, Second Edition. Oxford, New York: Oxford University Press, 2014.

757 Edwards HB, Savović J, Whiting P, *et al.* Quality of relationships as predictors of outcomes in people with dementia: a systematic review protocol. *BMJ Open* 2016; **6**: e010835.

758 Hawkley LC, Cacioppo JT. Loneliness Matters: A Theoretical and Empirical Review of Consequences and Mechanisms. *Ann Behav Med* 2010; **40**: 218–27.

759 Agnihotri S, Gray J, Colantonio A, *et al.* Two case study evaluations of an arts-based social skills intervention for adolescents with childhood brain disorder. *Developmental Neurorehabilitation* 2012; **15**: 284–97.

760 Chick G, Barnett LA. Children’s play and adult leisure. *The future of play theory: A multidisciplinary inquiry into the contributions of Brian Sutton-Smith* 1995; : 45–69.

761 Christenson OD, Zabriskie RB, Eggett DL, Freeman PA. Family Acculturation, Family Leisure Involvement, and Family Functioning among Mexican-Americans. *Journal of Leisure Research* 2006; **38**: 475–95.

762 Epp KM. Outcome-Based Evaluation of a Social Skills Program Using Art Therapy and Group Therapy for Children on the Autism Spectrum. *Child Sch* 2008; **30**: 27–36.

763 Falk JH, Storksdieck M. Science learning in a leisure setting. *Journal of Research in Science Teaching* 2010; **47**: 194–212.

764 Joubert L. Creative communities: The arts, social responsibility and sustainable planning and development. *WIT Transactions on Ecology and the Environment* 2004; **72**.

765 Madrid AL. Dancing with desire: cultural embodiment in Tijuana’s Nor-tec music and dance. *Popular Music* 2006; **25**: 383–99.

766 Packer J. Learning for Fun: The Unique Contribution of Educational Leisure Experiences. *Curator: The Museum Journal* 2006; **49**: 329–44.

767 Roberson Jr DN. Leisure and learning: An investigation of older adults and self‐directed learning. *Leisure/Loisir* 2010; published online Nov 21. https://www.tandfonline.com/doi/abs/10.1080/14927713.2005.9651330 (accessed July 27, 2020).

768 Roggenbuck JW, Loomis RJ, Dagostino J. The Learning Benefits of Leisure. *Journal of Leisure Research* 1990; **22**: 112–24.

769 Sargant N. Learning and leisure. *Boundaries of adult learning* 1996; **3**: 196–210.

770 Ericsson I, Karlsson MK. Motor skills and school performance in children with daily physical education in school – a 9-year intervention study. *Scandinavian Journal of Medicine & Science in Sports* 2014; **24**: 273–8.

771 Argyle M. Social skills and health (psychology revivals). Routledge, 2013.

772 Christensen PH. ‘It Hurts’: Children’s Cultural Learning About Everyday Illness. *Etnofoor* 1999; **12**: 39–52.

773 Gurung RAR. Health Psychology: A Cultural Approach. Cengage Learning, 2013.

774 Hwang W-C, Ting JY. Disaggregating the effects of acculturation and acculturative stress on the mental health of Asian Americans. *Cultural Diversity and Ethnic Minority Psychology* 2008; **14**: 147–54.

775 Johnson JL, Bottorff JL, Browne AJ, Grewal S, Hilton BA, Clarke H. Othering and Being Othered in the Context of Healthcare Services. *Health Communication* 2004; **16**: 255–71.

776 Ingen EV, Eijck KV. Leisure and Social Capital: An Analysis of Types of Company and Activities. *Leisure Sciences* 2009; **31**: 192–206.

777 Iwasaki Y, MacTavish J, MacKay K. Building on strengths and resilience: leisure as a stress survival strategy. *British Journal of Guidance & Counselling* 2005; **33**: 81–100.

778 Kleiber DA, Hutchinson SL, Williams R. Leisure as a Resource in Transcending Negative Life Events: Self-Protection, Self-Restoration, and Personal Transformation. *Leisure Sciences* 2002; **24**: 219–35.

779 Ohmer ML, Meadowcroft P, Freed K, Lewis E. Community Gardening and Community Development: Individual, Social and Community Benefits of a Community Conservation Program. *Journal of Community Practice* 2009; **17**: 377–99.

780 Shinew KJ, Glover TD, Parry DC. Leisure Spaces as Potential Sites for Interracial Interaction: Community Gardens in Urban Areas. *Journal of Leisure Research* 2004; **36**: 336–55.

781 Dimanche F, Samdahl D. Leisure as symbolic consumption: A conceptualization and prospectus for future research. *Leisure Sciences* 1994; **16**: 119–29.

782 Glover TD, Hemingway JL. Locating Leisure in the Social Capital Literature. *Journal of Leisure Research* 2005; **37**: 387–401.

783 Haslam C, Jetten J, Cruwys T, Dingle G, Haslam SA. The New Psychology of Health: Unlocking the Social Cure, 1 edition. New York: Routledge, 2018.

784 Haslam SA, Jetten J, Postmes T, Haslam C. Social Identity, Health and Well-Being: An Emerging Agenda for Applied Psychology. *Applied Psychology* 2009; **58**: 1–23.

785 Shannon CS, Bourque D. Overlooked and Underutilized: The Critical Role of Leisure Interventions in Facilitating Social Support Throughout Breast Cancer Treatment and Recovery. *Social Work in Healthcare* 2006; **42**: 73–92.

786 Cohen S. Social support and health. Academic Press, 1985.

787 Cutler DM, Lleras-Muney A, Vogl T. Socioeconomic Status and Health: Dimensions and Mechanisms. National Bureau of Economic Research, 2008 DOI:10.3386/w14333.

788 Derose KP, Varda DM. Social capital and healthcare access: a systematic review. *Medical care research and review: MCRR* 2009; **66**: 272.

789 Hofmeyer A, Marck PB. Building social capital in healthcare organizations: thinking ecologically for safer care. *Nursing Outlook* 2008; **56**: 145–e1.

790 Kawachi I, Berkman L. Social cohesion, social capital, and health. *Social epidemiology* 2000; **174**.

791 Kelly ME, Duff H, Kelly S, *et al.* The impact of social activities, social networks, social support and social relationships on the cognitive functioning of healthy older adults: a systematic review. *Syst Rev* 2017; **6**: 259.

792 Arai S, Pedlar A. Moving beyond individualism in leisure theory: a critical analysis of concepts of community and social engagement. *Leisure Studies* 2003; **22**: 185–202.

793 Braubach M, Egorov A, Mudu P, Wolf T, Thompson CW, Martuzzi M. Effects of urban green space on environmental health, equity and resilience. In: Nature-based solutions to climate change adaptation in urban areas. Springer, Cham, 2017: 187–205.

794 Breunig M, O’Connell T, Sharon T. Psychological Sense of Community and Group Cohesion on Wilderness Trips. *Journal of Experiential Education* 2008; **30, No. 3**: 258–61.

795 Christensen U, Schmidt L, Budtz-Jørgensen E, Avlund K. Group Cohesion and Social Support in Exercise Classes: Results From a Danish Intervention Study. *Health Educ Behav* 2006; **33**: 677–89.

796 Glass JS, Benshoff JM. Facilitating Group Cohesion among Adolescents through Challenge Course Experiences. *Journal of Experiential Education* 2002; **25**: 268–77.

797 Jennings V, Bamkole O. The Relationship between Social Cohesion and Urban Green Space: An Avenue for Health Promotion. *International Journal of Environmental Research and Public Health* 2019; **16**: 452.

798 Koch SC. Arts and health: Active factors and a theory framework of embodied aesthetics. *The Arts in Psychotherapy* 2017; **54**: 85–91.

799 Lee D. How the Arts Generate Social Capital to Foster Intergroup Social Cohesion. *The Journal of Arts Management, Law, and Society* 2013; **43**: 4–17.

800 Orthner DK, Mancini JA. Leisure Impacts on Family Interaction and Cohesion. *Journal of Leisure Research* 1990; **22**: 125–37.

801 Theorell T. Music in Social Cohesion. In: Psychological Health Effects of Musical Experiences. Springer Netherlands, 2014: 17–27.

802 Eckersley R. Population health: a forgotten dimension of social resilience. *Resilience and transformation: preparing Australia for uncertain futures* 2010; : 115–120.

803 Poortinga W. Community resilience and health: The role of bonding, bridging, and linking aspects of social capital. *Health & Place* 2012; **18**: 286–95.

804 Wallace R, Wallace D. Socioeconomic determinants of health: Community marginalisation and the diffusion of disease and disorder in the United States. *BMJ* 1997; **314**: 1341.

805 Arai SM, Pedlar AM. Building Communities Through Leisure: Citizen Participation in a Healthy Communities Initiative. *Journal of Leisure Research* 1997; **29**: 167–82.

806 Drinkwater C, Wildman J, Moffatt S. Social prescribing. *BMJ* 2019; : l1285.

807 Stratton LS, Datta Gupta N. Institutions, Social Norms, and Bargaining Power: An Analysis of Individual Leisure Time in Couple Households. Rochester, NY: Social Science Research Network, 2008 DOI:10.1111/j.0042-7092.2007.00700.x.

808 Wearing B. Beyond the Ideology of Motherhood: Leisure as Resistance. *The Australian and New Zealand Journal of Sociology* 1990; **26**: 36–58.

809 Wearing B, Wearing S. ‘All in a day’s leisure’: gender and the concept of leisure. *Leisure Studies* 1988; **7**: 111–23.

810 Jones P, Comfort D, Eastwood I, Hillier D. Creative industries: economic contributions, management challenges and support initiatives. *Management Research News* 2004; **27**: 134–45.

811 Kondo MC, South EC, Branas CC. Nature-Based Strategies for Improving Urban Health and Safety. *J Urban Health* 2015; **92**: 800–14.

812 Roberts K. Leisure inequalities, class divisions and social exclusion in present-day Britain. 2004. DOI:10.1080/0954896042000267152.

813 Scherger S, Savage M. Cultural Transmission, Educational Attainment and Social Mobility: *The Sociological Review* 2010; published online Aug 1. https://journals.sagepub.com/doi/10.1111/j.1467-954X.2010.01927.x (accessed Aug 4, 2020).

814 Farmer P. Pathologies of Power: Health, Human Rights, and the New War on the Poor. University of California Press, 2004.

815 Laverack G. Health Promotion Practice: Power and Empowerment. SAGE, 2004.

816 Link BG, Phelan J. Stigma power. *Social Science & Medicine* 2014; **103**: 24–32.

817 Parker J, DeLay D. The future of the healthcare supply chain: suppliers wield considerable power, but healthcare organizations can benefit from virtual centralization of the supply chain. *Healthcare Financial Management* 2008; **62**: 66–70.

818 Dawson C, Baker PL, Dowell D. Getting into the ‘Giving Habit’: The Dynamics of Volunteering in the UK. *Voluntas* 2019; **30**: 1006–21.

819 Hu T, Stafford TF, Kettinger WJ, Zhang X “Paul”, Dai H. Formation and Effect of Social Media Usage Habit. *Journal of Computer Information Systems* 2018; **58**: 334–43.

820 Kovacsik R, Griffiths MD, Pontes HM, *et al.* The Role of Passion in Exercise Addiction, Exercise Volume, and Exercise Intensity in Long-term Exercisers. *Int J Ment Health Addiction* 2019; **17**: 1389–400.

821 Kraaykamp G. Literary socialization and reading preferences. Effects of parents, the library, and the school. *Poetics* 2003; **31**: 235–57.

822 Rhodes RE, Rebar AL. Physical Activity Habit: Complexities and Controversies. In: Verplanken B, ed. The Psychology of Habit: Theory, Mechanisms, Change, and Contexts. Cham: Springer International Publishing, 2018: 91–109.

823 Schmutz V, Stearns E, Glennie EJ. Cultural capital formation in adolescence: High schools and the gender gap in arts activity participation. *Poetics* 2016; **57**: 27–39.

824 Tappe K, Tarves E, Oltarzewski J, Frum D. Habit Formation Among Regular Exercisers at Fitness Centers: An Exploratory Study. *Journal of Physical Activity and Health* 2013; **10**: 607–13.

825 Leme ACB, Lubans DR, Guerra PH, Dewar D, Toassa EC, Philippi ST. Preventing obesity among Brazilian adolescent girls: Six-month outcomes of the Healthy Habits, Healthy Girls–Brazil school-based randomized controlled trial. *Preventive Medicine* 2016; **86**: 77–83.

826 Aşcı Ö, Rathfisch G. Effect of lifestyle interventions of pregnant women on their dietary habits, lifestyle behaviors, and weight gain: a randomized controlled trial. *J Health Popul Nutr* 2016; **35**: 7.

827 Kaufman-Shriqui V, Fraser D, Friger M, *et al.* Effect of a School-Based Intervention on Nutritional Knowledge and Habits of Low-Socioeconomic School Children in Israel: A Cluster-Randomized Controlled Trial. *Nutrients* 2016; **8**: 234.

828 Gardner B, Rebar AL, Lally P. A matter of habit: Recognizing the multiple roles of habit in health behaviour. *British Journal of Health Psychology* 2019; **24**: 241–9.

829 Karl FM, Holle R, Schwettmann L, Peters A, Laxy M. Status quo bias and health behavior: findings from a cross-sectional study. *Eur J Public Health* 2019; **29**: 992–7.

830 Thomas GO, Poortinga W, Sautkina E. Habit Discontinuity, Self-Activation, and the Diminishing Influence of Context Change: Evidence from the UK Understanding Society Survey. *PLoS One* 2016; **11**. DOI:10.1371/journal.pone.0153490.

831 Watkins E, Owens M, Cook L. Habits in Depression: Understanding and Intervention. In: Verplanken B, ed. The Psychology of Habit: Theory, Mechanisms, Change, and Contexts. Cham: Springer International Publishing, 2018: 267–84.

832 Welsh SA. Health Habits, Coping Behaviors, and Perceived Social Support in Primary Care Physicians as a Function of Level of Burnout. 2017.

833 Chang F-Y, Huang H-C, Lin K-C, Lin L-C. The effect of a music programme during lunchtime on the problem behaviour of the older residents with dementia at an institution in Taiwan. *Journal of Clinical Nursing* 2010; **19**: 939–48.

834 Clark ME, Lipe AW, Bilbrey M. USE OF MUSIC to Decrease Aggressive Behaviors in People With Dementia. *J Gerontol Nurs* 1998; **24**: 10–7.

835 Cox E, Nowak M, Buettner P. Managing Agitated Behaviour in People with Alzheimer’s Disease: The Role of Live Music. *British Journal of Occupational Therapy* 2011; **74**: 517–24.

836 Hunter L. Musical Instrument Education and Inhibitory Control in Adolescent Youth. 2018; published online July 12. DOI:10.13016/M2WW7732J.

837 Mansouri FA, Acevedo N, Illipparampil R, Fehring DJ, Fitzgerald PB, Jaberzadeh S. Interactive effects of music and prefrontal cortex stimulation in modulating response inhibition. *Scientific Reports* 2017; **7**: 1–13.

838 Alain C, Moussard A, Singer J, Lee Y, Bidelman GM, Moreno S. Music and Visual Art Training Modulate Brain Activity in Older Adults. *Front Neurosci* 2019; **13**. DOI:10.3389/fnins.2019.00182.

839 Wegner L, Flisher AJ. Leisure boredom and adolescent risk behaviour: a systematic literature review. *Journal of Child and Adolescent Mental Health* 2009; published online Nov 12. DOI:10.2989/JCAMH.2009.21.1.4.806.

840 Brizi A, Chirumbolo A, Mannetti L, Scerbo S. Health behaviours and risk taking intentions. The role of regulatory modes and self-control. *PC* 2017. DOI:10.1482/87885.

841 Ent MR, Gerend MA. Cognitive dissonance and attitudes toward unpleasant medical screenings. *J Health Psychol* 2016; **21**: 2075–84.

842 Hookway S, Johansson MF, Svensson A, Heiden B. The Problem with Problems: Reframing and Cognitive Bias in Healthcare Innovation. *The Design Journal* 2019; **22**: 553–574.

843 Hwang S, Nolan ZT, White SF, Williams WC, Sinclair S, Blair RJR. Dual neurocircuitry dysfunctions in disruptive behavior disorders: emotional responding and response inhibition. *Psychological Medicine* 2016; **46**: 1485–96.

844 Kelly MP. Cognitive biases in public health and how economics and sociology can help overcome them. *Public Health* 2019; **169**: 163–72.

845 Zapf PA, Kukucka J, Kassin SM, Dror IE. Cognitive bias in forensic mental health assessment: Evaluator beliefs about its nature and scope. *Psychology, Public Policy, and Law* 2018; **24**: 1–10.

846 Bergin DA. Leisure Activity, Motivation, and Academic Achievement in High School Students. *Journal of Leisure Research* 1992; **24**: 225–39.

847 Hebblethwaite S, Norris J. Expressions of Generativity Through Family Leisure: Experiences of Grandparents and Adult Grandchildren. *Family Relations* 2011; **60**: 121–33.

848 Dwyer JJM. Effect of perceived choice of music on exercise intrinsic motivation. *Health Values: The Journal of Health Behavior, Education & Promotion* 1995; **19**: 18–26.

849 Reid D, Hirji T. The Influence of a Virtual Reality Leisure Intervention Program on the Motivation of Older Adult Stroke Survivors: A Pilot Study. *Physical & Occupational Therapy In Geriatrics* 2004; **21**: 1–19.

850 Caldwell LL, Patrick ME, Smith EA, Palen L-A, Wegner L. Influencing Adolescent Leisure Motivation: Intervention Effects of Health Wise South Africa. *Journal of Leisure Research* 2010; **42**: 203–20.

851 Chatzisarantis NLD, Hagger MS. Effects of a Brief Intervention Based on the Theory of Planned Behavior on Leisure-Time Physical Activity Participation. *Journal of Sport and Exercise Psychology* 2005; **27**: 470–87.

852 Son J, Wilson J. Generativity and Volunteering. *Sociological Forum* 2011; **26**: 644–67.

853 Hong D-G, Jeong H-Y, Park J, Kim S. The Effect of a Leisure Motivation and Participation Restriction of Stroke Patients on Level of Leisure Activity. 2015. DOI:10.14519/jksot.2015.23.3.02.

854 Kanter JW, Puspitasari AJ, Santos MM, Nagy GA. Behavioural activation: history, evidence and promise. *The British Journal of Psychiatry* 2012; **200**: 361–3.

855 Orgeta V, Brede J, Livingston G. Behavioural activation for depression in older people: systematic review and meta-analysis. *The British Journal of Psychiatry* 2017; : bjp.bp.117.205021.

856 Rudolph KD, Bohn LE. Translating Social Motivation Into Action: Contributions of Need for Approval to Children’s Social Engagement. *Social Development* 2014; **23**: 376–94.

857 Simpson EH, Balsam PD. The Behavioral Neuroscience of Motivation: An Overview of Concepts, Measures, and Translational Applications. *Curr Top Behav Neurosci* 2016; **27**: 1–12.

858 Phillips RD, Gorton RL, Pinciotti P, Sachdev A. Promising Findings on Preschoolers’ Emergent Literacy and School Readiness In Arts-integrated Early Childhood Settings. *Early Childhood Educ J* 2010; **38**: 111–22.

859 Brown ED, Benedett B, Armistead ME. Arts enrichment and school readiness for children at risk. *Early Childhood Research Quarterly* 2010; **25**: 112–24.

860 Butt ML, Kisilevsky BS. Music Modulates Behaviour of Premature Infants Following Heel Lance. *Canadian Journal of Nursing Research Archive* 2016; **31**. https://cjnr.archive.mcgill.ca/article/view/1549 (accessed Feb 19, 2020).

861 Levy AK, Schaefer L, Phelps PC. Increasing preschool effectiveness: Enhancing the language abilities of 3- and 4-year-old children through planned sociodramatic play. *Early Childhood Research Quarterly* 1986; **1**: 133–40.

862 Fancourt D, Steptoe A. Effects of creativity on social and behavioral adjustment in 7-to 11-year-old children. *Annals of the New York Academy of Sciences* 2019; **1438**: 30–39.

863 Schneider S, Weiß M, Thiel A, *et al.* Body dissatisfaction in female adolescents: extent and correlates. *Eur J Pediatr* 2013; **172**: 373–84.

864 McPhie ML, Rawana JS. The effect of physical activity on depression in adolescence and emerging adulthood: A growth-curve analysis. *Journal of Adolescence* 2015; **40**: 83–92.

865 Barkley RA. Major life activity and health outcomes associated with attention-deficit/hyperactivity disorder. *The Journal of Clinical Psychiatry* 2002; **63**: 10–5.

866 Hansen ÅM, Hogh A, Persson R, Karlson B, Garde AH, Ørbæk P. Bullying at work, health outcomes, and physiological stress response. *Journal of Psychosomatic Research* 2006; **60**: 63–72.

867 Hertzman C, Wiens M. Child development and long-term outcomes: A population health perspective and summary of successful interventions. *Social Science & Medicine* 1996; **43**: 1083–95.

868 Irwin LG, Siddiqi A, Hertzman G. Early child development: a powerful equalizer. Human Early Learning Partnership (HELP) Vancouver, BC, 2007.

869 Nigg JT. Attention-deficit/hyperactivity disorder and adverse health outcomes. *Clinical Psychology Review* 2013; **33**: 215–28.

870 Rowland AS, Lesesne CA, Abramowitz AJ. The epidemiology of attention-deficit/hyperactivity disorder (ADHD): A public health view. *Mental Retardation and Developmental Disabilities Research Reviews* 2002; **8**: 162–70.

871 Csikszentmihalyi M, LeFevre J. Optimal experience in work and leisure. *Journal of personality and social psychology* 1989; **56**: 815.

872 Heintzman P, Mannell RC. Spiritual Functions of Leisure and Spiritual Well-Being: Coping with Time Pressure. *Leisure Sciences* 2003; **25**: 207–30.

873 Hull RB, Michael SE, Walker GJ, Roggenbuck JW. Ebb and flow of brief leisure experiences. *Leisure Sciences* 1996; **18**: 299–314.

874 Johnson AJ, Glover TD, Stewart WP. Attracting Locals Downtown: Everyday Leisure as a Place-Making Initiative. *Journal of Park and Recreation Administration* 2014; **32**. https://js.sagamorepub.com/jpra/article/view/5724 (accessed Feb 25, 2020).

875 Kruger LE. Recreation as a path for place making and community building. *Leisure/Loisir* 2006; **30**: 383–92.

876 Kyle G, Graefe A, Manning R, Bacon J. An Examination of the Relationship between Leisure Activity Involvement and Place Attachment among Hikers Along the Appalachian Trail. *Journal of Leisure Research* 2003; **35**: 249–73.

877 Mannell RC, Zuzanek J, Larson R. Leisure States and “Flow” Experiences: Testing Perceived Freedom and Intrinsic Motivation Hypotheses. *Journal of Leisure Research* 1988; **20**: 289–304.

878 Mattijssen TJM, van der Jagt APN, Buijs AE, Elands BHM, Erlwein S, Lafortezza R. The long-term prospects of citizens managing urban green space: From place making to place-keeping? *Urban Forestry & Urban Greening* 2017; **26**: 78–84.

879 McClinchey KA. Social sustainability and a sense of place: harnessing the emotional and sensuous experiences of urban multicultural leisure festivals. *Leisure/Loisir* 2017; **41**: 391–421.

880 Goodman WK, Geiger AM, Wolf JM. Leisure activities are linked to mental health benefits by providing time structure: comparing employed, unemployed and homemakers. *J Epidemiol Community Health* 2017; **71**: 4–11.

881 Davies S, Filippopoulos P. Changes in Psychological Time Perspective During Residential Addiction Treatment: A Mixed-Methods Study. *Journal of Groups in Addiction & Recovery* 2015; **10**: 249–70.

882 Eyles J, Williams A. Sense of place, health and quality of life. Ashgate Publishing, Ltd., 2008.

883 Gesler WM, Kearns RA, Kearns RA. Culture/Place/Health. Routledge, 2005 DOI:10.4324/9780203996317.

884 Macintyre S, Ellaway A, Cummins S. Place effects on health: how can we conceptualise, operationalise and measure them? *Social Science & Medicine* 2002; **55**: 125–39.

885 Sommers J, Vodanovich SJ. Boredom proneness: Its relationship to psychological- and physical-health symptoms. *Journal of Clinical Psychology* 2000; **56**: 149–55.

886 Weissinger E. Effects of Boredom on Self-Reported Health. *Loisir et Société / Society and Leisure* 1995; **18**: 21–32.

887 Williams LD and A. A Sense of Place, A Sense of Well-being. Sense of Place, Health and Quality of Life. 2016; published online Dec 5. DOI:10.4324/9781315243474-12.

888 Anshel A, Kipper DA. The Influence of Group Singing on Trust and Cooperation. *J Music Ther* 1988; **25**: 145–55.

889 Coleman D, Iso-Ahola SE. Leisure and Health: The Role of Social Support and Self-Determination. *Journal of Leisure Research* 1993; **25**: 111–28.

890 Jasper JM. The Art of Moral Protest: Culture, Biography, and Creativity in Social Movements. University of Chicago Press, 2008.

891 Mahoney JL, Stattin H. Leisure activities and adolescent antisocial behavior: The role of structure and social context. *Journal of Adolescence* 2000; **23**: 113–27.

892 Toepoel V. Ageing, Leisure, and Social Connectedness: How could Leisure Help Reduce Social Isolation of Older People? *Soc Indic Res* 2013; **113**: 355–72.

893 Staiano AE, Abraham AA, Calvert SL. Adolescent exergame play for weight loss and psychosocial improvement: A controlled physical activity intervention. *Obesity* 2013; **21**: 598–601.

894 Ansari S. Social capital and collective efficacy: Resource and operating tools of community social control. *Journal of Theoretical & Philosophical Criminology* 2013; **5**: 75.

895 Brown P, Zavestoski S, McCormick S, Mayer B, Morello-Frosch R, Gasior Altman R. Embodied health movements: new approaches to social movements in health. *Sociology of health & illness* 2004; **26**: 50–80.

896 Brown P, Zavestoski S. Social movements in health: an introduction. *Sociology of health & illness* 2004; **26**: 679–694.

897 Lepper MR. 12 Social-control processes and the internalization of social values: an. 1983.

898 Umberson D. Gender, marital status and the social control of health behavior. *Social science & medicine* 1992; **34**: 907–917.

899 Angus J. A review of evaluation in community based art for health activity in the UK | Repository for Arts and Health Resources. 2002. https://www.artshealthresources.org.uk/docs/a-review-of-evaluation-in-community-based-art-for-health-activity-in-the-uk/ (accessed July 27, 2020).

900 Carson AJ, Chappell NL, Knight CJ. Promoting Health and Innovative Health Promotion Practice Through a Community Arts Centre. *Health Promotion Practice* 2007; **8**: 366–74.

901 Coalter F. Leisure studies, leisure policy and social citizenship: the failure of welfare or the limits of welfare? *Leisure Studies* 1998; **17**: 21–36.

902 Dobbinson SJ, Hayman JA, Livingston PM. Prevalence of health promotion policies in sports clubs in Victoria, Australia. *Health Promot Int* 2006; **21**: 121–9.

903 Kokko S, Kannas L, Villberg J, Ormshaw M. Health promotion guidance activity of youth sports clubs. *Health Education* 2011; **111**: 452–463.

904 McQueen-Thomson D. Promoting Mental Health & Wellbeing through Community & Cultural Development: A Review of Literature focussing on Community Festivals & Celebrations | Arts Health and Wellbeing. 2004. https://www.artshealthandwellbeing.org.uk/resources/research/promoting-mental-health-wellbeing-through-community-cultural-development-review-l (accessed July 27, 2020).

905 Putland C. Lost in Translation: The Question of Evidence Linking Community-based Arts and Health Promotion. *J Health Psychol* 2008; **13**: 265–76.

906 DiClemente RJ, Crosby RA, Kegler MC. Emerging Theories in Health Promotion Practice and Research. John Wiley & Sons, 2009.

907 Harris JS, Fries J. The health effects of health promotion. *Health Promotion in the Workplace Albany, NY: Delmar Publishers Inc* 2002; : 1–22.

908 Martin A, Sanderson K, Cocker F. Meta-analysis of the effects of health promotion intervention in the workplace on depression and anxiety symptoms. *Scandinavian Journal of Work, Environment & Health* 2009; **35**: 7–18.

909 Mittelmark MB. Promoting social responsibility for health: health impact assessment and healthy public policy at the community level. *Health Promot Int* 2001; **16**: 269–74.

910 Cox J. Leisure property trends: Consumer spending and leisure futures. *J Retail Leisure Property* 2002; **2**: 180–90.

911 DyckFehderau D, Holt NL, Ball GD, Alexander First Nation Community, Willows ND. Feasibility study of asset mapping with children: identifying how the community environment shapes activity and food choices in Alexander First Nation. *Rural Remote Health* 2013; **13**: 2289.

912 Nichols G, Forbes D, Findlay-King L, Macfadyen G. Is the Asset Transfer of Public Leisure Facilities in England an Example of Associative Democracy? *Administrative Sciences* 2015; **5**: 71–87.

913 Sickles RC, Yazbeck A. On the Dynamics of Demand for Leisure and the Production of Health. *Journal of Business & Economic Statistics* 1998; **16**: 187–97.

914 De Vries S, Verheij RA, Groenewegen PP, Spreeuwenberg P. Natural environments—healthy environments? An exploratory analysis of the relationship between greenspace and health. *Environment and planning A* 2003; **35**: 1717–1731.

915 Lachowycz K, Jones AP. Towards a better understanding of the relationship between greenspace and health: Development of a theoretical framework. *Landscape and Urban Planning* 2013; **118**: 62–9.

916 Maybery D, Pope R, Hodgins G, Hitchenor Y, Shepherd A. Resilience and Well-Being of Small Inland Communities: Community Assets as Key Determinants. *Rural Society* 2009; **19**: 326–39.

917 Anígilájé EA, Dabit OJ, Tyovenda RK, *et al.* Effects of leisure activities and psychosocial support on medication adherence and clinic attendance among children on antiretroviral therapy. *HIV AIDS (Auckl)* 2014; **6**: 127–37.

918 Mak HW, Fancourt D. Reading for pleasure in childhood and adolescent healthy behaviours: Longitudinal associations using the Millennium Cohort Study. *Preventive Medicine* 2020; **130**: 105889.

919 Menec VH, Chipperfield JG. Remaining Active in Later Life: The Role of Locus of Control in Seniors’ Leisure Activity Participation, Health, and Life Satisfaction. *J Aging Health* 1997; **9**: 105–25.

920 Nasermoaddeli A, Sekine M, Kumari M, Chandola T, Marmot M, Kagamimori S. Association of Sleep Quality and Free Time Leisure Activities in Japanese and British Civil Servants. *Journal of Occupational Health* 2005; **47**: 384–90.

921 Florindo AA, Guimarães VV, Cesar CLG, Barros MB de A, Alves MCGP, Goldbaum M. Epidemiology of Leisure, Transportation, Occupational, and Household Physical Activity: Prevalence and Associated Factors. *Journal of Physical Activity and Health* 2009; **6**: 625–32.

922 Triguero-Mas M, Dadvand P, Cirach M, *et al.* Natural outdoor environments and mental and physical health: Relationships and mechanisms. *Environment International* 2015; **77**: 35–41.

923 Osman M, Eacott B, Willson S. Arts-based interventions in healthcare education. *Medical Humanities* 2018; **44**: 28–33.

924 Johnson G, Otto D, Clair AA. The Effect of Instrumental and Vocal Music on Adherence to a Physical Rehabilitation Exercise Program with Persons who are Elderly. *J Music Ther* 2001; **38**: 82–96.

925 Cobbett S. Reaching the hard to reach: quantitative and qualitative evaluation of school-based arts therapies with young people with social, emotional and behavioural difficulties. *Emotional and Behavioural Difficulties* 2016; **21**: 403–15.

926 Jones M, Kimberlee R, Deave T, Evans S. The Role of Community Centre-based Arts, Leisure and Social Activities in Promoting Adult Well-being and Healthy Lifestyles. *International Journal of Environmental Research and Public Health* 2013; **10**: 1948–62.

927 Spring B, Moller AC, Coons MJ. Multiple health behaviours: overview and implications. *J Public Health (Oxf)* 2012; **34**: i3–10.

928 Steptoe A. Handbook of Behavioral Medicine: Methods and Applications. Springer Science & Business Media, 2010.

929 Akker OBAVD, Lees S. Leisure Activities and Adolescent Sexual Behaviour. *Sex Education* 2001; **1**: 137–47.

930 Andersson A, Andersson C, Holmgren K, Mårdby A-C, Hensing G. Participation in leisure activities and binge drinking in adults: Findings from a Swedish general population sample. *Addiction Research & Theory* 2012; **20**: 172–82.

931 Krumm-Merabet C, Meyer TD. Leisure activities, alcohol, and nicotine consumption in people with a hypomanic/hyperthymic temperament. *Personality and Individual Differences* 2005; **38**: 701–12.

932 Moore S, Ohtsuka K. The Structure of Young People’s Leisure and Their Gambling Behaviour. *Behaviour Change* 2000; **17**: 167–77.

933 Piko BF, Vazsonyi AT. Leisure activities and problem behaviours among Hungarian youth. *Journal of Adolescence* 2004; **27**: 717–30.

934 Wang H, Singhal A. East Los High: Transmedia Edutainment to Promote the Sexual and Reproductive Health of Young Latina/o Americans. *Am J Public Health* 2016; **106**: 1002–10.

935 Lakerveld J, Bot SDM, van der Ploeg HP, Nijpels G. The effects of a lifestyle intervention on leisure-time sedentary behaviors in adults at risk: The Hoorn Prevention Study, a randomized controlled trial. *Preventive Medicine* 2013; **57**: 351–6.

936 Buck D, Frosini F. Clustering of Unhealthy Behaviours Over Time: Implications for Policy and Practice. King’s Fund, 2012.

937 Bates V. ‘Humanizing’ healthcare environments: architecture, art and design in modern hospitals. *Design for Health* 2018; **2**: 5–19.

938 Camic PM, Chatterjee HJ. Museums and art galleries as partners for public health interventions. *Perspect Public Health* 2013; **133**: 66–71.

939 Horwitz P, Kretsch C, Jenkins A, *et al.* Contribution of biodiversity and green spaces to mental and physical fitness, and cultural dimensions of health. World Health Organization and Secretariat of the Convention on Biological Diversity, 2015. https://ore.exeter.ac.uk/repository/handle/10871/19908 (accessed Feb 19, 2020).

940 Lamont M, Beljean S, Clair M. What is missing? Cultural processes and causal pathways to inequality. *Socioecon Rev* 2014; **12**: 573–608.

941 Parker A, Marturano N, O’Connor G, Meek R. Marginalised youth, criminal justice and performing arts: young people’s experiences of music-making. *Journal of Youth Studies* 2018; **21**: 1061–76.

942 Parkinson C, White M. Inequalities, the arts and public health: Towards an international conversation. *Arts & Health* 2013; **5**: 177–89.

943 Sonke J, Pesata V, Arce L, Carytsas FP, Zemina K, Jokisch C. The effects of arts-in-medicine programming on the medical-surgical work environment. *Arts & Health* 2015; **7**: 27–41.

944 Sonke J, Pesata V, Nakazibwe V, *et al.* The Arts and Health Communication in Uganda: A Light Under the Table. *Health Communication* 2018; **33**: 401–8.

945 Sonke J, Pesata V. The arts and health messaging: Exploring the evidence and lessons from the 2014 Ebola outbreak. *BMJ Outcomes* 2015; **1**: 36–41.

946 Heijink R, Koolman X, Westert GP. Spending more money, saving more lives? The relationship between avoidable mortality and healthcare spending in 14 countries. *Eur J Health Econ* 2013; **14**: 527–38.

947 Jha AK, Orav EJ, Li Z, Epstein AM. The Inverse Relationship Between Mortality Rates And Performance In The Hospital Quality Alliance Measures. *Health Affairs* 2007; **26**: 1104–10.

948 Schoen C, Osborn R, Doty MM, Bishop M, Peugh J, Murukutla N. Toward Higher-Performance Health Systems: Adults’ Healthcare Experiences In Seven Countries, 2007. *Health Affairs* 2007; **26**: w717–34.

949 Volandes AE, Paasche-Orlow MK. Health Literacy, Health Inequality and a Just Healthcare System. *The American Journal of Bioethics* 2007; **7**: 5–10.

950 Shiell A, Hawe P, Gold L. Complex interventions or complex systems? Implications for health economic evaluation. *Bmj* 2008; **336**: 1281–1283.

951 Crawford DW, Jackson EL, Godbey G. A hierarchical model of leisure constraints. *Leisure Sciences* 1991; **13**: 309–20.

952 Godbey G, Crawford D, Shen X. Assessing Hierarchical Leisure Constraints Theory after Two Decades. *Journal of Leisure Research* 2010; **42**: 111–34.

953 Kelly JR. Situational and Social Factors in Leisure Decisions. *The Pacific Sociological Review* 1978; **21**: 313–30.

954 Michie S, van Stralen MM, West R. The behaviour change wheel: a new method for characterising and designing behaviour change interventions. *Implement Sci* 2011; **6**: 42.

955 Marmot M, Allen J, Bell R, Bloomer E, Goldblatt P. WHO European review of social determinants of health and the health divide. *The Lancet* 2012; **380**: 1011–29.
